# Novel Dipyridinium Lipophile-Based Ionic Liquids Tethering Hydrazone Linkage: Design, Synthesis and Antitumorigenic Study

**DOI:** 10.3390/ijms221910487

**Published:** 2021-09-28

**Authors:** Salsabeel Al-Sodies, Nadjet Rezki, Fawzia Faleh Albelwi, Mouslim Messali, Mohamed R. Aouad, Sanaa K. Bardaweel, Mohamed Hagar

**Affiliations:** 1 Department of Chemistry, Faculty of Science, Taibah University, Al-Madinah Al-Munawarah 30002, Saudi Arabia; s7l_88@hotmail.com (S.A.-S.); ffs.chem334@gmail.com (F.F.A.); aboutasnim@yahoo.fr (M.M.); aouadmohamedreda@yahoo.fr (M.R.A.); 2 Department of Pharmaceutical Sciences, Faculty of Pharmacy, University of Jordan, Amman 11942, Jordan; 3 Chemistry Department, Faculty of Science, Alexandria University, Alexandria 21321, Egypt; mohamedhaggar@gmail.com

**Keywords:** ionic liquids, hydrazones, amphiphiles, lung cancer, metastasis, tumor colonization

## Abstract

Novel dicationic pyridinium ionic liquids tethering amphiphilic long alkyl side chains and fluorinated counter anions have been successfully synthesized by means of the quaternization of the dipyridinium hydrazone through its alkylation with different alkyl halides. The resulting halogenated di-ionic liquids underwent a metathesis reaction in order to incorporate some fluorinated counter anions in their structures. The structures of all the resulting di-ionic liquids were characterized by several spectroscopic experiments. The antitumorigenic activities of the investigated compounds were further studied against three different human lung cancer cell lines. Compared to the standard chemotherapeutic agent, cisplatin, the synthesized di-ionic liquids exerted equal, even more active, moderate, or weak anticancer activities against the various lung cancer cell lines under investigation. The observed anticancer activity appears to be enhanced by increasing the length of the aliphatic side chains. Moreover, dicationic pyridinium bearing a nine carbon chain as counter cation and hexafluoro phosphate and/or tetrafluoro bororate as counter anion were selected for further evaluation and demonstrated effective and significant antimetastatic effects and suppressed the colonization ability of the lung cancer cells, suggesting a therapeutic potential for the synthesized compounds in lung cancer treatment.

## 1. Introduction

Lung cancer (LC) is one of the most common cancers in both genders and appears to be a leading cause of cancer mortality globally [1,2]. Lung cancer is categorized depending on histopathological characteristics: small cell lung cancer (SCLC) and non-small cell lung cancer (NSCLC), which is the most commonly diagnosed type of lung cancer, accounting for 85% of all lung cancer cases [3]. The incidence of LC fluctuates by age, gender, and race in different human populations.

Effective treatments for lung cancer vary depending on the histologic type of cancer, the clinical stage, and the patient’s functional status [4]. Radiotherapy, targeted therapy, chemotherapy, and immunotherapy are common effective treatments, with clear limitations for each type of therapy and high tumor relapse rates among patients [5]. While surgery is the first line treatment option in the early stages of lung cancers, it is not effective when metastasis to other organs occurs [4]. Lung cancer patients have a five-year survival rate of less than 20% throughout all stages [1,2]. As a consequence, it is crucial to examine new, safe, and effective therapeutic options against lung cancer.

Over the last decade, evidence has emerged to support the efficacy of ionic liquids (ILs) as druggable molecules in a molten state [6]. The chemistry of ILs has become a hot topic since it is concerned with the design and development of newer scaffolds, as well as the ability to understand their medical properties and feature areas that guide their design and synthesis process [7,8].

Recently, significant effort has been expended on the design and synthesis of these fascinating molecules, which have revolutionized virtually all aspects of daily life [6]. ILs can now be sketched and constructed from bulky organic cations paired with either organic or inorganic anions to accomplish a structural framework with a particular melting point, density and lower toxicity [9,10] along with increasing profits in their medical application due to their safety and stability properties: nonflammable and nonvolatile, negligible vapor pressure, high ionic conductivity and thermal stability, easy recyclability and miscibility with aqueous and organic solvents [11,12,13,14,15].

As part of our ongoing progress in the field of potent ionic liquid frameworks bearing pyridine-hydrazone [16,17,18,19], these features have significantly improved our structural and functional expertise. Thus, we attempt in the present work to provide the design, synthesis and characterization of an array of dicationic pyridinium hydrazone incorporating long alkyl side chains.

Further to that, we assessed the cytotoxic properties of the targeted ILs against three different human lung cancer cell lines and established their antitumorigenic activity profiles, not only as cancer cell growth inhibitors but also as antimigratory and anticolonization substances. Upon careful inspection of the existing literature, this study is the first report that has investigated the ability of ILs to modulate cancer cells’ metastasis and their ability to colonize.

### The Rational Study

Ionic liquids (ILs) are ionic materials with distinct properties that make them a promising contender in a range of fields, particularly pharmaceutical sciences. ILs are classified into three generations based on their properties and characteristics [20]. The third generation of ILs have been reviewed and documented as fascinating active pharmaceutical ingredients (APIs) and have been widely used to develop biologically active candidate-based IL building blocks. Recently, the majority of research has focused mainly on the physical and chemical characteristics, and the toxicity and biological behavior of ILs have gained prominence as two of the most controversial issues in this area. Thereby, novel ILs have been developed using biologically active ions; however, the primary motivation for this research has been to investigate the use of well-known low-toxicity ions to establish ILs with the necessary details [21,22,23,24,25].

Melting temperature and solubility are significant considerations in the pharmaceutical industry because they are regularly monitored and have the potential to impact drug processing and bioavailability [26,27,28]. This is a useful approach for varying the composition of a drug using ionizable functional groups in order to overcome the undesirable aspects in the parent molecule. A drug’s quality, safety, and performance are all impacted only by salt structure. The ion pair chosen could have a major impact on the pharmacokinetics of a medication candidate. That is one of the reasons regulatory agencies have launched the categorizing of novel salts of a licensed medicine as a new chemical entity [28].

The generation and structural analysis of the demanded medications were used to generate such (ILs) as APIs and became an effective approach for addressing the challenges of the pharmaceutical industry.

Despite the fact that several treatment options are available for lung cancer, high tumor relapse rates and resistance to chemotherapy are prevalent. Lung cancer patients have a five-year survival rate of less than 20%. As a consequence, it is imperative to develop new, safe, and effective lung cancer therapeutic approaches.

**Figure 1:**
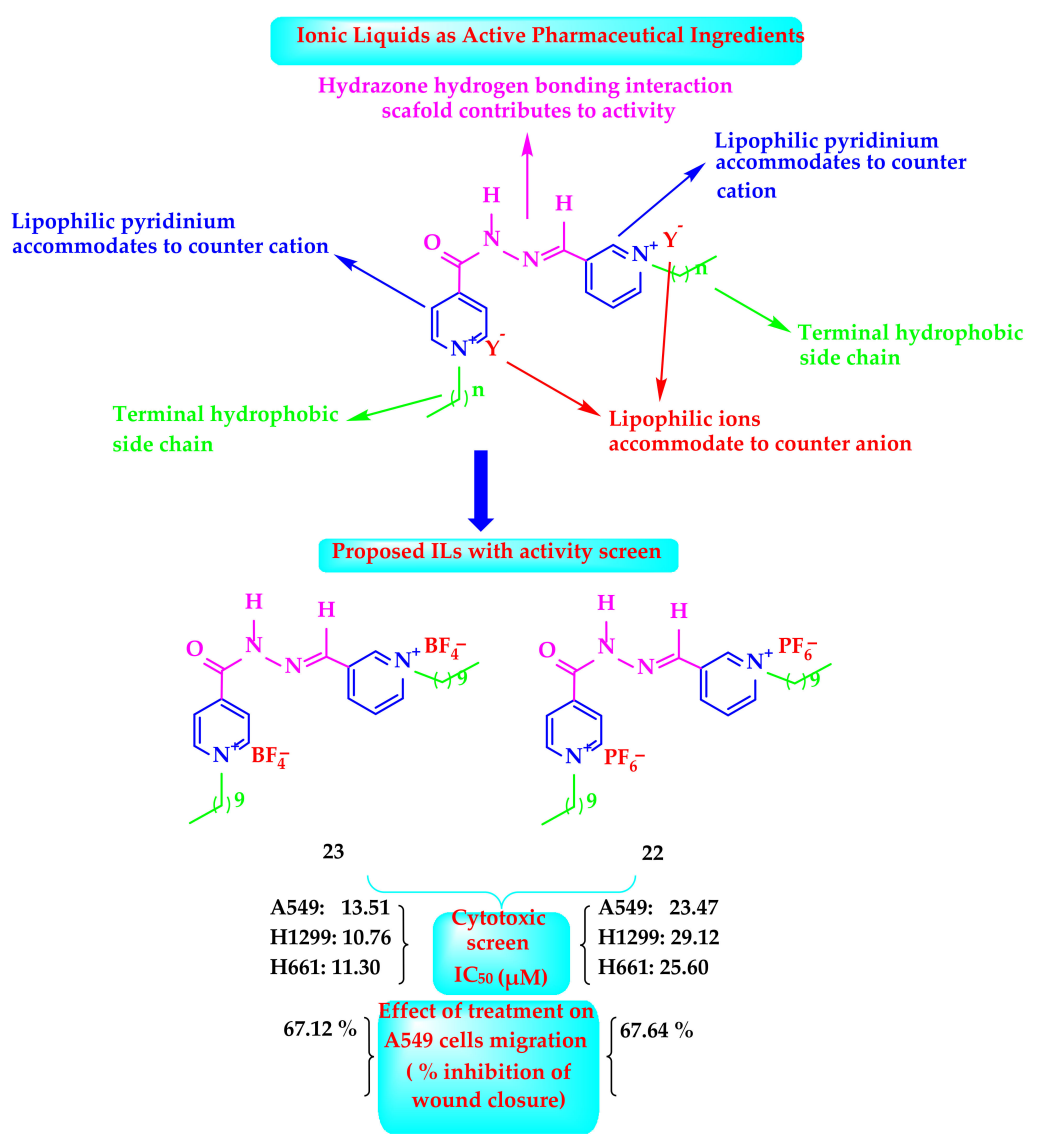
Schematic representation of the proposed research.

We proposed to construct a library of dipyridinium ionic liquids tethering numerous long alkyl side chains and some fluorinated anions and test them for antitumorigenic activity to find the obvious ideal candidate of the new advanced third generation of IL-based active units with promising antiproliferative activity (Figure 1).

## 2. Results and Discussion

### 2.1. Synthesis and Characterization

**Scheme 1:**
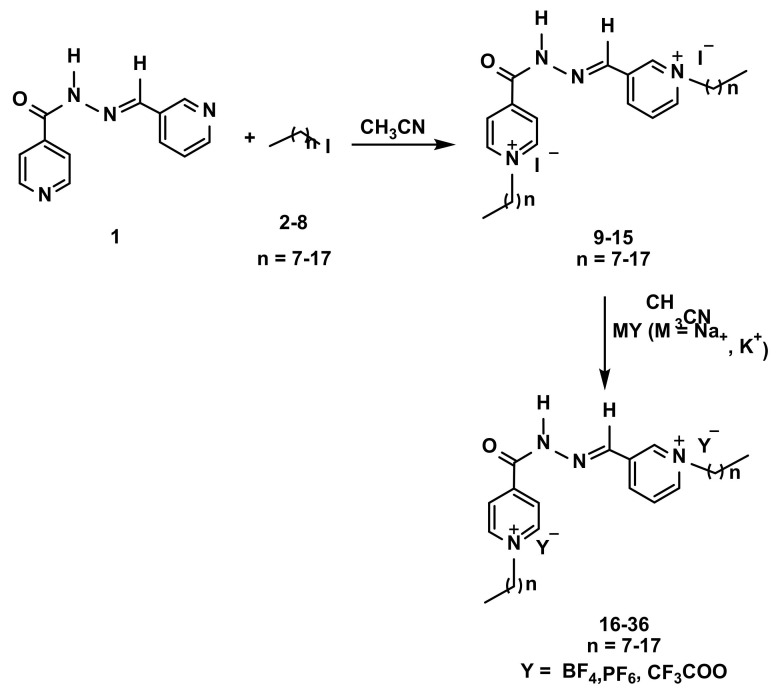
Synthetic route for the synthesis of dicationic pyridinium lipophile-based ionic liquids incorporating hydrazone anion **9**–**36**.

The synthetic pathway adopted in this study is depicted in Scheme 1. In general, ionic liquids can be synthesized by quaternizing nitrogen-containing molecules followed by a metathetical reaction. The dipyridinium hydrazone **1** was selected as the starting material for the synthesis of the designed DiILs. It was noticeable that the dipyridinium hydrazone **1** was prepared through the condensation of 3-pyridinecarboxaldehyde and isonicotinic acid hydrazide in refluxing ethanol in the presence of a few drops of hydrochloric acid, according to reported literature [29] (Scheme 1).

In refluxing acetonitrile (CH_3_CN), the two nitrogen pyridine atoms of the starting dipyridine hydrazone **1** were bis-alkylated with two equivalents of some selected alkyl iodides with eight to eighteen carbons in their aliphatic (Scheme 1), yielding the targeted dipyridinium ILs **9**–**15** bearing a hydrophobic side chain as counter cation and iodide as counter anion as listed in Table 1. The success of such a quaternization reaction was evidenced by the proton and carbon nuclear magnetic resonance (^1^H and ^13^C NMR) experiments. Thus, the appearance of diagnostic triplet and multiplet around δ_H_ 0.84 ppm and 4.58–4.69 ppm attributed to the lateral C**H_3_** and NC**H_2_**, respectively, confirmed the incorporation of the alkyl chain in their structure. The spectroscopic data also revealed extra methylene protons in the aliphatic area belonging to the alkyl protons. The carbons of the same groups (**C**H_3_ and N**C**H_2_) were observed in their ^13^C NMR spectra at δ_C_ 14.43–14.44 ppm and δ_C_ 61.51–61.61 ppm, respectively. Additional aliphatic carbons were also resonated at their expected chemical shifts (See experimental section).

The synthesized dicationic halogenated pyridinium ionic liquids **9**–**15** were then subjected to the metathetical process in order to displace the iodide anion and incorporate the new fluorinated counter anions (PF_6_^−^, BF_4_^−^, CF_3_COO^−^), as detailed in Table 1. In this respect, the thermal treatment of DiILs **9**–**15** with some fluorinated metal salts, in acetonitrile for 16 h resulted in the elaboration of the desired dicationic pyridinium hydrazones tethering the targeted fluorinated counter anions **16**–**36** (Scheme 1) in good yields (88–96%). The structures of these task-specific DiILs **16**–**36** were deduced based on their spectroscopic results. Due to the similarity of the proton and carbon signals on the ^1^H and ^13^C NMR of **16**–**36** compared to their halogenated analogues **9**–**15**, no difference was detected.

The architectural distinction between the resulting material **16**–**36** and their halogenated building blocks **9**–**15** was based on the NMR of ^19^F, ^31^P, and ^11^B and mass spectroscopy, to support the tethering of fluorinated counter anions (PF_6_, BF_4_, and CF_3_COO). Hence, the presence of the hexafluorophosphate (PF_6_^−^) in DiILs **16**, **19, 22, 25**, **28, 31** and **34** was confirmed using ^31^P and ^19^F NMR analyses. Thus, the exhibition of a distinct multiplet in their ^31^P NMR spectra between δ_P_ −153.02 and −135.45 ppm supported their structure. The investigation of the ^19^F NMR spectra of the same products showed the presence of a new doublet between δ_F_ −69.19 and −69.18 ppm, which was assigned to the hexafluorine anion (PF_6_^−^). On the other hand, the ^11^B and ^19^F NMR experiments proved the formation of dicationc ionic liquids **17**, **20, 23, 26**, **29, 32** and **35** comprising BF_4_^−^ in their structures. As a result, the analysis of ^11^B NMR data showed a multiplet arround δ_B_ −1.39 and −1.23 ppm, revealing that the boron atom existed in the form of tetrafluoroborate counter-anion (BF_4_^−^). In addition, their ^19^F NMR spectra exhibited two doublets at δ_F_ −148.21 and −148.14 ppm, respectively, which also supported the encompassing of tetrafluoroborate anion as counter anion in such structural ILs.

Moreover, the ^19^F NMR analysis was used to examine the architectural characterization of dipyridinium ionic liquids **18**, **21, 24, 27, 30, 33** and **36** containing trifluoroacetate (CF_3_COO^−^), that disclosed the existence of a distinctive singlet between δ_F_ −73.65 and −73.57 ppm.

### 2.2. Biological Assay

#### 2.2.1. Effect of Compounds on the Viability of Lung Cancer Cells

To evaluate the effect of the examined series on the viability of different lung cancer cells, MTT assay was conducted after exposing A549, H1299, and H661 cell lines to increasing concentrations of each compound for 48 h. The results showed potent cytotoxic activity, as treated cells had lower cell viability than untreated control cells. Table 2 and Supplementary Figure S92 (see Supplementary Materials) show the 50% inhibitory concentration (IC50) values for the compounds under investigation in A549, H1299, and H661 lung cancer cells. The same table also displays the impact of treatment on the viability of noncancerous cells, fibroblast cells, after 48 h of treatment.

#### 2.2.2. Effect of Treatment with the Compounds on Migration of Lung Cancer

**Figure 2:**
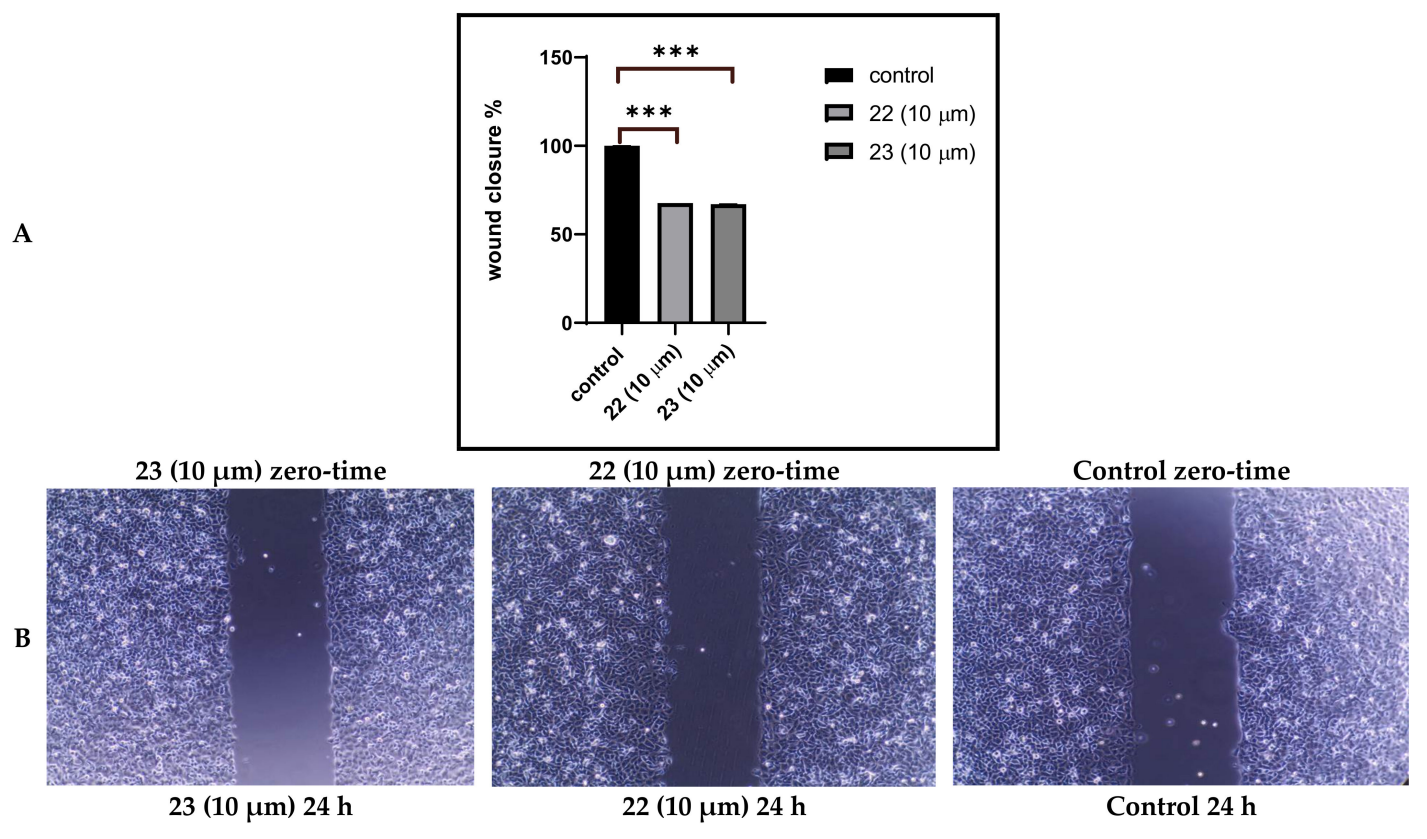

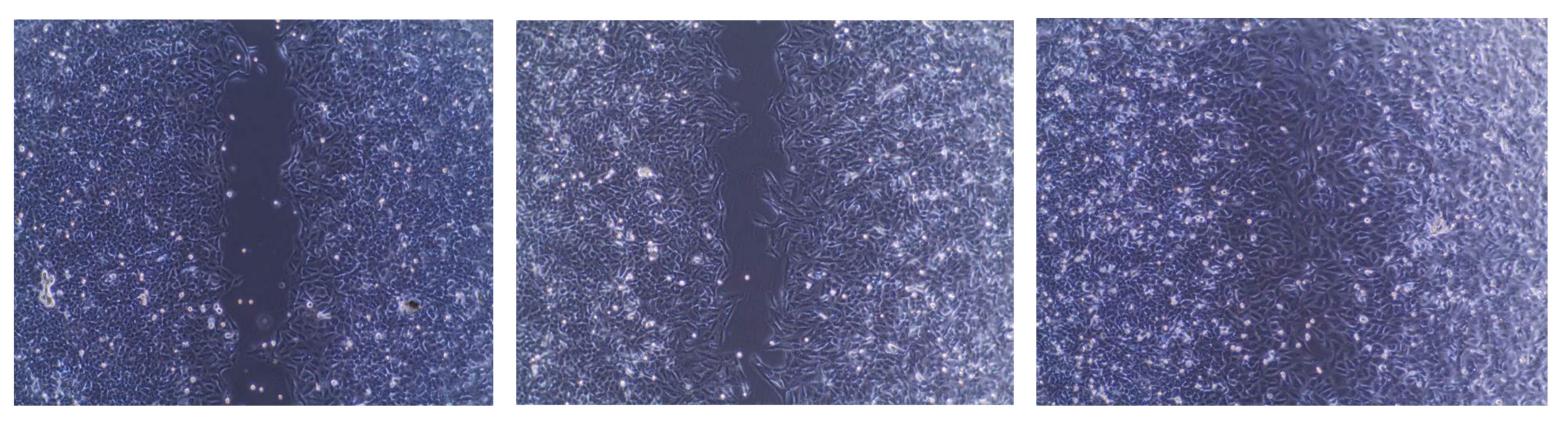
(**A**) Effect of treatment on **A549** cell migration column representation. (**B**) Cell 2D migration photos captured by 5.0 Mega Cmos camera at 4 × magnification. Data is presented as percentage wound closure at 24 h of treatment relative to wound width at time 0.

The wound healing assay was performed in A549 cells to evaluate the effect of the synthesized series on lung cancer cell migration, as shown in Table 3. The wound of the control sample was completely closed after 24 h. Compounds **22** or **23** were found to be the most effective against all cell lines tested and were thus investigated further. Cell migration was inhibited after 24 h of treatment with compounds **22** or **23** compared to untreated control cells. At 10 µM concentration, treatment of cells with each investigated compound resulted in approximately 67% inhibition of wound closure (Figure 2).

#### 2.2.3. Effect of Treatment with the Compounds on Colony-Formation Ability of Lung Cancer Cells

**Figure 3:**
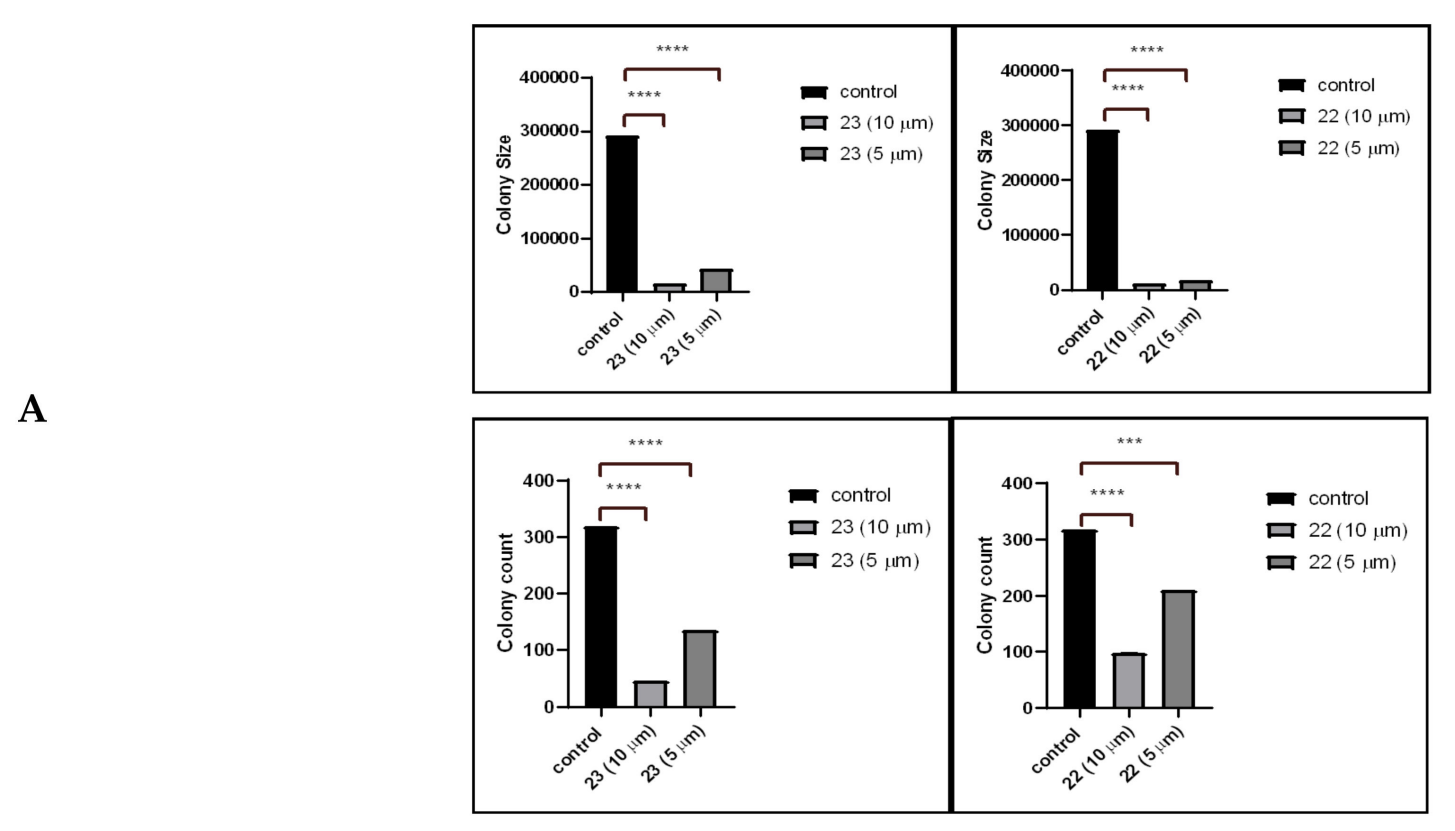

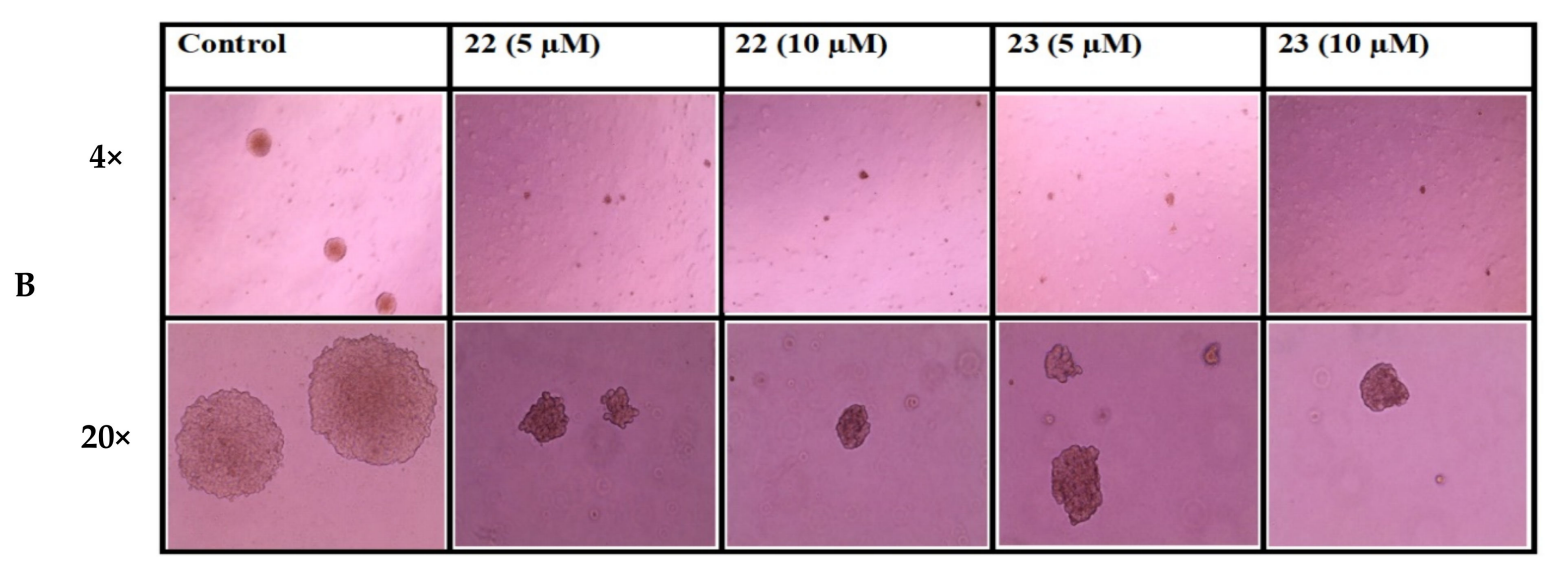
(**A**) Effect of treatment on **A549** cells colony size and count in column representation. (**B**) Pictures of the colonies were captured by 5.0 Mega Cmos camera at 4× and 20× magnification The colony-forming assay was performed by seeding equal amount (1 × 10^4^) of cells treated with IC_50_ and sub-IC_50_ concentrations of compounds 22 and 23 in soft agar in 6-well tissue culture plates. Cells were photographed after 12 days.

To investigate the effect of treatment with either potent compound **22** or **23** at two different concentrations (5 and 10 µM) on the colony-forming capability of lung cancer cells, A549 cells were treated with either concentration for 24 h. Afterwards, cells were grown on soft agar. As shown in Table 4, treatment with both compounds appears to inhibit cell colony formation by reducing the number and size of colonies compared to the control of untreated cells. Representative images taken at day 12 are shown in Figure 3.

### 2.3. Discussion

In consideration of any potential antitumor activity that could be related to the synthesized series, the growth inhibition of lung cancer cell lines (A549, H1299, H661) induced by treatment with each compound for 48 h was evaluated. The results demonstrated that the DiIL **14** harboring a 16 carbon alkyl chain substitution exerted the most potent antiproliferative effect against all examined cell lines when compared to analogues with shorter or longer alkyl chain substitutions. The derivatives **23** and **27** with alkyl side chains of 10 carbons and 12 carbons, respectively, were more effective as antitumor agents relative to their analogues harboring alkyl side chains of different lengths. The IC_50_ values (micromolar) are presented in Table 2. Relative to the standard chemotherapeutic agent cisplatin, which demonstrated IC_50_ values of 23, 18, and 32 µM against A549, H1299, and H661 cell lines, respectively, the examined compounds appear to have equal, even more active, moderate, and weak anticancer activities against the different lung cancer cells lines under investigation. Several reports in the literature have often shown that the cytotoxic effects of ILs to cancer cells are linked to the length of the substituted alkyl side chain [30]. Most commonly, DiILs with C-1 to C-18 alkyl side chains attached to various anions exhibit improved toxic effects with the extending alkyl side chain length.

Interestingly, the synthesized series demonstrated less cytotoxic effects against the normal human dermal cell line fibroblasts, revealing a unique mechanism of the observed antitumor activity. The ability of lung cancer cells to migrate between edges and close an artificial wound was assessed within 24 h of treatment to further investigate the antitumorigenic activity of the investigated DiILs. The results indicate that treatment with sub-IC_50_ concentrations of DiILs effectively suppressed the capability of the examined cancer cells to move and seal a wound, suggesting that treatment may potentially inhibit lung cancer metastasis, a hallmark of lung cancer that increases the rate of cancer replacement and chemotherapy failure. Future studies should shed light on the mechanism underlying the potential inhibition of the multistep process of invasion and metastasis.

Impactful colonization of distant organs by circulating tumor cells (CTC) is an essential process that takes place before metastatic growth. Nonetheless, factors triggering tumor dissemination and distant colonization are still unclear. According to the observed results, two of the examined DiILs have shown potent and significant inhibition of A549 cancer cell-colonization ability, measured by the number of colonies formed and the size of one formed colony, in a dose-dependent manner, suggesting a potential therapeutic utility for the targeted DiILs.

## 3. Materials and Methods

### 3.1. Chemistry

The resulting NMR spectra were obtained with a Bruker spectrometer (and 400 Brucker, Fällanden, Switzerland) using Tetramethylsilane (TMS) as an internal standard (0.00 ppm). A LCMS/MS impact II was used to perform high resolution mass spectroscopy (HRMS).

#### 3.1.1. General Procedures for the Synthesis of **9**–**15**

A solution of dipyridine hydrazone **1** (1 mmol) in CH_3_CN (25 mL) and some selected aliphatic iodide-bearing C_8_ to C_18_ carbonic chain (2.2 mmol) was refluxed for 4–6 h. The excess of solvent was evaporated at low pressure and the resulting product was collected by filtration and/or chloroform extraction to obtain the required compounds **9**–**15**.

##### Characterization of 1-Octyl-3-((2-(1-octylpyridin-1-ium-4-carbonyl)hydrazono)methyl)pyridin-1-ium Iodide (**9**)

Yield: 88%. ^1^H NMR (DMSO-*d*_6_, 400 MHz): δ_H_ = 0.84 (t, 6H, *J* = 8 Hz, 2 × C**H_3_**), 1.24–1.29 (m, 20H, 10 × C**H_2_**), 1.97 (bs, 4H, 2 × NCH_2_C**H_2_**), 4.62–4.71 (m, 4H, 2 × NC**H_2_**), 8.12 (dd, 0.25H, *J* = 4 Hz, 8 Hz, **H-Ar**), 8.25 (dd, 0.75H, *J* = 4 Hz, 8 Hz, **H-Ar**), 8.28 (s, 0.25H, **H**-C=N), 8.43 (d, 0.5H, *J* = 8 Hz, **H-Ar**), 8.56 (d, 1.5H, *J* = 8 Hz, **H-Ar**), 8.65 (s, 0.75H, **H**-C=N), 8.67 (d, 0.25H, *J* = 4 Hz, **H-Ar**), 8.94 (d, 0.75H, *J* = 8 Hz, **H-Ar**), 9.10 (d, 0.25H, *J* = 4 Hz, **H-Ar**), 9.18 (d, 0.75H, *J* = 8 Hz, **H-Ar**), 9.28 (d, 0.5H, *J* = 8 Hz, **H-Ar**), 9.32 (s, 0.25H, **H-Ar**), 9.39 (d, 1.5H, *J* = 4 Hz, **H-Ar**), 9.53 (s, 0.75H, **H-Ar**), 13.01 (s, 1H, CON**H**). ^13^C NMR (DMSO-*d*_6_, 100 MHz): δ_C_ = 14.43 (2 × **C**H_3_), 22.53, 25.90, 25.97, 28.83, 28.87, 28.93, 30.97, 31.08, 31.19, 31.61 (12 ×**C**H_2_), 61.51, 61.58 (2 × N**C**H_2_), 126.83, 127.80, 128.47, 128.69, 134.22, 134.53, 140.00, 141.82, 142.47, 144.34, 144.40, 145.45, 145.67, 146.30, 147.12, 149.16 (**C**-Ar), 159.93, 166.33 (**C**=N, **C**=O). HRMS (ESI) m/z = 706.1604 [M^+^].

##### Characterization of 1-Nonyl-3-((2-(1-nonylpyridin-1-ium-4-carbonyl)hydrazono)methyl)pyridin-1-ium Iodide (**10**)

Yield: 85%. ^1^H NMR (DMSO-*d_6_*, 400 MHz): δ_H_ = 0.84 (t, 6H, *J* = 8 Hz, 2 × C**H_3_**), 1.24–1.30 (m, 24H, 12 × C**H_2_**), 1.96 (bs, 4H, 2 × NCH_2_C**H_2_**), 4.61–4.70 (m, 4H, 2 × NC**H_2_**), 8.12 (dd, 0.25H, *J* = 4 Hz, 8 Hz, **H-Ar**), 8.24 (dd, 0.75H, *J* = 4 Hz, 8 Hz, **H-Ar**), 8.28 (s, 0.25H, **H**-C=N), 8.43 (d, 0.5H, *J* = 8 Hz, **H-Ar**), 8.55 (d, 1.5H, *J* = 4 Hz, **H-Ar**), 8.64 (s, 0.75H, **H**-C=N), 8.65 (d, 0.25H, *J* = 4 Hz, **H-Ar**), 8.94 (d, 0.75H, *J* = 8 Hz, **H-Ar**), 9.07 (d, 0.25H, *J* = 8 Hz, **H-Ar**), 9.16 (d, 0.75H, *J* = 4 Hz, **H-Ar**), 9.27 (d, 0.5H, *J* = 8 Hz, **H-Ar**), 9.30 (s, 0.25H, **H-Ar**), 9.38 (d, 1.5H, *J* = 8 Hz, **H-Ar**), 9.51 (s, 0.75H, **H-Ar**), 13.01 (s, 1H, CON**H**). ^13^C NMR (DMSO-*d*_6_, 100 MHz): δ_C_ = 14.44 (2 × **C**H_3_), 22.55, 25.85, 25.89, 28.86, 28.90, 29.04, 29.22, 30.97, 31.19, 31.70 (14 × **C**H_2_), 61.54, 61.59 (2 × N**C**H_2_), 126.84, 127.82, 128.45, 128.69, 134.25, 134.55, 140.04, 141.51, 142.51, 144.31, 144.44, 145.74, 146.30, 147.15, 149.18 (**C**-Ar), 159.96, 166.34 (**C**=N, **C**=O). HRMS (ESI) m/z = 734.1917 [M^+^].

##### Characterization of 1-Decyl-3-((2-(1-decylpyridin-1-ium-4-carbonyl)hydrazono)methyl)pyridin-1-ium Iodide (**11**)

Yield: 86%. ^1^H NMR (DMSO-*d*_6_, 400 MHz): δ_H_ = 0.84 (t, 6H, *J* = 4 Hz, 2 × C**H_3_**), 1.24–1.30 (m, 28H, 14 × C**H_2_**), 1.96 (bs, 4H, 2 × NCH_2_C**H_2_**), 4.58–4.70 (m, 4H, 2 × NC**H_2_**), 8.12 (dd, 0.25H, *J* = 4 Hz, 8 Hz, **H-Ar**), 8.24 (dd, 0.75H, *J* = 4 Hz, 8 Hz, **H-Ar**), 8.27 (s, 0.25H, **H**-C=N), 8.43 (d, 0.5H, *J* = 8 Hz, **H-Ar**), 8.55 (d, 1.5H, *J* = 4 Hz, **H-Ar**), 8.63 (s, 0.75H, **H**-C=N), 8.65 (d, 0.25H, *J* = 4 Hz, **H-Ar**), 8.94 (d, 0.75H, *J* = 8 Hz, **H-Ar**), 9.08 (d, 0.25H, *J* = 8 Hz, **H-Ar**), 9.15 (d, 0.75H, *J* = 4 Hz, **H-Ar**), 9.26 (d, 0.5H, *J* = 8 Hz, **H-Ar**), 9.28 (s, 0.25H, **H-Ar**), 9.36 (d, 1.5H, *J* = 4 Hz, **H-Ar**), 9.50 (s, 0.75H, **H-Ar**), 13.00 (s, 1H, CON**H**). ^13^C NMR (DMSO-*d*_6_, 100 MHz): δ_C_ = 14.45 (2 × **C**H_3_), 22.57, 25.85, 25.90, 28.86, 28.90, 29.13, 29.27, 29.35, 31.18, 31.74 (16 × **C**H_2_), 61.51, 61.61 (2 × N**C**H_2_), 126.84, 127.83, 128.45, 128.69, 134.25, 134.55, 141.51, 142.54, 144.28, 144.45, 145.75, 146.31, 147.16, 149.19 (**C**-Ar), 159.96, 166.35 (**C**=N, **C**=O). HRMS (ESI) m/z = 762.2230 [M^+^].

##### Characterization of 1-Dodecyl-3-((2-(1-dodecylpyridin-1-ium-4-carbonyl)hydrazono)methyl)pyridin-1-ium Iodide (**12**)

Yield: 88%. ^1^H NMR (DMSO-*d*_6_, 400 MHz): δ_H_ = 0.84 (t, 6H, *J* = 4 Hz, 2 × C**H_3_**), 1.23–1.29 (m, 36H, 18 × C**H_2_**), 1.96 (bs, 4H, 2 × NCH_2_C**H_2_**), 4.58–4.71 (m, 4H, 2 × NC**H_2_**), 8.12 (dd, 0.25H, *J* = 4 Hz, 8 Hz, **H-Ar**), 8.25 (dd, 0.75H, *J* = 4 Hz, 8 Hz, **H-Ar**), 8.27 (s, 0.25H, **H**-C=N), 8.43 (d, 0.5H, *J* = 8 Hz, **H-Ar**), 8.55 (d, 1.5H, *J* = 4 Hz, **H-Ar**), 8.63 (s, 0.75H, **H**-C=N), 8.65 (d, 0.25H, *J* = 4 Hz, **H-Ar**), 8.94 (d, 0.75H, *J* = 8 Hz, **H-Ar**), 9.08 (d, 0.25H, *J* = 8 Hz, **H-Ar**), 9.16 (d, 0.75H, *J* = 8 Hz, **H-Ar**), 9.26 (d, 0.5H, *J* = 8 Hz, **H-Ar**), 9.28 (s, 0.25H, **H-Ar**), 9.37 (d, 1.5H, *J* = 8 Hz, H-Ar), 9.50 (s, 0.75H, H-Ar), 13.00 (s, 1H, CON**H**). ^13^C NMR (DMSO-*d*_6_, 100 MHz): δ_C_ = 14.45 (2 × **C**H_3_), 22.57, 25.85, 25.90, 28.87, 28.91, 29.19, 29.28, 29.40, 29.49, 31.19, 31.76 (20 × **C**H_2_), 61.56, 61.61 (2 × N**C**H_2_), 126.84, 127.84, 128.45, 128.69, 134.25, 134.56, 141.51, 142.54, 144.28, 144.44, 145.75, 145.77, 146.31, 147.17, 149.19 (**C**-Ar), 159.95, 166.35 (**C**=N, **C**=O). HRMS (ESI) m/z = 818.2856 [M^+^].

##### Characterization of 1-Tetradecyl-3-((2-(1-tetradecylpyridin-1-ium-4-carbonyl)hydrazono)methyl)pyridin-1-ium Iodide (**13**)

Yield: 87%. ^1^H NMR (DMSO-*d*_6_, 400 MHz): δ_H_ = 0.84 (t, 6H, *J* = 4 Hz, 2 × C**H_3_**), 1.23–1.29 (m, 44H, 22 × C**H_2_**), 1.96 (bs, 4H, 2 × NCH_2_C**H_2_**), 4.58–4.69 (m, 4H, 2 × NC**H_2_**), 8.12 (dd, 0.25H, *J* = 4 Hz, 8 Hz, **H**-Ar), 8.24 (dd, 0.75H, *J* = 4 Hz, 8 Hz, **H**-Ar), 8.27 (s, 0.25H, **H**-C=N), 8.43 (d, 0.5H, *J* = 8 Hz, **H**-Ar), 8.55 (d, 1.5H, *J* = 4 Hz, **H**-Ar), 8.63 (s, 0.75H, **H**-C=N), 8.65 (d, 0.25H, *J* = 4 Hz, **H**-Ar), 8.93 (d, 0.75H, *J* = 8 Hz, **H**-Ar), 9.07 (d, 0.25H, *J* = 8 Hz, **H**-Ar), 9.15 (d, 0.75H, *J* = 4 Hz, **H**-Ar), 9.25 (d, 0.5H, *J* = 4 Hz, **H**-Ar), 9.26 (s, 0.25H, **H**-Ar), 9.36 (d, 1.5H, *J* = 4 Hz, **H**-Ar), 9.50 (s, 0.75H, **H**-Ar), 12.99 (s, 0.75H, CON**H**). 13.00 (s, 0.25H, CON**H**). ^13^C NMR (DMSO-*d*_6_, 100 MHz): δ_C_ = 14.44 (2 × **C**H_3_), 22.57, 25.85, 25.91, 28.87, 28.92, 29.19, 29.29, 29.41, 29.50, 31.19, 31.77 (24 × **C**H_2_), 61.55, 61.61 (2 × N**C**H_2_), 126.84, 127.84, 128.45, 128.69, 134.25, 134.56, 141.51, 142.54, 144.28, 144.45, 145.75, 145.77, 146.31, 147.17, 149.19 (**C-**Ar), 159.92, 166.36 (**C**=N, **C**=O). HRMS (ESI) m/z = 874.3482 [M^+^].

##### Characterization of 1-Hexadecyl-3-((2-(1-hexadecylpyridin-1-ium-4-carbonyl)hydrazono)methyl)pyridin-1-ium Iodide (**14**)

Yield: 90%.^1^H NMR (DMSO-*d*_6_, 400 MHz): δ_H_ = 0.84 (t, 6H, *J* = 8 Hz, 2 × C**H_3_**), 1.23–1.29 (m, 52H, 26 × C**H_2_**), 1.96 (bs, 4H, 2 × NCH_2_C**H_2_**), 4.58–4.69 (m, 4H, 2 × NC**H_2_**), 8.13 (dd, 0.25H, *J* = 4 Hz, 8 Hz, **H**-Ar), 8.24 (dd, 0.75H, *J* = 4 Hz, 8 Hz, **H**-Ar), 8.27 (s, 0.25H, **H**-C=N), 8.43 (d, 0.5H, *J* = 8 Hz, **H**-Ar), 8.55 (d, 1.5H, *J* = 4 Hz, **H**-Ar), 8.63 (s, 0.75H, **H**-C=N), 8.65 (d, 0.25H, *J* = 4 Hz, **H**-Ar), 8.93 (d, 0.75H, *J* = 8 Hz, **H**-Ar), 9.07 (d, 0.25H, *J* = 8 Hz, **H**-Ar), 9.15 (d, 0.75H, *J* = 4 Hz, **H**-Ar), 9.25 (d, 0.5H, *J* = 4 Hz, **H**-Ar), 9.26 (s, 0.25H, **H**-Ar), 9.35 (d, 1.5H, *J* = 4 Hz, **H**-Ar), 9.48 (s, 0.75H, **H**-Ar), 12.99 (s, 1H, CON**H**). ^13^C NMR (DMSO-*d*_6_, 100 MHz): δ_C_ = 14.44 (2 × **C**H_3_), 22.57, 25.85, 25.91, 28.87, 28.92, 29.19, 29.29, 29.41, 29.50, 31.19, 31.77 (28 × **C**H_2_), 61.55, 61.61 (2 × N**C**H_2_), 126.84, 127.84, 128.45, 128.69, 134.25, 134.56, 141.51, 142.54, 144.28, 144.45, 145.75, 145.77, 146.31, 147.17, 149.19 (**C**-Ar), 159.92, 166.36 (**C**=N, **C**=O). HRMS (ESI) m/z = 930.4108 [M^+^].

##### Characterization of 1-Octadecyl-3-((2-(1-octadecylpyridin-1-ium-4-carbonyl)hydrazono)methyl)pyridin-1-ium Iodide (**15**)

Yield: 88%; mp: 192–193 °C. ^1^H NMR (DMSO-*d*_6_,, 400 MHz): δ_H_ = 0.84 (t, 6H, *J* = 8 Hz, 2 × C**H_3_**), 1.23–1.29 (m, 60H, 30 × C**H_2_**), 1.96 (bs, 4H, 2 × NCH_2_C**H_2_**), 4.59–4.69 (m, 4H, 2 × NC**H_2_**), 8.13 (dd, 0.25H, *J* = 4 Hz, 8 Hz, **H**-Ar), 8.24 (dd, 0.75H, *J* = 4 Hz, 8 Hz, **H**-Ar), 8.27 (s, 0.25H, **H**-C=N), 8.43 (d, 0.5H, *J* = 8 Hz, **H**-Ar), 8.55 (d, 1.5H, *J* = 4 Hz, **H**-Ar), 8.63 (s, 0.75H, **H**-C=N), 8.65 (d, 0.25H, *J* = 4 Hz, **H**-Ar), 8.93 (d, 0.75H, *J* = 8 Hz, **H**-Ar), 9.07 (d, 0.25H, *J* = 8 Hz, **H**-Ar), 9.15 (d, 0.75H, *J* = 4 Hz, **H**-Ar), 9.25 (d, 0.5H, *J* = 4 Hz, **H**-Ar), 9.26 (s, 0.25H, **H**-Ar), 9.35 (d, 1.5H, *J* = 4 Hz, **H**-Ar), 9.48 (s, 0.75H, **H**-Ar), 12.65 (s, 0.75H, CON**H**), 12.99 (s, 0.25H, CON**H**). ^13^C NMR (DMSO-*d*_6_, 100 MHz): δ_C_ = 14.44 (2 × **C**H_3_), 22.57, 25.85, 25.91, 28.87, 28.92, 29.19, 29.29, 29.41, 29.50, 31.19, 31.77 (32 × **C**H_2_), 61.55, 61.61 (2 × N**C**H_2_), 126.84, 127.84, 128.45, 128.69, 134.25, 134.56, 141.51, 142.54, 144.28, 144.45, 145.75, 145.77, 146.31, 147.17, 149.19 (**C**-Ar), 159.92, 166.36 (**C**=N, **C**=O). HRMS (ESI) m/z = 986.4734 [M^+^].

#### 3.1.2. General Metathesis Procedure for the Synthesis **16**–**36**

A suspension of compounds **9**–**15** (1 mmol) and the appropriate fluorinated metal salt: (2.2 mmol) in CH_3_CN (25 mL) were heated under reflux for 16 h. The targeted dicationic pyridinium lipophiles tethering fluorinated counter anions **16**–**36** were substantially brought on by the evaporation of acetonitrile.

##### Characterization of 1-Octyl-3-((2-(1-octylpyridin-1-ium-4-carbonyl)hydrazono)methyl)pyridin-1-ium Hexafluorophosphate (**16**)

Yield: 89%. ^1^H NMR (DMSO-*d*_6_, 400 MHz): δ_H_ = 0.84 (t, 6H, *J* = 8 Hz, 2 × C**H_3_**), 1.24–1.29 (m, 20H, 10 × C**H_2_**), 1.97 (bs, 4H, 2 × NCH_2_C**H_2_**), 4.62–4.71 (m, 4H, 2 × NC**H_2_**), 8.12 (dd, 0.25H, *J* = 4 Hz, 8 Hz, **H**-Ar), 8.25 (dd, 0.75H, *J* = 4 Hz, 8 Hz, **H**-Ar), 8.28 (s, 0.25H, **H**-C=N), 8.43 (d, 0.5H, *J* = 8 Hz, **H**-Ar), 8.56 (d, 1.5H, *J* = 8 Hz, **H**-Ar), 8.65 (s, 0.75H, **H**-C=N), 8.67 (d, 0.25H, *J* = 4 Hz, **H**-Ar), 8.94 (d, 0.75H, *J* = 8 Hz, **H**-Ar), 9.10 (d, 0.25H, *J* = 4 Hz, **H**-Ar), 9.18 (d, 0.75H, *J* = 8 Hz, **H**-Ar), 9.28 (d, 0.5H, *J* = 8 Hz, **H**-Ar), 9.32 (s, 0.25H, **H**-Ar), 9.39 (d, 1.5H, *J* = 4 Hz, **H**-Ar), 9.53 (s, 0.75H, **H**-Ar), 13.01 (s, 1H, CON**H**). ^13^C NMR (DMSO-*d*_6_, 100 MHz): δ_C_ = 14.43 (2 × **C**H_3_), 22.53, 25.90, 25.97, 28.83, 28.87, 28.93, 30.97, 31.08, 31.19, 31.61 (12 × **C**H_2_), 61.51, 61.58 (2 × N**C**H_2_), 126.83, 127.80, 128.47, 128.69, 134.22, 134.53, 140.00, 141.82, 142.47, 144.34, 144.40, 145.45, 145.67, 146.30, 147.12, 149.16 (**C**-Ar), 159.93, 166.33 (**C**=N, **C**=O). ^31^P NMR (DMSO-*d*_6_, 162 MHz): δ_P_ = (-153.03) to (-135.47) (m, 2P, 2 × **P**F_6_). ^19^F NMR (DMSO-*d*_6_, 377 MHz): δ_F_ = -69.18 (d, 12F, 2 × P**F_6_**). HRMS (ESI) *m/z* = 742.2799 [M^+^].

##### Characterization of 1-Octyl-3-((2-(1-octylpyridin-1-ium-4-carbonyl)hydrazono)methyl)pyridin-1-ium Tetrafluoroborate (**17**)

Yield in 91%. ^1^H NMR (DMSO-*d*_6_, 400 MHz): δ_H_ = 0.84 (t, 6H, *J* = 8 Hz, 2 × C**H_3_**), 1.24–1.29 (m, 20H, 10 × C**H_2_**), 1.97 (bs, 4H, 2 × NCH_2_C**H_2_**), 4.62–4.71 (m, 4H, 2 × NC**H_2_**), 8.12 (dd, 0.25H, *J* = 4 Hz, 8 Hz, **H**-Ar), 8.25 (dd, 0.75H, *J* = 4 Hz, 8 Hz, **H**-Ar), 8.28 (s, 0.25H, **H**-C=N), 8.43 (d, 0.5H, *J* = 8 Hz, **H**-Ar), 8.56 (d, 1.5H, *J* = 8 Hz, **H**-Ar), 8.65 (s, 0.75H, **H**-C=N), 8.67 (d, 0.25H, *J* = 4 Hz, **H**-Ar), 8.94 (d, 0.75H, *J* = 8 Hz, **H**-Ar), 9.10 (d, 0.25H, *J* = 4 Hz, **H**-Ar), 9.18 (d, 0.75H, *J* = 8 Hz, **H**-Ar), 9.28 (d, 0.5H, *J* = 8 Hz, **H**-Ar), 9.32 (s, 0.25H, **H**-Ar), 9.39 (d, 1.5H, *J* = 4 Hz, **H**-Ar), 9.53 (s, 0.75H, **H**-Ar), 13.01 (s, 1H, CON**H**). ^13^C NMR (DMSO-*d*_6_, 100 MHz): δ_C_ = 14.43 (2 × **C**H_3_), 22.53, 25.90, 25.97, 28.83, 28.87, 28.93, 30.97, 31.08, 31.19, 31.61 (12 × **C**H_2_), 61.51, 61.58 (2 × N**C**H_2_), 126.83, 127.80, 128.47, 128.69, 134.22, 134.53, 140.00, 141.82, 142.47, 144.34, 144.40, 145.45, 145.67, 146.30, 147.12, 149.16 (Ar-**C**), 159.93, 166.33 (**C**=N, **C**=O). ^11^B NMR (DMSO-*d*_6_, 128 MHz): δ_B_ = (-1.39) to (-1.23) (m, 2B, 2 × **B**F_4_). ^19^F NMR (DMSO-*d*_6_, 377 MHz): δ_F_ = −148.20, −148.14 (2d, 8F, 2 × B**F**_4_). HRMS (ESI) *m/z* = 626.3573 [M^+^].

##### Characterization of 1-Octyl-3-((2-(1-octylpyridin-1-ium-4-carbonyl)hydrazono)methyl)pyridin-1-ium Trifluoroacetate (**18**)

Yield: 95%. ^1^H NMR (DMSO-*d*_6_, 400 MHz): δ_H_ = 0.84 (t, 6H, *J* = 8 Hz, 2 × C**H_3_**), 1.24–1.29 (m, 20H, 10 × C**H_2_**), 1.97 (bs, 4H, 2 × NCH_2_C**H_2_**), 4.62–4.71 (m, 4H, 2 × NC**H_2_**), 8.12 (dd, 0.25H, *J* = 4 Hz, 8 Hz, **H**-Ar), 8.25 (dd, 0.75H, *J* = 4 Hz, 8 Hz, **H**-Ar), 8.28 (s, 0.25H, **H**-C=N), 8.43 (d, 0.5H, *J* = 8 Hz, **H**-Ar), 8.56 (d, 1.5H, *J* = 8 Hz, **H**-Ar), 8.65 (s, 0.75H, **H**-C=N), 8.67 (d, 0.25H, *J* = 4 Hz, **H**-Ar), 8.94 (d, 0.75H, *J* = 8 Hz, **H**-Ar), 9.10 (d, 0.25H, *J* = 4 Hz, **H**-Ar), 9.18 (d, 0.75H, *J* = 8 Hz, **H**-Ar), 9.28 (d, 0.5H, *J* = 8 Hz, **H**-Ar), 9.32 (s, 0.25H, **H**-Ar), 9.39 (d, 1.5H, *J* = 4 Hz, **H**-Ar), 9.53 (s, 0.75H, **H**-Ar), 13.01 (s, 1H, CON**H**). ^13^C NMR (DMSO-*d*_6_, 100 MHz): δ_C_ = 14.43 (2 × **C**H_3_), 22.53, 25.90, 25.97, 28.83, 28.87, 28.93, 30.97, 31.08, 31.19, 31.61 (12 × **C**H_2_), 61.51, 61.58 (2 × N**C**H_2_), 126.83, 127.80, 128.47, 128.69, 134.22, 134.53, 140.00, 141.82, 142.47, 144.34, 144.40, 145.45, 145.67, 146.30, 147.12, 149.16 (**C**-Ar), 159.93, 166.33 (**C**=N, **C**=O). ^19^F NMR (DMSO-*d*_6_, 377 MHz): δ_F_ = −73.63 (s, 6F, 2 × C**F_3_**). HRMS (ESI) m/z = 678.3216 [M^+^].

##### Characterization of 1-Nonyl-3-((2-(1-nonylpyridin-1-ium-4-carbonyl)hydrazono)methyl)pyridin-1-ium Hexafluorophosphate (**19**)

Yield: 89%. ^1^H NMR (DMSO-*d*_6_, 400 MHz): δ_H_ = 0.84 (t, 6H, *J* = 8 Hz, 2 × C**H_3_**), 1.24–1.30 (m, 24H, 12 × C**H_2_**), 1.96 (bs, 4H, 2 × NCH_2_C**H_2_**), 4.61–4.70 (m, 4H, 2 × NC**H_2_**), 8.12 (dd, 0.25H, *J* = 4 Hz, 8 Hz, **H**-Ar), 8.24 (dd, 0.75H, *J* = 4 Hz, 8 Hz, **H**-Ar), 8.28 (s, 0.25H, **H**-C=N), 8.43 (d, 0.5H, *J* = 8 Hz, **H**-Ar), 8.55 (d, 1.5H, *J* = 4 Hz, **H**-Ar), 8.64 (s, 0.75H, **H**-C=N), 8.65 (d, 0.25H, *J* = 4 Hz, **H**-Ar), 8.94 (d, 0.75H, *J* = 8 Hz, **H**-Ar), 9.07 (d, 0.25H, *J* = 8 Hz, **H**-Ar), 9.16 (d, 0.75H, *J* = 4 Hz, **H**-Ar), 9.27 (d, 0.5H, *J* = 8 Hz, **H**-Ar), 9.30 (s, 0.25H, **H**-Ar), 9.38 (d, 1.5H, *J* = 8 Hz, **H**-Ar), 9.51 (s, 0.75H, **H**-Ar), 13.01 (s, 1H, CON**H**). ^13^C NMR (DMSO-*d*_6_, 100 MHz): δ_C_ = 14.44 (2 × **C**H_3_), 22.55, 25.85, 25.89, 28.86, 28.90, 29.04, 29.22, 30.97, 31.19, 31.70 (14 × **C**H_2_), 61.54, 61.59 (2 × N**C**H_2_), 126.84, 127.82, 128.45, 128.69, 134.25, 134.55, 140.04, 141.51, 142.51, 144.31, 144.44, 145.74, 146.30, 147.15, 149.18 (**C**-Ar), 159.96, 166.34 (**C**=N, **C**=O). ^31^P NMR (DMSO-*d*_6_, 162 MHz): δ_P_ = (−153.19) to (−135.75) (m, 2P, 2 × **P**F_6_). ^19^F NMR (DMSO-*d*_6_, 377 MHz): δ_F_ = −69.19 (d, 12F, 2 × P**F_6_**). HRMS (ESI) *m/z* = 770.3112 [M^+^].

##### Characterization of 1-Nonyl-3-((2-(1-nonylpyridin-1-ium-4-carbonyl)hydrazono)methyl)pyridin-1-ium Tetrafluoroborate (**20**)

Yield: 96%. ^1^H NMR (DMSO-*d*_6_, 400 MHz): δ_H_ = 0.84 (t, 6H, *J* = 8 Hz, 2 × C**H_3_**), 1.24–1.30 (m, 24H, 12 × C**H_2_**), 1.96 (bs, 4H, 2 × NCH_2_C**H_2_**), 4.61–4.70 (m, 4H, 2 × NC**H_2_**), 8.12 (dd, 0.25H, *J* = 4 Hz, 8 Hz, **H**-Ar), 8.24 (dd, 0.75H, *J* = 4 Hz, 8 Hz, **H**-Ar), 8.28 (s, 0.25H, **H**-C=N), 8.43 (d, 0.5H, *J* = 8 Hz, **H**-Ar), 8.55 (d, 1.5H, *J* = 4 Hz, **H**-Ar), 8.64 (s, 0.75H, **H**-C=N), 8.65 (d, 0.25H, *J* = 4 Hz, **H**-Ar), 8.94 (d, 0.75H, *J* = 8 Hz, **H**-Ar), 9.07 (d, 0.25H, *J* = 8 Hz, **H**-Ar), 9.16 (d, 0.75H, *J* = 4 Hz, **H**-Ar), 9.27 (d, 0.5H, *J* = 8 Hz, **H**-Ar), 9.30 (s, 0.25H, **H**-Ar), 9.38 (d, 1.5H, *J* = 8 Hz, **H**-Ar), 9.51 (s, 0.75H, **H**-Ar), 13.01 (s, 1H, CON**H**). ^13^C NMR (DMSO-*d*_6_, 100 MHz): δ_C_ = 14.44 (2 × **C**H_3_), 22.55, 25.85, 25.89, 28.86, 28.90, 29.04, 29.22, 30.97, 31.19, 31.70 (14 × **C**H_2_), 61.54, 61.59 (2 × N**C**H_2_), 126.84, 127.82, 128.45, 128.69, 134.25, 134.55, 140.04, 141.51, 142.51, 144.31, 144.44, 145.74, 146.30, 147.15, 149.18 (**C**-Ar), 159.96, 166.34 (**C**=N, **C**=O). ^11^B NMR (DMSO-*d*_6_, 128 MHz): δ_B_ = (−1.42) to (−1.23) (m, 2B, 2 × **B**F_4_). ^19^F NMR (DMSO-*d*_6_, 377 MHz): δ_F_ = −148.21, −148.14 (2d, 8F, 2 × B**F**_4_). HRMS (ESI) *m/z* = 654.3886 [M^+^].

##### Characterization of 1-Nonyl-3-((2-(1-nonylpyridin-1-ium-4-carbonyl)hydrazono)methyl)pyridin-1-ium Trifluoroacetate (**21**)

Yield: 95%. ^1^H NMR (DMSO-*d*_6_, 400 MHz): δ_H_ = 0.84 (t, 6H, *J* = 8 Hz, 2 × C**H_3_**), 1.24–1.30 (m, 24H, 12 × C**H_2_**), 1.96 (bs, 4H, 2 × NCH_2_C**H_2_**), 4.61–4.70 (m, 4H, 2 × NC**H_2_**), 8.12 (dd, 0.25H, *J* = 4 Hz, 8 Hz, **H**-Ar), 8.24 (dd, 0.75H, *J* = 4 Hz, 8 Hz, **H**-Ar), 8.28 (s, 0.25H, **H**-C=N), 8.43 (d, 0.5H, *J* = 8 Hz, **H**-Ar), 8.55 (d, 1.5H, *J* = 4 Hz, **H**-Ar), 8.64 (s, 0.75H, **H**-C=N), 8.65 (d, 0.25H, *J* = 4 Hz, **H**-Ar), 8.94 (d, 0.75H, *J* = 8 Hz, **H**-Ar), 9.07 (d, 0.25H, *J* = 8 Hz, **H**-Ar), 9.16 (d, 0.75H, *J* = 4 Hz, **H**-Ar), 9.27 (d, 0.5H, *J* = 8 Hz, **H**-Ar), 9.30 (s, 0.25H, **H**-Ar), 9.38 (d, 1.5H, *J* = 8 Hz, **H**-Ar), 9.51 (s, 0.75H, **H**-Ar), 13.01 (s, 1H, CON**H**). ^13^C NMR (DMSO-*d*_6_, 100 MHz): δ_C_ = 14.44 (2 × **C**H_3_), 22.55, 25.85, 25.89, 28.86, 28.90, 29.04, 29.22, 30.97, 31.19, 31.70 (14 × **C**H_2_), 61.54, 61.59 (2 × N**C**H_2_), 126.84, 127.82, 128.45, 128.69, 134.25, 134.55, 140.04, 141.51, 142.51, 144.31, 144.44, 145.74, 146.30, 147.15, 149.18 (**C**-Ar), 159.96, 166.34 (**C**=N, **C**=O). ^19^F NMR (DMSO-*d*_6_, 377 MHz): δ_F_ = −73.59 (s, 6F, 2 × C**F_3_**). HRMS (ESI) m/z = 706.3529 [M^+^].

##### Characterization of 1-Decyl-3-((2-(1-decylpyridin-1-ium-4-carbonyl)hydrazono)methyl)pyridin-1-ium Hexafluorophosphate (**22**)

Yield: 93%. ^1^H NMR (DMSO-*d*_6_, 400 MHz): δ_H_ = 0.84 (t, 6H, *J* = 4 Hz, 2 × C**H_3_**), 1.24–1.30 (m, 28H, 14 × C**H_2_**), 1.96 (bs, 4H, 2 × NCH_2_C**H_2_**), 4.58–4.70 (m, 4H, 2 × NC**H_2_**), 8.12 (dd, 0.25H, *J* = 4 Hz, 8 Hz, **H**-Ar), 8.24 (dd, 0.75H, *J* = 4 Hz, 8 Hz, **H**-Ar), 8.27 (s, 0.25H, **H**-C=N), 8.43 (d, 0.5H, *J* = 8 Hz, **H**-Ar), 8.55 (d, 1.5H, *J* = 4 Hz, **H**-Ar), 8.63 (s, 0.75H, **H**-C=N), 8.65 (d, 0.25H, *J* = 4 Hz, **H**-Ar), 8.94 (d, 0.75H, *J* = 8 Hz, **H**-Ar), 9.08 (d, 0.25H, *J* = 8 Hz, **H**-Ar), 9.15 (d, 0.75H, *J* = 4 Hz, **H**-Ar), 9.26 (d, 0.5H, *J* = 8 Hz, **H**-Ar), 9.28 (s, 0.25H, **H**-Ar), 9.36 (d, 1.5H, *J* = 4 Hz, **H**-Ar), 9.50 (s, 0.75H, **H**-Ar), 13.00 (s, 1H, CON**H**). ^13^C NMR (DMSO-*d*_6_, 100 MHz): δ_C_ = 14.45 (2 × **C**H_3_), 22.57, 25.85, 25.90, 28.86, 28.90, 29.13, 29.27, 29.35, 31.18, 31.74 (16 × **C**H_2_), 61.51, 61.61 (2 × N**C**H_2_), 126.84, 127.83, 128.45, 128.69, 134.25, 134.55, 141.51, 142.54, 144.28, 144.45, 145.75, 146.31, 147.16, 149.19 (**C**-Ar), 159.96, 166.35 (**C**=N, **C**=O). ^31^P NMR (DMSO-*d*_6_, 162 MHz): δ_P_ = (−153.03) to (−135.47) (m, 2P, 2 × **P**F_6_). ^19^F NMR (DMSO-*d*_6_, 377 MHz): δ_F_ = −69.19 (d, 12F, 2 × P**F_6_**). HRMS (ESI) *m/z* = 798.3425 [M^+^].

##### Characterization of 1-Decyl-3-((2-(1-decylpyridin-1-ium-4-carbonyl)hydrazono)methyl)pyridin-1-ium Tetrafluoroborate (**23**)

Yield: 88%.^1^H NMR (DMSO-*d*_6_, 400 MHz): δ_H_ = 0.84 (t, 6H, *J* = 4 Hz, 2 × C**H_3_**), 1.24–1.30 (m, 28H, 14 × C**H_2_**), 1.96 (bs, 4H, 2 × NCH_2_C**H_2_**), 4.58–4.70 (m, 4H, 2 × NC**H_2_**), 8.12 (dd, 0.25H, *J* = 4 Hz, 8 Hz, **H**-Ar), 8.24 (dd, 0.75H, *J* = 4 Hz, 8 Hz, **H**-Ar), 8.27 (s, 0.25H, **H**-C=N), 8.43 (d, 0.5H, *J* = 8 Hz, **H**-Ar), 8.55 (d, 1.5H, *J* = 4 Hz, **H**-Ar), 8.63 (s, 0.75H, **H**-C=N), 8.65 (d, 0.25H, *J* = 4 Hz, **H**-Ar), 8.94 (d, 0.75H, *J* = 8 Hz, **H**-Ar), 9.08 (d, 0.25H, *J* = 8 Hz, **H**-Ar), 9.15 (d, 0.75H, *J* = 4 Hz, **H**-Ar), 9.26 (d, 0.5H, *J* = 8 Hz, **H**-Ar), 9.28 (s, 0.25H, **H**-Ar), 9.36 (d, 1.5H, *J* = 4 Hz, **H**-Ar), 9.50 (s, 0.75H, **H**-Ar), 13.00 (s, 1H, CON**H**). ^13^C NMR (DMSO-*d*_6_, 100 MHz): δ_C_ = 14.45 (2 × **C**H_3_), 22.57, 25.85, 25.90, 28.86, 28.90, 29.13, 29.27, 29.35, 31.18, 31.74 (16 × **C**H_2_), 61.51, 61.61 (2 × N**C**H_2_), 126.84, 127.83, 128.45, 128.69, 134.25, 134.55, 141.51, 142.54, 144.28, 144.45, 145.75, 146.31, 147.16, 149.19 (**C**-Ar), 159.96, 166.35 (**C**=N, **C**=O). ^11^B NMR (DMSO-*d*_6_, 128 MHz): δ_B_ = (−1.40) to (−1.24) (m, 2B, 2 × **B**F_4_). ^19^F NMR (DMSO-*d*_6_, 377 MHz): δ_F_ = −148.21, −148.14 (2d, 8F, 2 × B**F**_4_). HRMS (ESI) *m/z* = 682.4199 [M^+^].

##### Characterization of 1-Decyl-3-((2-(1-decylpyridin-1-ium-4-carbonyl)hydrazono)methyl)pyridin-1-ium Trifluoroacetate (**24**)

Yield: 89%. ^1^H NMR (DMSO-*d*_6_, 400 MHz): δ_H_ = 0.84 (t, 6H, *J* = 4 Hz, 2 × C**H_3_**), 1.24–1.30 (m, 28H, 14 × C**H_2_**), 1.96 (bs, 4H, 2 × NCH_2_C**H_2_**), 4.58–4.70 (m, 4H, 2 × NC**H_2_**), 8.12 (dd, 0.25H, *J* = 4 Hz, 8 Hz, **H**-Ar), 8.24 (dd, 0.75H, *J* = 4 Hz, 8 Hz, **H**-Ar), 8.27 (s, 0.25H, **H**-C=N), 8.43 (d, 0.5H, *J* = 8 Hz, **H**-Ar), 8.55 (d, 1.5H, *J* = 4 Hz, **H**-Ar), 8.63 (s, 0.75H, **H**-C=N), 8.65 (d, 0.25H, *J* = 4 Hz, **H**-Ar), 8.94 (d, 0.75H, *J* = 8 Hz, **H**-Ar), 9.08 (d, 0.25H, *J* = 8 Hz, **H**-Ar), 9.15 (d, 0.75H, *J* = 4 Hz, **H**-Ar), 9.26 (d, 0.5H, *J* = 8 Hz, **H**-Ar), 9.28 (s, 0.25H, **H**-Ar), 9.36 (d, 1.5H, *J* = 4 Hz, **H**-Ar), 9.50 (s, 0.75H, **H**-Ar), 13.00 (s, 1H, CON**H**). ^13^C NMR (DMSO-*d*_6_, 100 MHz): δ_C_ = 14.45 (2 × **C**H_3_), 22.57, 25.85, 25.90, 28.86, 28.90, 29.13, 29.27, 29.35, 31.18, 31.74 (16 × **C**H_2_), 61.51, 61.61 (2 × N**C**H_2_), 126.84, 127.83, 128.45, 128.69, 134.25, 134.55, 141.51, 142.54, 144.28, 144.45, 145.75, 146.31, 147.16, 149.19 (**C**-Ar), 159.96, 166.35 (**C**=N, **C**=O). ^19^F NMR (DMSO-*d*_6_, 377 MHz): δ_F_ = −73.61 (s, 6F, 2 × C**F_3_**). HRMS (ESI) m/z = 734.3842 [M^+^].

##### Characterization of 1-Dodecyl-3-((2-(1-dodecylpyridin-1-ium-4-carbonyl)hydrazono)methyl)pyridin-1-ium Hexafluorophosphate (**25**)

Yield: 90%. ^1^H NMR (DMSO-*d*_6_, 400 MHz): δ_H_ = 0.84 (t, 6H, *J* = 4 Hz, 2 × C**H_3_**), 1.23–1.29 (m, 36H, 18 × C**H_2_**), 1.96 (bs, 4H, 2 × NCH_2_C**H_2_**), 4.58–4.71 (m, 4H, 2 × NC**H_2_**), 8.12 (dd, 0.25H, *J* = 4 Hz, 8 Hz, **H**-Ar), 8.25 (dd, 0.75H, *J* = 4 Hz, 8 Hz, **H**-Ar), 8.27 (s, 0.25H, **H**-C=N), 8.43 (d, 0.5H, *J* = 8 Hz, **H**-Ar), 8.55 (d, 1.5H, *J* = 4 Hz, **H**-Ar), 8.63 (s, 0.75H, **H**-C=N), 8.65 (d, 0.25H, *J* = 4 Hz, **H**-Ar), 8.94 (d, 0.75H, *J* = 8 Hz, **H**-Ar), 9.08 (d, 0.25H, *J* = 8 Hz, **H**-Ar), 9.16 (d, 0.75H, *J* = 8 Hz, **H**-Ar), 9.26 (d, 0.5H, *J* = 8 Hz, **H**-Ar), 9.28 (s, 0.25H, **H**-Ar), 9.37 (d, 1.5H, *J* = 8 Hz, **H**-Ar), 9.50 (s, 0.75H, **H**-Ar), 13.00 (s, 1H, CON**H**). ^13^C NMR (DMSO-*d*_6_, 100 MHz): δ_C_ = 14.45 (2 × **C**H_3_), 22.57, 25.85, 25.90, 28.87, 28.91, 29.19, 29.28, 29.40, 29.49, 31.19, 31.76 (20 × **C**H_2_), 61.56, 61.61 (2 × N**C**H_2_), 126.84, 127.84, 128.45, 128.69, 134.25, 134.56, 141.51, 142.54, 144.28, 144.44, 145.75, 145.77, 146.31, 147.17, 149.19 (**C**-Ar), 159.95, 166.35 (**C**=N, **C**=O). ^31^P NMR (DMSO-*d*_6_, 162 MHz): δ_P_ = (−153.02) to (−135.45) (m, 2P, 2 × **P**F_6_). ^19^F NMR (DMSO-*d*_6_, 377 MHz): δ_F_ = −69.19 (d, 12F, 2 × P**F_6_**). HRMS (ESI) *m/z* = 854.4051 [M^+^].

##### Characterization of 11-Dodecyl-3-((2-(1-dodecylpyridin-1-ium-4-carbonyl)hydrazono)methyl)pyridin-1-ium Tetrafluoroborate (**26**)

Yield: 95%. ^1^H NMR (DMSO-*d*_6_, 400 MHz): δ_H_ = 0.84 (t, 6H, *J* = 4 Hz, 2 × C**H_3_**), 1.23–1.29 (m, 36H, 18 × C**H_2_**), 1.96 (bs, 4H, 2 × NCH_2_C**H_2_**), 4.58–4.71 (m, 4H, 2 × NC**H_2_**), 8.12 (dd, 0.25H, *J* = 4 Hz, 8 Hz, **H**-Ar), 8.25 (dd, 0.75H, *J* = 4 Hz, 8 Hz, **H**-Ar), 8.27 (s, 0.25H, **H**-C=N), 8.43 (d, 0.5H, *J* = 8 Hz, **H**-Ar), 8.55 (d, 1.5H, *J* = 4 Hz, **H**-Ar), 8.63 (s, 0.75H, **H**-C=N), 8.65 (d, 0.25H, *J* = 4 Hz, **H**-Ar), 8.94 (d, 0.75H, *J* = 8 Hz, **H**-Ar), 9.08 (d, 0.25H, *J* = 8 Hz, **H**-Ar), 9.16 (d, 0.75H, *J* = 8 Hz, **H**-Ar), 9.26 (d, 0.5H, *J* = 8 Hz, **H**-Ar), 9.28 (s, 0.25H, **H**-Ar), 9.37 (d, 1.5H, *J* = 8 Hz, **H**-Ar), 9.50 (s, 0.75H, **H**-Ar), 13.00 (s, 1H, CON**H**). ^13^C NMR (DMSO-*d*_6_, 100 MHz): δ_C_ = 14.45 (2 × **C**H_3_), 22.57, 25.85, 25.90, 28.87, 28.91, 29.19, 29.28, 29.40, 29.49, 31.19, 31.76 (20 × **C**H_2_), 61.56, 61.61 (2 × N**C**H_2_), 126.84, 127.84, 128.45, 128.69, 134.25, 134.56, 141.51, 142.54, 144.28, 144.44, 145.75, 145.77, 146.31, 147.17, 149.19 (**C**-Ar), 159.95, 166.35 (**C**=N, **C**=O). ^11^B NMR (DMSO-*d*_6_, 128 MHz): δ_B_ = (−1.41) to (−1.24) (m, 2B, 2 × **B**F_4_). ^19^F NMR (DMSO-*d*_6_, 377 MHz): δ_F_ = −148.20, −148.14 (2d, 8F, 2 × B**F**_4_). HRMS (ESI) *m/z* = 738.4825 [M^+^].

##### Characterization of 1-Dodecyl-3-((2-(1-dodecylpyridin-1-ium-4-carbonyl)hydrazono)methyl)pyridin-1-ium Trifluoroacetate (**27**)

Yield: 91%. ^1^H NMR (DMSO-*d*_6_, 400 MHz): δ_H_ = 0.84 (t, 6H, *J* = 4 Hz, 2 × C**H_3_**), 1.23–1.29 (m, 36H, 18 × C**H_2_**), 1.96 (bs, 4H, 2 × NCH_2_C**H_2_**), 4.58–4.71 (m, 4H, 2 × NC**H_2_**), 8.12 (dd, 0.25H, *J* = 4 Hz, 8 Hz, **H**-Ar), 8.25 (dd, 0.75H, *J* = 4 Hz, 8 Hz, **H**-Ar), 8.27 (s, 0.25H, **H**-C=N), 8.43 (d, 0.5H, *J* = 8 Hz, **H**-Ar), 8.55 (d, 1.5H, *J* = 4 Hz, **H**-Ar), 8.63 (s, 0.75H, **H**-C=N), 8.65 (d, 0.25H, *J* = 4 Hz, **H**-Ar), 8.94 (d, 0.75H, *J* = 8 Hz, **H**-Ar), 9.08 (d, 0.25H, *J* = 8 Hz, **H**-Ar), 9.16 (d, 0.75H, *J* = 8 Hz, **H**-Ar), 9.26 (d, 0.5H, *J* = 8 Hz, **H**-Ar), 9.28 (s, 0.25H, **H**-Ar), 9.37 (d, 1.5H, *J* = 8 Hz, **H**-Ar), 9.50 (s, 0.75H, **H**-Ar), 13.00 (s, 1H, CON**H**). ^13^C NMR (DMSO-*d*_6_, 100 MHz): δ_C_ = 14.45 (2 × **C**H_3_), 22.57, 25.85, 25.90, 28.87, 28.91, 29.19, 29.28, 29.40, 29.49, 31.19, 31.76 (20 × **C**H_2_), 61.56, 61.61 (2 × N**C**H_2_), 126.84, 127.84, 128.45, 128.69, 134.25, 134.56, 141.51, 142.54, 144.28, 144.44, 145.75, 145.77, 146.31, 147.17, 149.19 (**C**-Ar), 159.95, 166.35 (**C**=N, **C**=O). ^19^F NMR (DMSO-*d*_6_, 377 MHz): δ_F_ = −73.65 (s, 6F, 2 × C**F_3_**). HRMS (ESI) m/z = 790.4468 [M^+^].

##### Characterization of 1-Tetradecyl-3-((2-(1-tetradecylpyridin-1-ium-4-carbonyl)hydrazono)methyl)pyridin-1-ium Hexafluorophosphate (**28**)

Yield: 92%. ^1^H NMR (DMSO-*d*_6_, 400 MHz): δ_H_ = 0.84 (t, 6H, *J* = 4 Hz, 2 × C**H_3_**), 1.23–1.29 (m, 44H, 22 × C**H_2_**), 1.96 (bs, 4H, 2 × NCH_2_C**H_2_**), 4.58–4.69 (m, 4H, 2 × NC**H_2_**), 8.12 (dd, 0.25H, *J* = 4 Hz, 8 Hz, **H**-Ar), 8.24 (dd, 0.75H, *J* = 4 Hz, 8 Hz, **H**-Ar), 8.27 (s, 0.25H, **H**-C=N), 8.43 (d, 0.5H, *J* = 8 Hz, **H**-Ar), 8.55 (d, 1.5H, *J* = 4 Hz, **H**-Ar), 8.63 (s, 0.75H, **H**-C=N), 8.65 (d, 0.25H, *J* = 4 Hz, **H**-Ar), 8.93 (d, 0.75H, *J* = 8 Hz, **H**-Ar), 9.07 (d, 0.25H, *J* = 8 Hz, **H**-Ar), 9.15 (d, 0.75H, *J* = 4 Hz, **H**-Ar), 9.25 (d, 0.5H, *J* = 4 Hz, **H**-Ar), 9.26 (s, 0.25H, **H**-Ar), 9.36 (d, 1.5H, *J* = 4 Hz, **H**-Ar), 9.50 (s, 0.75H, **H**-Ar), 12.99 (s, 0.75H, CON**H**). 13.00 (s, 0.25H, CON**H**). ^13^C NMR (DMSO-*d*_6_, 100 MHz): δ_C_ = 14.44 (2 × **C**H_3_), 22.57, 25.85, 25.91, 28.87, 28.92, 29.19, 29.29, 29.41, 29.50, 31.19, 31.77 (24 × **C**H_2_), 61.55, 61.61 (2 × N**C**H_2_), 126.84, 127.84, 128.45, 128.69, 134.25, 134.56, 141.51, 142.54, 144.28, 144.45, 145.75, 145.77, 146.31, 147.17, 149.19 (**C**-Ar), 159.92, 166.36 (**C**=N, **C**=O). ^31^P NMR (DMSO-*d*_6_, 162 MHz): δ_P_ = (−153.31) to (−135.84) (m, 2P, 2 × **P**F_6_). ^19^F NMR (DMSO-*d*_6_, 377 MHz): δ_F_ = −69.19 (d, 12F, 2 × P**F_6_**). HRMS (ESI) *m/z* = 910.4677 [M^+^].

##### Characterization of 1-Tetradecyl-3-((2-(1-tetradecylpyridin-1-ium-4-carbonyl)hydrazono)methyl)pyridin-1-ium Tetrafluoroborate (**29**)

Yield: 92%. ^1^H NMR (DMSO-*d*_6_, 400 MHz): δ_H_ = 0.84 (t, 6H, *J* = 4 Hz, 2 × C**H_3_**), 1.23–1.29 (m, 44H, 22 × C**H_2_**), 1.96 (bs, 4H, 2 × NCH_2_C**H_2_**), 4.58–4.69 (m, 4H, 2 × NC**H_2_**), 8.12 (dd, 0.25H, *J* = 4 Hz, 8 Hz, **H**-Ar), 8.24 (dd, 0.75H, *J* = 4 Hz, 8 Hz, **H**-Ar), 8.27 (s, 0.25H, **H**-C=N), 8.43 (d, 0.5H, *J* = 8 Hz, **H**-Ar), 8.55 (d, 1.5H, *J* = 4 Hz, **H**-Ar), 8.63 (s, 0.75H, **H**-C=N), 8.65 (d, 0.25H, *J* = 4 Hz, **H**-Ar), 8.93 (d, 0.75H, *J* = 8 Hz, **H**-Ar), 9.07 (d, 0.25H, *J* = 8 Hz, **H**-Ar), 9.15 (d, 0.75H, *J* = 4 Hz, **H**-Ar), 9.25 (d, 0.5H, *J* = 4 Hz, **H**-Ar), 9.26 (s, 0.25H, **H**-Ar), 9.36 (d, 1.5H, *J* = 4 Hz, **H**-Ar), 9.50 (s, 0.75H, **H**-Ar), 12.99 (s, 0.75H, CON**H**). 13.00 (s, 0.25H, CON**H**). ^13^C NMR (DMSO-*d*_6_, 100 MHz): δ_C_ = 14.44 (2 × **C**H_3_), 22.57, 25.85, 25.91, 28.87, 28.92, 29.19, 29.29, 29.41, 29.50, 31.19, 31.77 (24 × **C**H_2_), 61.55, 61.61 (2 × N**C**H_2_), 126.84, 127.84, 128.45, 128.69, 134.25, 134.56, 141.51, 142.54, 144.28, 144.45, 145.75, 145.77, 146.31, 147.17, 149.19 (**C**-Ar), 159.92, 166.36 (**C**=N, **C**=O). ^11^B NMR (DMSO-*d*_6_, 128 MHz): δ_B_ = (−1.39) to (−1.24) (m, 2B, 2 × **B**F_4_). ^19^F NMR (DMSO-*d*_6_, 377 MHz): δ_F_ = −148.21, −148.15 (2d, 8F, 2 × B**F**_4_). HRMS (ESI) *m/z* = 794.5451 [M^+^].

##### Characterization of 1-Tetradecyl-3-((2-(1-tetradecylpyridin-1-ium-4-carbonyl)hydrazono)methyl)pyridin-1-ium Trifluoroacetate (**30**)

Yield: 89%. ^1^H NMR (DMSO-*d*_6_, 400 MHz): δ_H_ = 0.84 (t, 6H, *J* = 4 Hz, 2 × C**H_3_**), 1.23–1.29 (m, 44H, 22 × C**H_2_**), 1.96 (bs, 4H, 2 × NCH_2_C**H_2_**), 4.58–4.69 (m, 4H, 2 × NC**H_2_**), 8.12 (dd, 0.25H, *J* = 4 Hz, 8 Hz, **H**-Ar), 8.24 (dd, 0.75H, *J* = 4 Hz, 8 Hz, **H**-Ar), 8.27 (s, 0.25H, **H**-C=N), 8.43 (d, 0.5H, *J* = 8 Hz, **H**-Ar), 8.55 (d, 1.5H, *J* = 4 Hz, **H**-Ar), 8.63 (s, 0.75H, **H**-C=N), 8.65 (d, 0.25H, *J* = 4 Hz, **H**-Ar), 8.93 (d, 0.75H, *J* = 8 Hz, **H**-Ar), 9.07 (d, 0.25H, *J* = 8 Hz, **H**-Ar), 9.15 (d, 0.75H, *J* = 4 Hz, **H**-Ar), 9.25 (d, 0.5H, *J* = 4 Hz, **H**-Ar), 9.26 (s, 0.25H, **H**-Ar), 9.36 (d, 1.5H, *J* = 4 Hz, **H**-Ar), 9.50 (s, 0.75H, **H**-Ar), 12.99 (s, 0.75H, CON**H**). 13.00 (s, 0.25H, CON**H**). ^13^C NMR (DMSO-*d*_6_, 100 MHz): δ_C_ = 14.44 (2 × **C**H_3_), 22.57, 25.85, 25.91, 28.87, 28.92, 29.19, 29.29, 29.41, 29.50, 31.19, 31.77 (24 × **C**H_2_), 61.55, 61.61 (2 × N**C**H_2_), 126.84, 127.84, 128.45, 128.69, 134.25, 134.56, 141.51, 142.54, 144.28, 144.45, 145.75, 145.77, 146.31, 147.17, 149.19 (**C**-Ar), 159.92, 166.36 (**C**=N, **C**=O). ^19^F NMR (DMSO-*d*_6_, 377 MHz): δ_F_ = −73.59 (s, 6F, 2 × C**F_3_**). HRMS (ESI) m/z = 846.5094 [M^+^].

##### Characterization of 1-Hexadecyl-3-((2-(1-hexadecylpyridin-1-ium-4-carbonyl)hydrazono)methyl)pyridin-1-ium Hexafluorophosphate (**31**)

Yield: 90%. ^1^H NMR (DMSO-*d*_6_, 400 MHz): δ_H_ = 0.84 (t, 6H, *J* = 8 Hz, 2 × C**H_3_**), 1.23–1.29 (m, 52H, 26 × C**H_2_**), 1.96 (bs, 4H, 2 × NCH_2_C**H_2_**), 4.58–4.69 (m, 4H, 2 × NC**H_2_**), 8.13 (dd, 0.25H, *J* = 4 Hz, 8 Hz, **H**-Ar), 8.24 (dd, 0.75H, *J* = 4 Hz, 8 Hz, **H**-Ar), 8.27 (s, 0.25H, **H**-C=N), 8.43 (d, 0.5H, *J* = 8 Hz, **H**-Ar), 8.55 (d, 1.5H, *J* = 4 Hz, **H**-Ar), 8.63 (s, 0.75H, **H**-C=N), 8.65 (d, 0.25H, *J* = 4 Hz, **H**-Ar), 8.93 (d, 0.75H, *J* = 8 Hz, **H**-Ar), 9.07 (d, 0.25H, *J* = 8 Hz, **H**-Ar), 9.15 (d, 0.75H, *J* = 4 Hz, **H**-Ar), 9.25 (d, 0.5H, *J* = 4 Hz, **H**-Ar), 9.26 (s, 0.25H, **H**-Ar), 9.35 (d, 1.5H, *J* = 4 Hz, **H**-Ar), 9.48 (s, 0.75H, **H**-Ar), 12.99 (s, 1H, CON**H**). ^13^C NMR (DMSO-*d*_6_, 100 MHz): δ_C_ = 14.44 (2 × **C**H_3_), 22.57, 25.85, 25.91, 28.87, 28.92, 29.19, 29.29, 29.41, 29.50, 31.19, 31.77 (28 × **C**H_2_), 61.55, 61.61 (2 × N**C**H_2_), 126.84, 127.84, 128.45, 128.69, 134.25, 134.56, 141.51, 142.54, 144.28, 144.45, 145.75, 145.77, 146.31, 147.17, 149.19 (**C**-Ar), 159.92, 166.36 (**C**=N, **C**=O). ^31^P NMR (DMSO-*d*_6_, 162 MHz): δ_P_ = (−153.29) to (−135.70) (m, 2P, 2 × **P**F_6_). ^19^F NMR (DMSO-*d*_6_, 377 MHz): δ_F_ = −69.19 (d, 12F, 2 × P**F_6_**). HRMS (ESI) m/z = 966.5303 [M^+^].

##### Characterization of 1-Hexadecyl-3-((2-(1-hexadecylpyridin-1-ium-4-carbonyl)hydrazono)methyl)pyridin-1-ium Tetrafluoroborate (**32**)

Yield: 92%. ^1^H NMR (DMSO-*d*_6_, 400 MHz): δ_H_ = 0.84 (t, 6H, *J* = 8 Hz, 2 × C**H_3_**), 1.23–1.29 (m, 52H, 26 × C**H_2_**), 1.96 (bs, 4H, 2 × NCH_2_C**H_2_**), 4.58–4.69 (m, 4H, 2 × NC**H_2_**), 8.13 (dd, 0.25H, *J* = 4 Hz, 8 Hz, **H**-Ar), 8.24 (dd, 0.75H, *J* = 4 Hz, 8 Hz, **H**-Ar), 8.27 (s, 0.25H, **H**-C=N), 8.43 (d, 0.5H, *J* = 8 Hz, **H**-Ar), 8.55 (d, 1.5H, *J* = 4 Hz, **H**-Ar), 8.63 (s, 0.75H, **H**-C=N), 8.65 (d, 0.25H, *J* = 4 Hz, **H**-Ar), 8.93 (d, 0.75H, *J* = 8 Hz, **H**-Ar), 9.07 (d, 0.25H, *J* = 8 Hz, **H**-Ar), 9.15 (d, 0.75H, *J* = 4 Hz, **H**-Ar), 9.25 (d, 0.5H, *J* = 4 Hz, **H**-Ar), 9.26 (s, 0.25H, **H**-Ar), 9.35 (d, 1.5H, *J* = 4 Hz, **H**-Ar), 9.48 (s, 0.75H, **H**-Ar), 12.99 (s, 1H, CON**H**). ^13^C NMR (DMSO-*d*_6_, 100 MHz): δ_C_ = 14.44 (2 × **C**H_3_), 22.57, 25.85, 25.91, 28.87, 28.92, 29.19, 29.29, 29.41, 29.50, 31.19, 31.77 (28 × **C**H_2_), 61.55, 61.61 (2 × N**C**H_2_), 126.84, 127.84, 128.45, 128.69, 134.25, 134.56, 141.51, 142.54, 144.28, 144.45, 145.75, 145.77, 146.31, 147.17, 149.19 (**C**-Ar), 159.92, 166.36 (**C**=N, **C**=O). ^11^B NMR (DMSO-*d*_6_, 128 MHz): δ_B_ = (−1.39) to (−1.24) (m, 2B, 2 × **B**F_4_). ^19^F NMR (DMSO-*d*_6_, 377 MHz): δ_F_ = −148.14, −148.21 (2d, 8F, 2 × B**F**_4_). HRMS (ESI) *m/z* = 850.6077 [M^+^].

##### Characterization of 1-Hexadecyl-3-((2-(1-hexadecylpyridin-1-ium-4-carbonyl)hydrazono)methyl)pyridin-1-ium Trifluoroacetate (**33**)

Yield: 94%. ^1^H NMR (DMSO-*d*_6_, 400 MHz): δ_H_ = 0.84 (t, 6H, *J* = 8 Hz, 2 × C**H_3_**), 1.23–1.29 (m, 52H, 26 × C**H_2_**), 1.96 (bs, 4H, 2 × NCH_2_C**H_2_**), 4.58–4.69 (m, 4H, 2 × NC**H_2_**), 8.13 (dd, 0.25H, *J* = 4 Hz, 8 Hz, **H**-Ar), 8.24 (dd, 0.75H, *J* = 4 Hz, 8 Hz, **H**-Ar), 8.27 (s, 0.25H, **H**-C=N), 8.43 (d, 0.5H, *J* = 8 Hz, **H**-Ar), 8.55 (d, 1.5H, *J* = 4 Hz, **H**-Ar), 8.63 (s, 0.75H, **H**-C=N), 8.65 (d, 0.25H, *J* = 4 Hz, **H**-Ar), 8.93 (d, 0.75H, *J* = 8 Hz, **H**-Ar), 9.07 (d, 0.25H, *J* = 8 Hz, **H**-Ar), 9.15 (d, 0.75H, *J* = 4 Hz, **H**-Ar), 9.25 (d, 0.5H, *J* = 4 Hz, **H**-Ar), 9.26 (s, 0.25H, **H**-Ar), 9.35 (d, 1.5H, *J* = 4 Hz, **H**-Ar), 9.48 (s, 0.75H, **H**-Ar), 12.99 (s, 1H, CON**H**). ^13^C NMR (DMSO-*d*_6_, 100 MHz): δ_C_ = 14.44 (2 × **C**H_3_), 22.57, 25.85, 25.91, 28.87, 28.92, 29.19, 29.29, 29.41, 29.50, 31.19, 31.77 (28 × **C**H_2_), 61.55, 61.61 (2 × N**C**H_2_), 126.84, 127.84, 128.45, 128.69, 134.25, 134.56, 141.51, 142.54, 144.28, 144.45, 145.75, 145.77, 146.31, 147.17, 149.19 (**C**-Ar), 159.92, 166.36 (**C**=N, **C**=O). ^19^F NMR (DMSO-*d*_6_, 377 MHz): δ_F_ = −73.57 (s, 6F, 2 × C**F_3_**). HRMS (ESI) m/z = 902.5720 [M^+^].

##### Characterization of 1-Octadecyl-3-((2-(1-octadecylpyridin-1-ium-4-carbonyl)hydrazono)methyl)pyridin-1-ium Hexafluorophosphate (**34**)

Yield: 92%. ^1^H NMR (DMSO-*d*_6_, 400 MHz): δ_H_ = 0.84 (t, 6H, *J* = 8 Hz, 2 × C**H_3_**), 1.23–1.29 (m, 60H, 30 × C**H_2_**), 1.96 (bs, 4H, 2 × NCH_2_C**H_2_**), 4.59–4.69 (m, 4H, 2 × NC**H_2_**), 8.13 (dd, 0.25H, *J* = 4 Hz, 8 Hz, **H**-Ar), 8.24 (dd, 0.75H, *J* = 4 Hz, 8 Hz, **H**-Ar), 8.27 (s, 0.25H, **H**-C=N), 8.43 (d, 0.5H, *J* = 8 Hz, **H**-Ar), 8.55 (d, 1.5H, *J* = 4 Hz, **H**-Ar), 8.63 (s, 0.75H, **H**-C=N), 8.65 (d, 0.25H, *J* = 4 Hz, **H**-Ar), 8.93 (d, 0.75H, *J* = 8 Hz, **H**-Ar), 9.07 (d, 0.25H, *J* = 8 Hz, **H**-Ar), 9.15 (d, 0.75H, *J* = 4 Hz, **H**-Ar), 9.25 (d, 0.5H, *J* = 4 Hz, **H**-Ar), 9.26 (s, 0.25H, **H**-Ar), 9.35 (d, 1.5H, *J* = 4 Hz, **H**-Ar), 9.48 (s, 0.75H, **H**-Ar), 12.65 (s, 0.75H, CON**H**), 12.99 (s, 0.25H, CON**H**). ^13^C NMR (DMSO-*d*_6_, 100 MHz): δ_C_ = 14.44 (2 × **C**H_3_), 22.57, 25.85, 25.91, 28.87, 28.92, 29.19, 29.29, 29.41, 29.50, 31.19, 31.77 (32 × **C**H_2_), 61.55, 61.61 (2 × N**C**H_2_), 126.84, 127.84, 128.45, 128.69, 134.25, 134.56, 141.51, 142.54, 144.28, 144.45, 145.75, 145.77, 146.31, 147.17, 149.19 (**C**-Ar), 159.92, 166.36 (**C**=N, **C**=O). ^31^P NMR (DMSO-*d*_6_, 162 MHz): δ_P_ = (−153.29) to (−135.70) (m, 2P, 2 × **P**F_6_). ^19^F NMR (DMSO-*d*_6_, 377 MHz): δ_F_ = −69.18 (d, 12F, 2 × P**F_6_**). HRMS (ESI) m/z = 1022.5929 [M^+^].

##### Characterization of 1-Octadecyl-3-((2-(1-octadecylpyridin-1-ium-4-carbonyl)hydrazono)methyl)pyridin-1-ium Tetrafluoroborate (**35**)

Yield: 90%. ^1^H NMR (DMSO-*d*_6_, 400 MHz): δ_H_ = 0.84 (t, 6H, *J* = 8 Hz, 2 × C**H_3_**), 1.23–1.29 (m, 60H, 30 × C**H_2_**), 1.96 (bs, 4H, 2 × NCH_2_C**H_2_**), 4.59–4.69 (m, 4H, 2 × NC**H_2_**), 8.13 (dd, 0.25H, *J* = 4 Hz, 8 Hz, **H**-Ar), 8.24 (dd, 0.75H, *J* = 4 Hz, 8 Hz, **H**-Ar), 8.27 (s, 0.25H, **H**-C=N), 8.43 (d, 0.5H, *J* = 8 Hz, **H**-Ar), 8.55 (d, 1.5H, *J* = 4 Hz, **H**-Ar), 8.63 (s, 0.75H, **H**-C=N), 8.65 (d, 0.25H, *J* = 4 Hz, **H**-Ar), 8.93 (d, 0.75H, *J* = 8 Hz, **H**-Ar), 9.07 (d, 0.25H, *J* = 8 Hz, **H**-Ar), 9.15 (d, 0.75H, *J* = 4 Hz, **H**-Ar), 9.25 (d, 0.5H, *J* = 4 Hz, **H**-Ar), 9.26 (s, 0.25H, **H**-Ar), 9.35 (d, 1.5H, *J* = 4 Hz, **H**-Ar), 9.48 (s, 0.75H, **H**-Ar), 12.65 (s, 0.75H, CON**H**), 12.99 (s, 0.25H, CON**H**). ^13^C NMR (DMSO-*d*_6_, 100 MHz): δ_C_ = 14.44 (2 × **C**H_3_), 22.57, 25.85, 25.91, 28.87, 28.92, 29.19, 29.29, 29.41, 29.50, 31.19, 31.77 (32 × **C**H_2_), 61.55, 61.61 (2 × N**C**H_2_), 126.84, 127.84, 128.45, 128.69, 134.25, 134.56, 141.51, 142.54, 144.28, 144.45, 145.75, 145.77, 146.31, 147.17, 149.19 (**C**-Ar), 159.92, 166.36 (**C**=N, **C**=O). ^11^B NMR (DMSO-*d*_6_, 128 MHz): δ_B_ = (−1.24) to (−1.42) (m, 2B, 2 × **B**F_4_). ^19^F NMR (DMSO-*d*_6_, 377 MHz): δ_F_ = −148.13, −148.22 (2d, 8F, 2 × B**F**_4_). HRMS (ESI) *m/z* = 906.6703 [M^+^].

##### Characterization of 1-Octadecyl-3-((2-(1-octadecylpyridin-1-ium-4-carbonyl)hydrazono)methyl)pyridin-1-ium Trifluoroacetate (**36**)

Yield: 94%. ^1^H NMR (DMSO-*d*_6_, 400 MHz): δ_H_ = 0.84 (t, 6H, *J* = 8 Hz, 2 × C**H_3_**), 1.23–1.29 (m, 60H, 30 × C**H_2_**), 1.96 (bs, 4H, 2 × NCH_2_C**H_2_**), 4.59–4.69 (m, 4H, 2 × NC**H_2_**), 8.13 (dd, 0.25H, *J* = 4 Hz, 8 Hz, **H**-Ar), 8.24 (dd, 0.75H, *J* = 4 Hz, 8 Hz, **H**-Ar), 8.27 (s, 0.25H, **H**-C=N), 8.43 (d, 0.5H, *J* = 8 Hz, **H**-Ar), 8.55 (d, 1.5H, *J* = 4 Hz, **H**-Ar), 8.63 (s, 0.75H, **H**-C=N), 8.65 (d, 0.25H, *J* = 4 Hz, **H**-Ar), 8.93 (d, 0.75H, *J* = 8 Hz, **H**-Ar), 9.07 (d, 0.25H, *J* = 8 Hz, **H**-Ar), 9.15 (d, 0.75H, *J* = 4 Hz, **H**-Ar), 9.25 (d, 0.5H, *J* = 4 Hz, **H**-Ar), 9.26 (s, 0.25H, **H**-Ar), 9.35 (d, 1.5H, *J* = 4 Hz, **H**-Ar), 9.48 (s, 0.75H, **H**-Ar), 12.65 (s, 0.75H, CON**H**), 12.99 (s, 0.25H, CON**H**). ^13^C NMR (DMSO-*d*_6_, 100 MHz): δ_C_ = 14.44 (2 × **C**H_3_), 22.57, 25.85, 25.91, 28.87, 28.92, 29.19, 29.29, 29.41, 29.50, 31.19, 31.77 (32 × **C**H_2_), 61.55, 61.61 (2 × N**C**H_2_), 126.84, 127.84, 128.45, 128.69, 134.25, 134.56, 141.51, 142.54, 144.28, 144.45, 145.75, 145.77, 146.31, 147.17, 149.19 (**C**-Ar), 159.92, 166.36 (**C**=N, **C**=O). ^19^F NMR (DMSO-*d*_6_, 377 MHz): δ_F_ = −73.50 (s, 6F, 2 × C**F_3_**). HRMS (ESI) m/z = 958.6346 [M^+^].

### 3.2. Biological Assay

#### 3.2.1. Cancer Cell Viability

##### Cell Lines and Culture Conditions

The human lung cancer cell lines A549, H1299 and H661 and the normal cells dermal fibroblasts were obtained from the American Type Culture Collection ATCC. Cells were cultured as a monolayer at seeding density of 8 × 10^3^ cells per well in high glucose Dulbecco’s modified eagle medium (DMEM) (Invitrogen, USA), as previously described [31]. The in vitro assessment of the antitumorigenic activities related to the studied compounds was undertaken using the 3-(4,5-dimethylthiazol-2yl)-2,5-diphenyl tetrazolium bromide (MTT) colorimetric assay, as previously reported [32]. The compounds under investigation were tested in three triplicates of each concentration in three independent assays for a total of nine replicates. Both positive (cisplatin) and negative (untreated culture) controls were applied under the same experimental conditions. Cells were cultured for 48 h at 37 °C in a 5% CO_2_ incubator. Afterwards, MTT assay was carried out where viable cell count was determined based on the amount of formazan crystals, as verified by the absorbance at 490 nm.

##### Statistical Analysis

Data was analyzed by Graphpad Prism software (Graphpad Software Inc. Jolla, CA, USA) for the determination of IC_50_ value. All data were expressed as the average of triplicate experiments.

#### 3.2.2. Wound Healing Assay

A549 cells were seeded in inserts (Ibidi GmbH, Gräfelfing, Germany) at concentration of 30,000 per insert in 70 μL medium and incubated for 24 h. Afterward, inserts were removed and cells incubated with 10 μg/mL of mitomycin C for 2 h to stop cell proliferation [8]. The medium was removed, and cells were washed three times then cells were treated with 10 µM concentration of the examined compound. Images were captured at 24 h using BOECO microscope coupled with 5.0 Mega Cmos camera at 4× magnification. Digital images were taken using ISCapture software and wound width was measured using MIPAR software (MIPAR, Worthington, OH, USA).

#### 3.2.3. Colony Formation in Soft Agar Assay

To carry out the soft agar assay, a bottom agar film of 1% (*w*/*v*) was prepared by mixing autoclaved 1% (*w*/*v*) agar solution with 2× DMEM medium in a 1:1 ratio and allowed to solidify at room temperature [33]. A mixture of 1 × 10^4^ of A549 cells treated with either compound NV7 (5 µM and 10 µM) or NV8 (5 µM and 10 µM) and 0.6% (*w*/*v*) agar solution was prepared in 1:1 ratio and poured on top of the bottom layer and left to solidify. Plate was incubated for 2 weeks. Photos were captured after 12 days using EVOS XL Core imaging system (Invitrogen, Waltham, MA, USA).

## 4. Conclusions

Ionic liquids are becoming increasingly important as anticancer candidates. By quaternizing the dipyridinium hydrazone with different long alkyl halides, some novel dipyridinium iodide-based ionic liquids harboring different alkyl side chains were successfully synthesized and subjected to a metathetical anion exchange to make the targeted task DiILs tether the fluorinated counter anion. Their structures were elucidated based on several NMR experiments. The synthesized compounds were evaluated for their antitumorigenic activities, and the results indicated that DILs tethering an alkyl chain of 10 to 16 carbons had significant anticancer activities against the examined lung cancer cell lines. Moreover, some compounds were further investigated for their ability to suppress metastasis and the colony-formation ability of the cancer cells and the results revealed considerable inhibition for both cancer promoting events, suggesting therapeutic potential for the reported compounds in lung cancer treatment.

## Figures and Tables

**Table 1:**
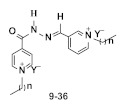
List of the dicationic pyridinium lipophile-based ionic liquids incorporating hydrazone anion **9**–**36**.

Compound No.	n	Y	Mp (°C)
** 9 **	7	I	82–84
** 10 **	8	I	76–78
** 11 **	9	I	70–72
** 12 **	11	I	63–65
** 13 **	13	I	Syrup
** 14 **	15	I	Syrup
** 15 **	17	I	Syrup
** 16 **	7	PF_6_	58–59
** 17 **	7	BF_4_	Syrup
** 18 **	7	CF_3_COO	Syrup
** 19 **	8	PF_6_	49–50
** 20 **	8	BF_4_	Syrup
** 21 **	8	CF_3_COO	Syrup
** 22 **	9	PF_6_	42–44
** 23 **	9	BF_4_	Syrup
** 24 **	9	CF_3_COO	Syrup
** 25 **	11	PF_6_	37–38
** 26 **	11	BF_4_	Syrup
** 27 **	11	CF_3_COO	Syrup
** 28 **	13	PF_6_	Syrup
** 29 **	13	BF_4_	Syrup
** 30 **	13	CF_3_COO	Syrup
** 31 **	15	PF_6_	Syrup
** 32 **	15	BF_4_	Syrup
** 33 **	15	CF_3_COO	Syrup
** 34 **	17	PF_6_	Syrup
** 35 **	17	BF_4_	Syrup
** 36 **	17	CF_3_COO	Syrup

**Table 2:** IC_50_ (µM) values of the examined compounds against three different human lung cancer cell lines and the human dermal fibroblasts for 48 h. Data are expressed as mean of three independent experiments (N = 9). SD did not exceed 5%.

Compound No.	A549 (µM)	H1299 (µM)	H661 (µM)	Fibroblasts (µM)
**9**	75.3	64.27	74.20	>300
**10**	55.56	63.11	60.14	>300
**11**	15.13	19.02	23.51	>300
**12**	26.94	30.08	29.82	>300
**13**	36.83	38.04	38.19	>300
**14**	7.111	10.09	11.23	>300
**15**	26.24	22.08	26.13	>300
**16**	45.34	42.72	48.10	>300
**17**	69.08	66.0	61.24	>300
**18**	78.54	71.04	74.50	>300
**19**	23.81	20.19	19.88	>300
**20**	31.42	32.62	33.02	>300
**21**	33.48	28.03	19.41	>300
**22**	23.47	29.12	25.60	>300
**23**	13.51	10.76	11.3	>300
**24**	20.01	19.00	18.05	>300
**25**	40.03	33.81	38.00	>300
**26**	44.03	39.34	40.36	>300
**27**	14.33	12.05	10.50	>300
**28**	35.71	29.99	39.17	>300
**29**	42.05	44.63	46.82	>300
**30**	38.64	38.52	41.90	>300
**31**	82.22	93.06	96.21	>300
**32**	114	108.19	103.72	>300
**33**	87.75	92.30	100.1	>300
**34**	146	124.78	118.04	>300
**35**	421.2	399.10	382.5	>1000
**36**	920	947	904	>1000
**Cisplatin**	23	18	32	>100

**Table 3:** Effect of treatment on **A549** cells migration.

Compound No.	Wound Closure %	*p*-Value
Control	100%	
**22** (10 µm)	67.64%	0.0001 significant
**23** (10 µm)	67.12%	0.0001 significant

**Table 4:** Effect of treatment on A549 anchorage-independent cell growth.

Compound No.	Average Colony Size	*p* Value	Average Colony Count	*p* Value
(10 µM) **22**	11159	<0.0001 significant	98	<0.0001 significant
(5 µM) **22**	16356	<0.0001 significant	210	<0. 001 significant
(10 µM) **23**	16837	<0.0001 significant	45	<0.0001 significant
(5 µM) **23**	43771	<0.0001 significant	135	<0.0001 significant

## Data Availability

Not applicable.

## Supplementary Materials

The following are available online at https://www.mdpi.com/article/10.3390/ijms221910487/s1, Figure S1: title, Table S1: title, Video S1: title.

## Abbreviations

DiILs	Di-ionic liquids
NMR	Nuclear magnetic resonance
LC	Lung cancer
SCLC	Small cell lung cancer
NSCLC	Non-small cell lung cancer
APIs	Active pharmaceutical ingredients

## References

[1] Siegel R.L., Miller K.D., Jemal A. (2020). Cancer statistics, 2020. CA A Cancer J. Clin..

[2] Reck M., Rabe K.F. (2017). Precision Diagnosis and Treatment for Advanced Non–Small-Cell Lung Cancer. N. Engl. J. Med..

[3] Collins L.G., Haines C., Perkel R., Enck E.R. (2007). Lung cancer: Diagnosis and management. Am. Fam. Physician.

[4] Mustafa M., Azizi A.J., Iiizam E., Nazirah A., Sharifa S., Abbas S. (2016). Lung Cancer: Risk Factors, Management, And Prognosis. IOSR J. Dent. Med Sci..

[5] Waqar S.N., Morgensztern D. (2017). Treatment advances in small cell lung cancer (SCLC). Pharmacol. Ther..

[6] Egorova K.S., Gordeev E.G., Ananikov V.P. (2017). Biological Activity of Ionic Liquids and Their Application in Pharmaceutics and Medicine. Chem. Rev..

[7] Lei Z., Chen B., Koo Y.-M., MacFarlane D.R. (2017). Introduction: Ionic Liquids. Chem. Rev..

[8] Gomes J.M., Silva S.S., Reis R.L. (2019). Biocompatible ionic liquids: Fundamental behaviours and applications. Chem. Soc. Rev..

[9] Ding Y.-S., Zha M., Zhang J., Wang S.-S. (2007). Synthesis, characterization and properties of geminal imidazolium ionic liquids. Colloids Surf. A Physicochem. Eng. Asp..

[10] Steudte S., Bemowsky S., Mahrova M., Bottin-Weber U., Tojo-Suarez E., Stepnowski P., Stolte S. (2014). Toxicity and biodegradability of dicationic ionic liquids. RSC Adv..

[11] Payagala T., Huang J., Breitbach Z.S., Sharma P.S., Armstrong D.W. (2007). Unsymmetrical Dicationic Ionic Liquids: Manipulation of Physicochemical Properties Using Specific Structural Architectures. Chem. Mater..

[12] Sharma P.S., Payagala T., Wanigasekara E., Wijeratne A., Huang J., Armstrong D.W. (2008). Trigonal Tricationic Ionic Liquids: Molecular Engineering of Trications to Control Physicochemical Properties. Chem. Mater..

[13] Balducci A. (2017). Ionic Liquids in Lithium-Ion Batteries. Topics in Current Chemistry Collections.

[14] Earle M.J., Seddon K.R. (2000). Ionic liquids. Green solvents for the future. Pure Appl. Chem..

[15] Plechkova N.V., Seddon K.R. (2008). Applications of ionic liquids in the chemical industry. Chem. Soc. Rev..

[16] Rezki N., Al-Sodies S.A., Aouad M.R., Bardaweel S., Messali M., El Ashry E.S.H. (2016). An Eco-Friendly Ultrasound-Assisted Synthesis of Novel Fluorinated Pyridinium Salts-Based Hydrazones and Antimicrobial and Antitumor Screening. Int. J. Mol. Sci..

[17] Rezki N., Al-Sodies S.A., Shreaz S., Shiekh R.A., Messali M., Raja V., Aouad M.R. (2017). Green Ultrasound versus Conventional Synthesis and Characterization of Specific Task Pyridinium Ionic Liquid Hydrazones Tethering Fluorinated Counter Anions: Novel Inhibitors of Fungal Ergosterol Biosynthesis. Molecules.

[18] Rezki N., Messali M., Al-Sodies S.A., Naqvi A., Bardaweel S.K., Al-Blewi F.F., Aouad M.R., El Ashry E.S.H. (2018). Design, synthesis, in-silico and in-vitro evaluation of di-cationic pyridinium ionic liquids as potential anticancer scaffolds. J. Mol. Liq..

[19] Al-Blewi F., Rezki N., Naqvi A., Uddin H.Q., Al-Sodies S., Messali M., Aouad M.R., Bardaweel S. (2019). A Profile of the In Vitro Anti-Tumor Activity and In Silico ADME Predictions of Novel Benzothiazole Amide-Functionalized Imidazolium Ionic Liquids. Int. J. Mol. Sci..

[20] Ferraz R., Branco L., Prudêncio C., Noronha J.P., Petrovski Ž. (2011). Ionic Liquids as Active Pharmaceutical Ingredients. ChemMedChem.

[21] Turguła A., Stęsik K., Materna K., Klejdysz T., Praczyk T., Pernak J. (2020). Third-generation ionic liquids with N-alkylated 1,4-diazabicyclo[2.2.2]octane cations and pelargonate anions. RSC Adv..

[22] Stolte S., Matzke M., Arning J., Böschen A., Pitner W.-R., Welz-Biermann U., Jastorff B., Ranke J. (2007). Effects of different head groups and functionalised side chains on the aquatic toxicity of ionic liquids. Green Chem..

[23] Kumari1 P., Pillai1 V.V.S., Benedetto A. (2020). Mechanisms of action of ionic liquids on living cells: The state of the art. Biophys. Rev..

[24] Frade R.F., Rosatella A., Marques C., Branco L., Kulkarni P., Mateus N.M.M., Afonso C.A.M., Duarte C.M. (2009). Toxicological evaluation on human colon carcinoma cell line (CaCo-2) of ionic liquids based on imidazolium, guanidinium, ammonium, phosphonium, pyridinium and pyrrolidinium cations. Green Chem..

[25] Frade R.F.M., Matias A., Branco L.C., Afonso C.A.M., Duarte C.M.M. (2007). Effect of ionic liquids on human colon carcinoma HT-29 and CaCo-2 cell lines. Green Chem..

[26] Padnya P., Andreyko E.A., Gorbatova P.A., Parfenov V.V., Rizvanov I.K., Stoikov I.I. (2017). Towards macrocyclic ionic liquids: Novel ammonium salts based on tetrasubstituted p-tert-butylthiacalix[4]arenes. RSC Adv..

[27] Pérez S.A., Montalbán M.G., Carissimi G., Licence P., Víllora G.J. (2020). In vitro cytotoxicity assessment of monocationic and dicationic pyridinium-based ionic liquids on HeLa, MCF-7, BGM and EA.hy926 cell lines. J. Hazard. Mater..

[28] Vereshchagin A., Frolov N., Egorova K., Seitkalieva M., Ananikov V. (2021). Quaternary Ammonium Compounds (QACs) and Ionic Liquids (ILs) as Biocides: From Simple Antiseptics to Tunable Antimicrobials. Int. J. Mol. Sci..

[29] Lima P.C., Lima L.M., da Silva K.C.M., Léda P.H.O., de Miranda A.L.P., Fraga C.A., Barreiro E.J. (2000). Synthesis and analgesic ac-tivity of novel N-acylarylhydrazones and isosters, derived from natural safrole. Eur. J. Med. Chem..

[30] Al-Blewi F.F., Rezki N., Al-Sodies S.A., Bardaweel S.K., Sabbah D.A., Messali M., Aouad M.R. (2018). Novel amphiphilic pyridinium ionic liquids-supported Schiff bases: Ultrasound assisted synthesis, molecular docking and anticancer evaluation. Chem. Central J..

[31] Bardaweel S.K., Abu-Dahab R., Almomani N.F. (2013). An in vitro based investigation into the cytotoxic effects of D-amino acids. Acta Pharm..

[32] Bilginer S., Bardaweel S.K., Sabbah D.A., Gul H.I. (2021). Docking Studies and Antiproliferative Activities of 6-(3-aryl-2-propenoyl)-2(3H)- benzoxazolone Derivatives as Novel Inhibitors of Phosphatidylinositol 3-Kinase (PI3Kα). Anti-Cancer Agents Med. Chem..

[33] Bardaweel S.K., Alsalamat H., Aleidi S., Bashatwah R.M. (2018). Glucose Deprivation Enhances the Antiproliferative Effects of Oral Hypoglycemic Biguanides in Different Molecular Subtypes of Breast Cancer: An in Vitro Study. Acta Pharm..

