# Supplementary material for: Novel Dipyridinium Lipophile-Based Ionic Liquids Tethering Hydrazone Linkage: Design, Synthesis and Antitumorigenic Study
Source: Int J Mol Sci. 2021 Sep 28;22(19):10487. doi: 10.3390/ijms221910487 (PMC8508903; doi:10.3390/ijms221910487)
Supplement: Supplementary file 1 [file ijms-22-10487-s001.zip › ijms-1390612-supplementary.pdf]

Novel dipyridinium lipophiles based ionic liquids tethering hydrazone linkage: Design, Synthesis and antitumorigenic study

Salsabeel Al-Sodies[1], Nadjet Rezki[1,*], Fawzia Faleh Albelwi [1], Mouslim Messali[1], Mohamed R. Aouad[1], Sanaa K. Bardaweel[2,*], Mohamed Hagar[3]

[a]*Department of Chemistry, Faculty of Science, Taibah University, Al-Madinah Al-Munawarah 30002, Saudi Arabia*
[b]*Department of Pharmaceutical Sciences, Faculty of Pharmacy, University of Jordan, Amman 11942, Jordan*
[c]*Chemistry Department, Faculty of Science, Alexandria University, Alexandria 21321, Egypt*

Spectroscopic characterization

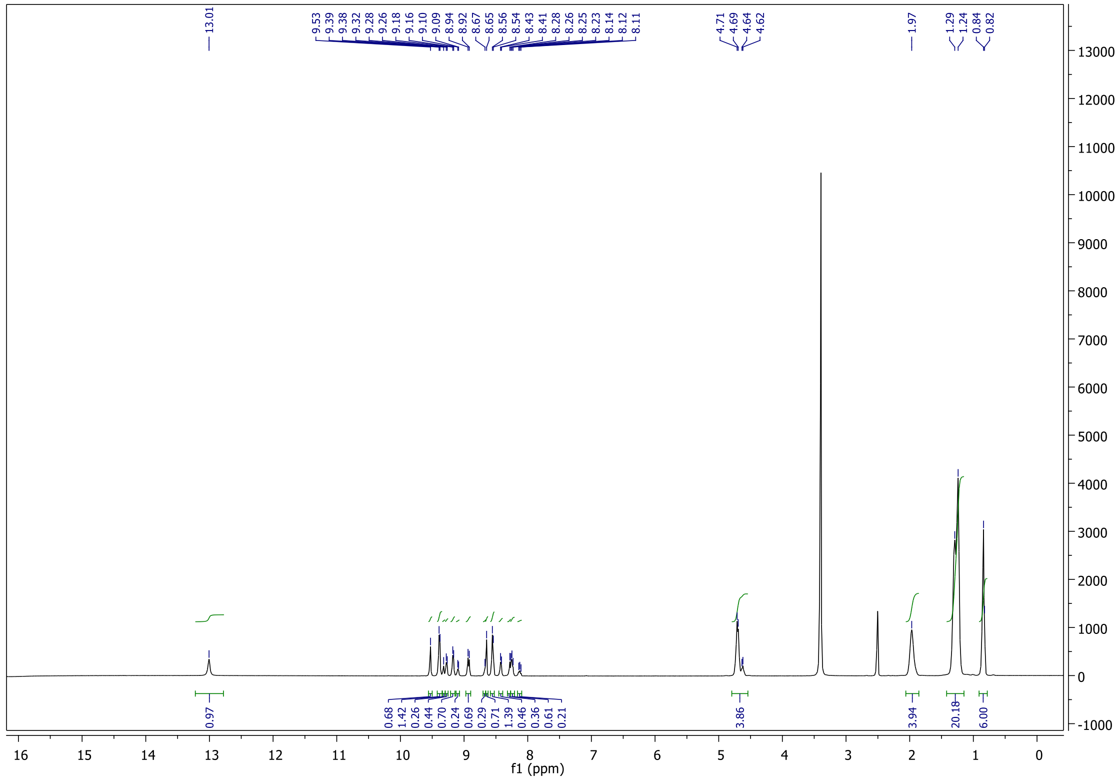

Figure S1. ^{1}H NMR of Compound **9**

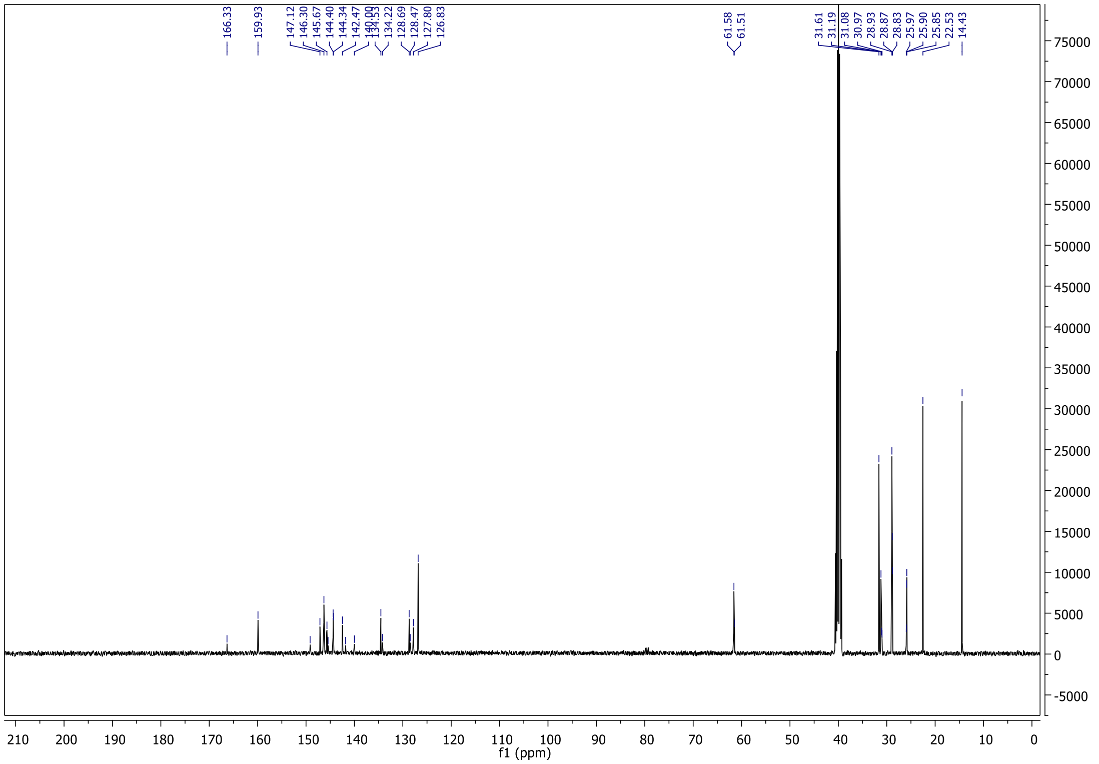

Figure S2. ^{13}C NMR of Compound **9**

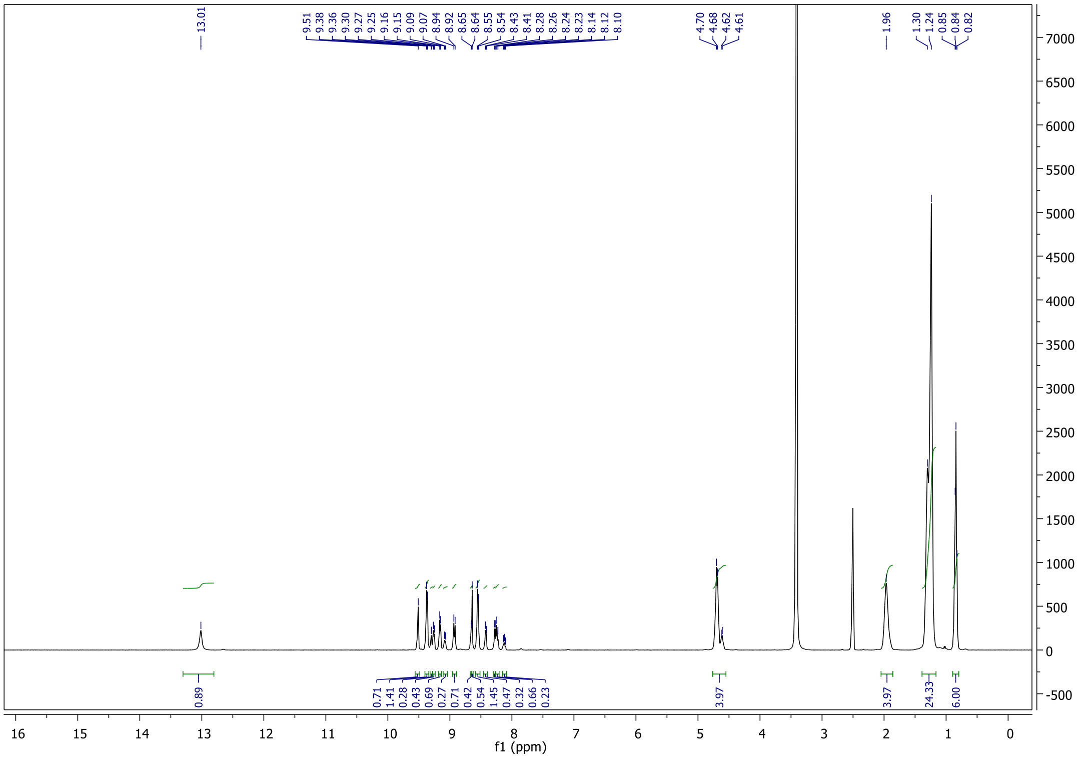

Figure S3. ^{1}H NMR of Compound **10**

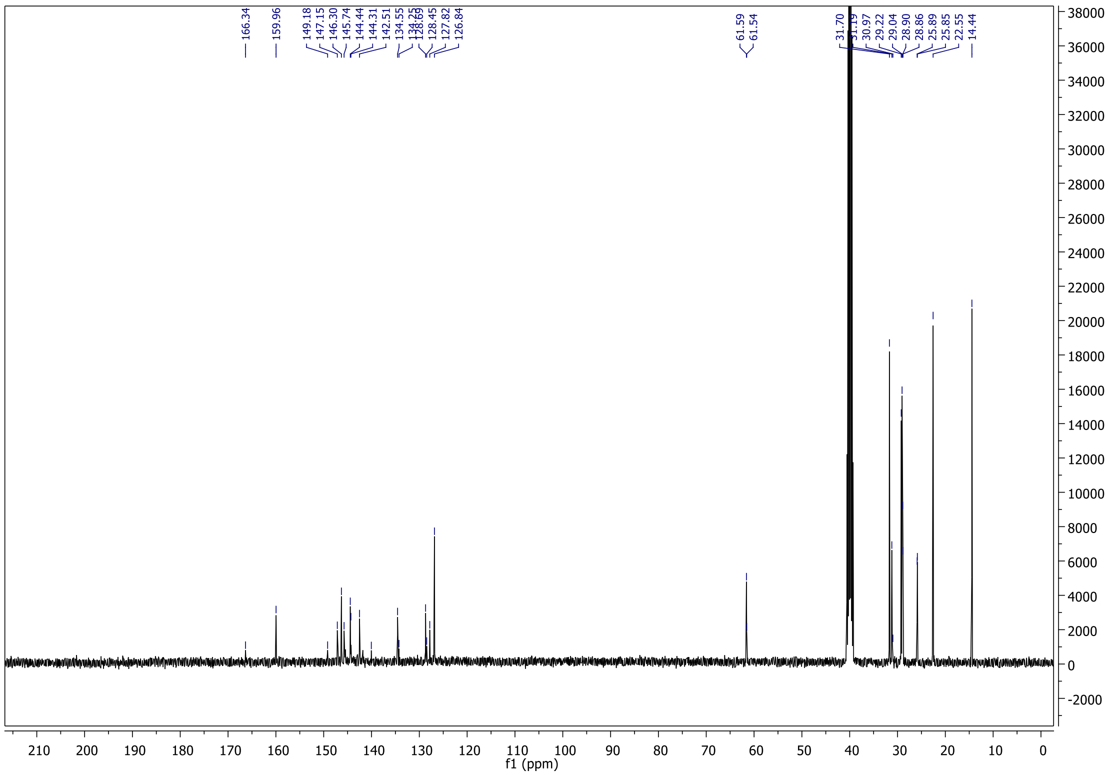

Figure S4. ^{13}C NMR of Compound **10**

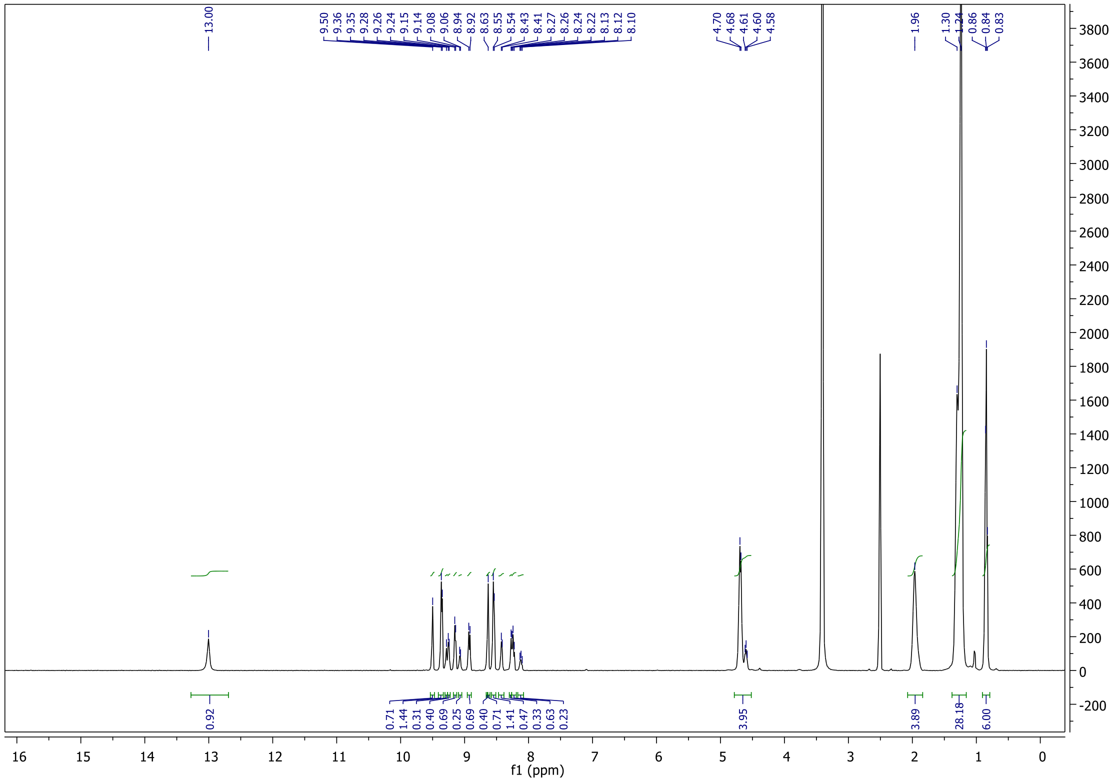

Figure S5. ^{1}H NMR of Compound **11**

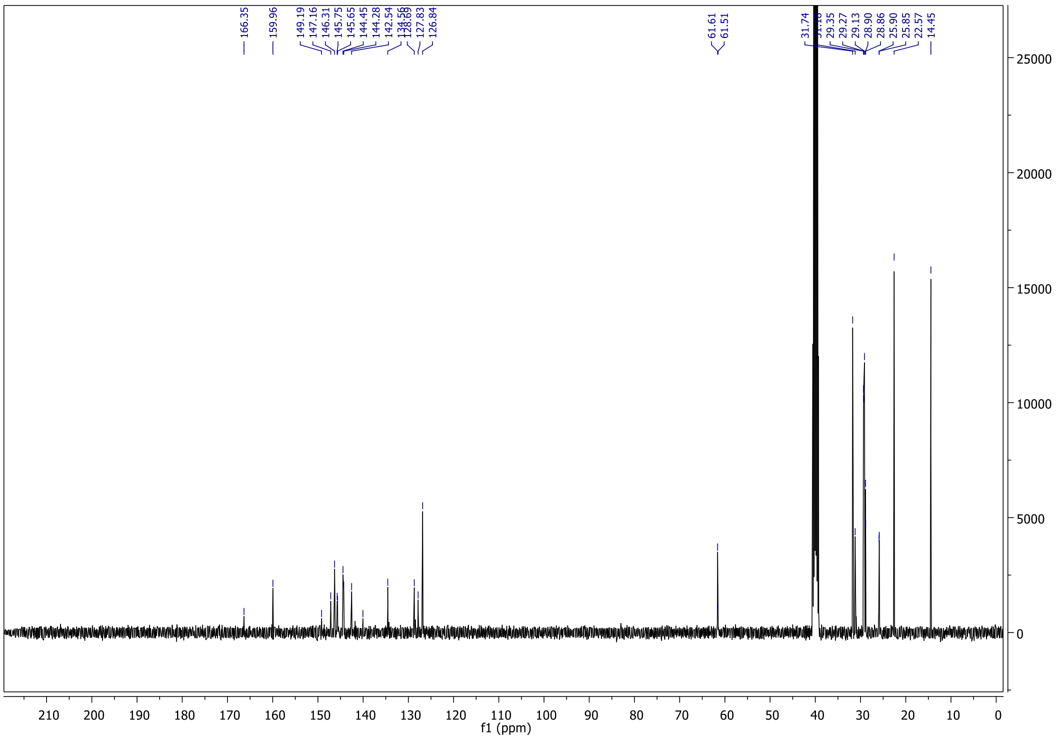

Figure S6. ^{13}C NMR of Compound **11**

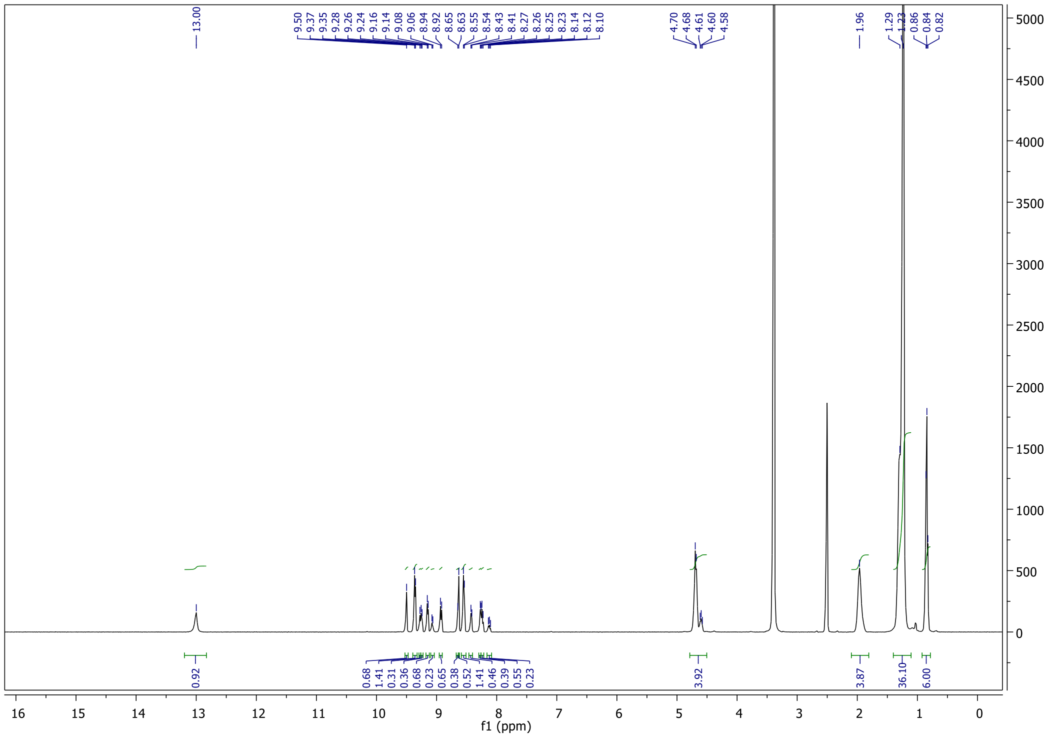

Figure S7. ^{1}H NMR of Compound **12**

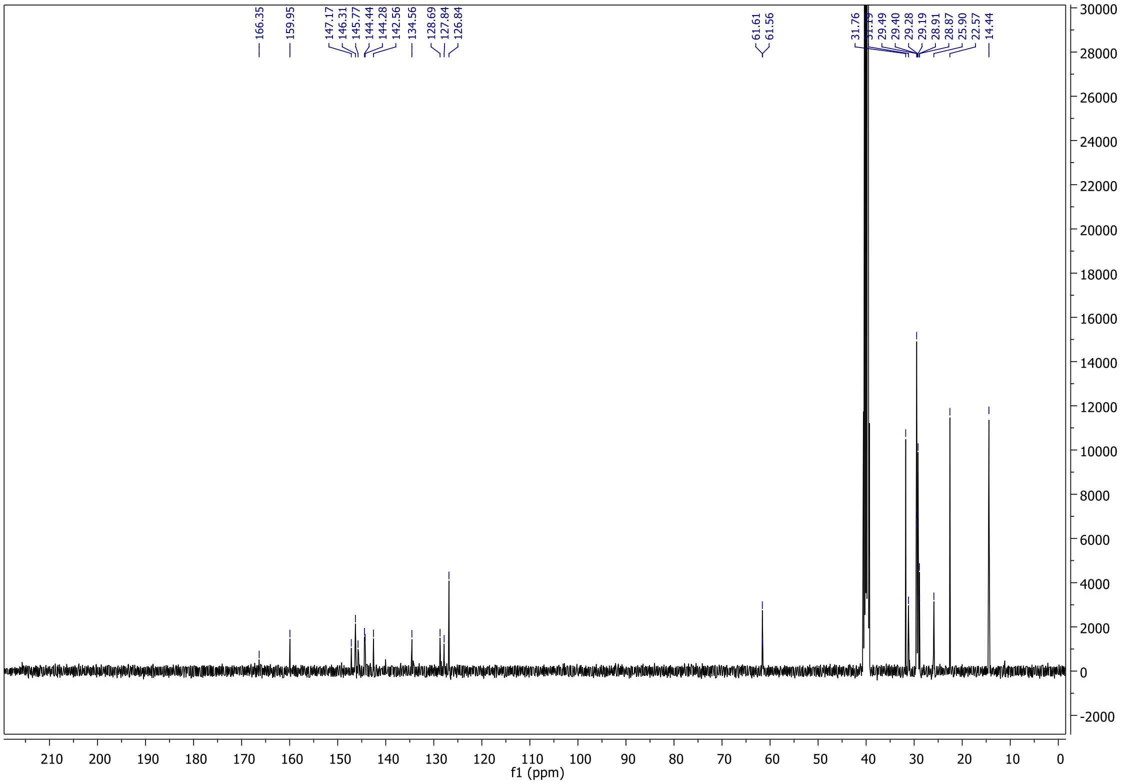

Figure S8. ^{13}C NMR of Compound **12**

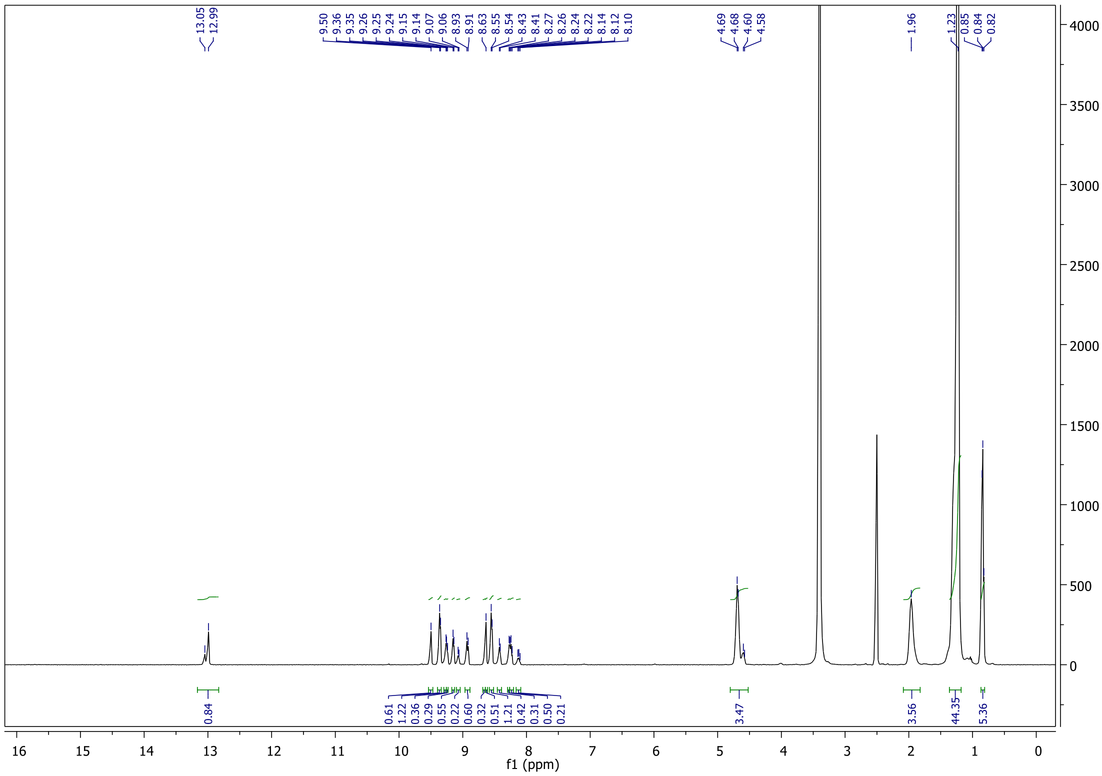

Figure S9. ^{1}H NMR of Compound **13**

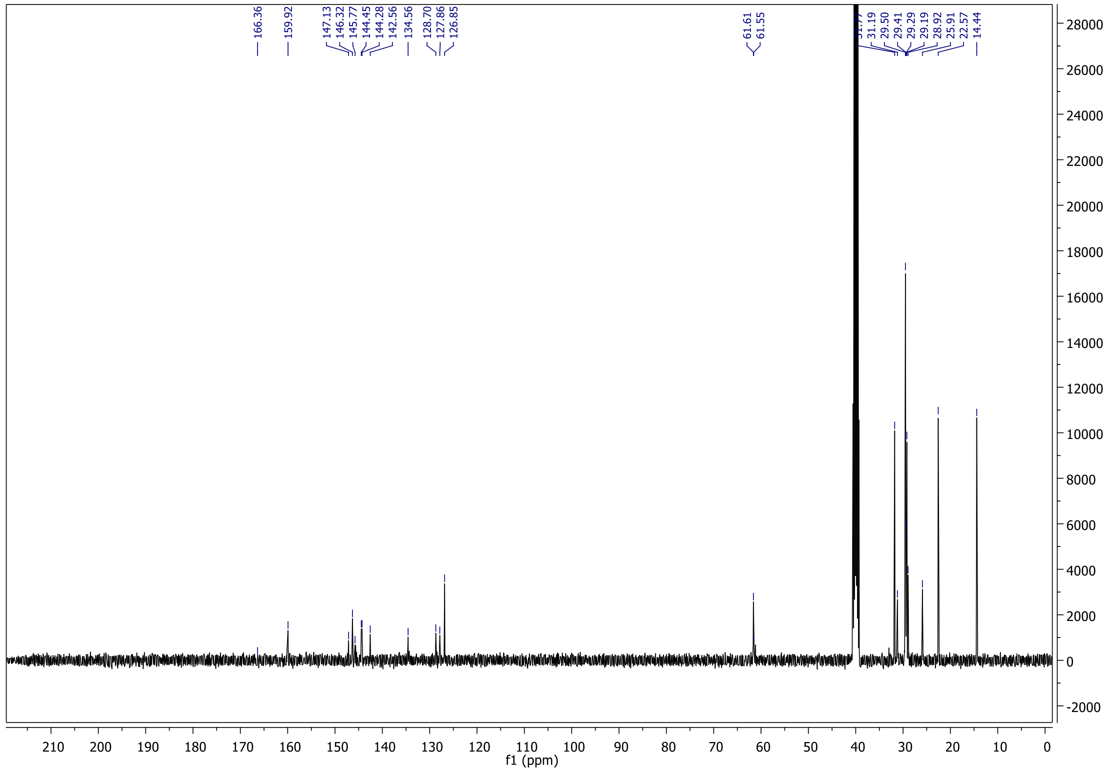

Figure S10. ^{13}C NMR of Compound **13**

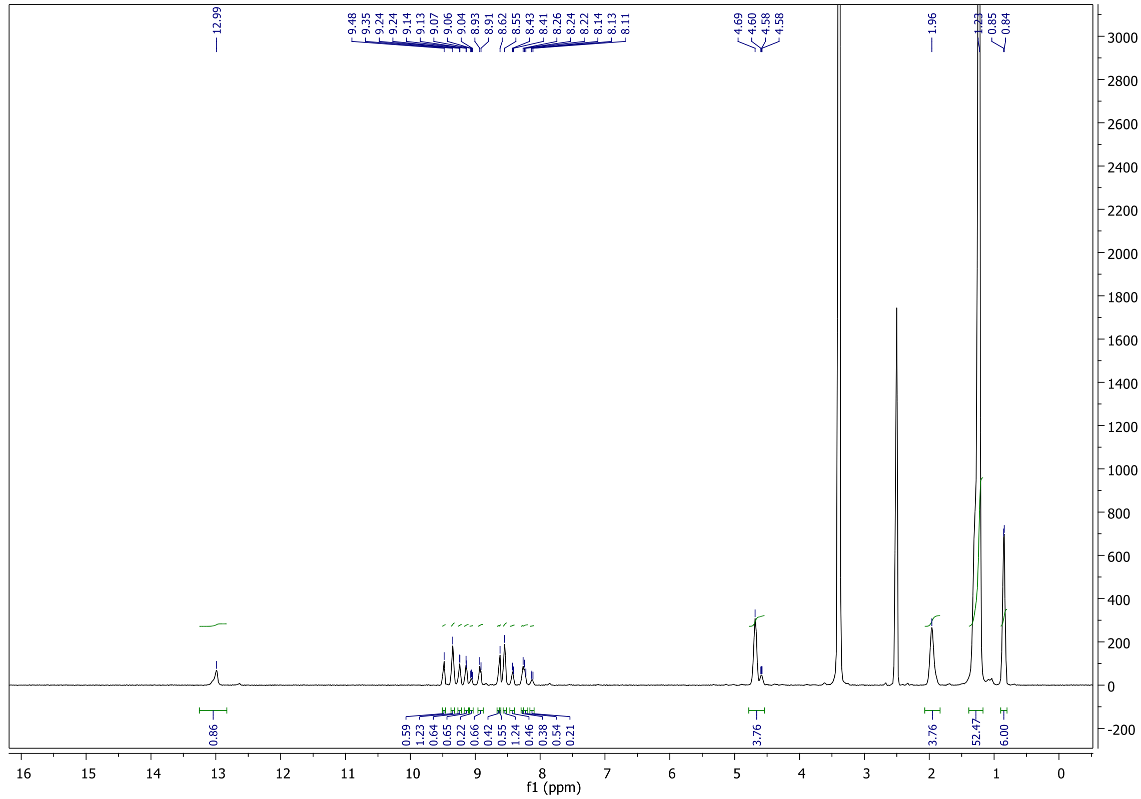

Figure S11. ^{1}H NMR of Compound **14**

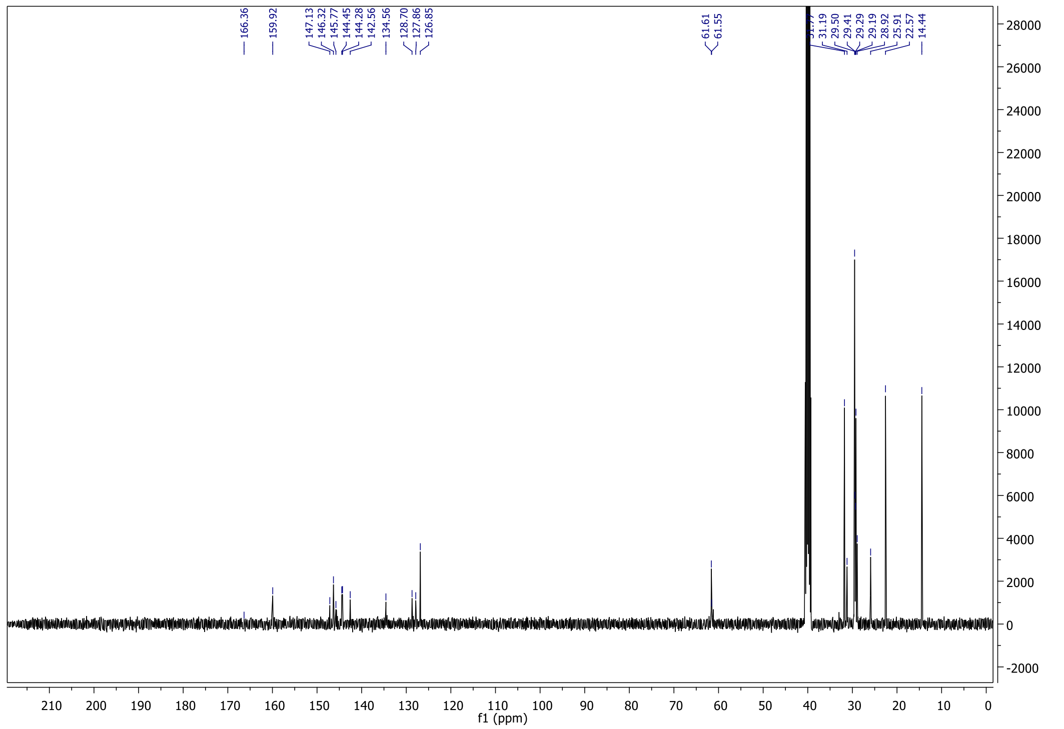

Figure S12. ^{13}C NMR of Compound **14**

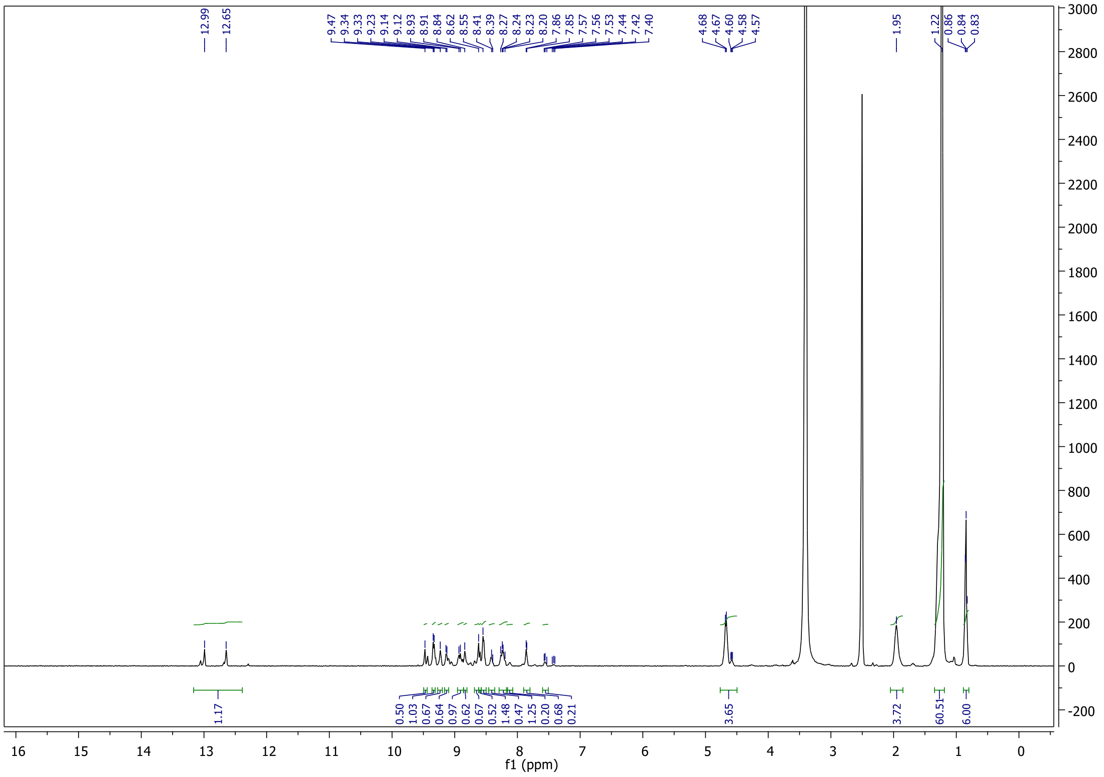

Figure S13. ^{1}H NMR of Compound **15**

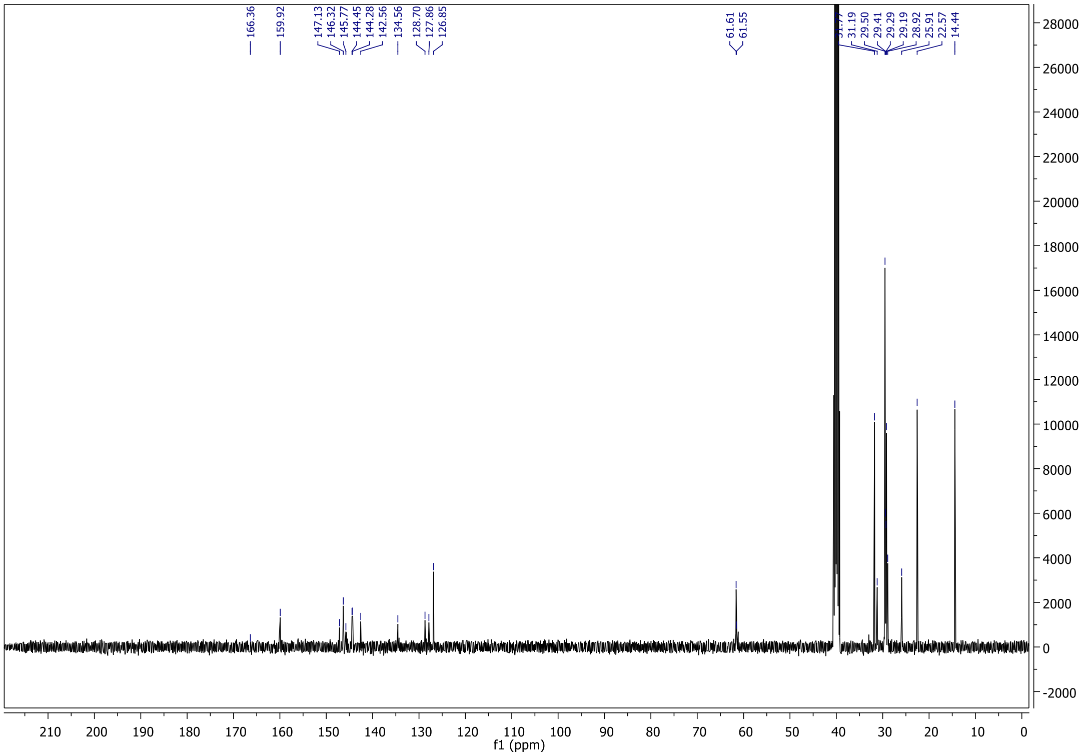

Figure S14. ^{13}C NMR of Compound **15**

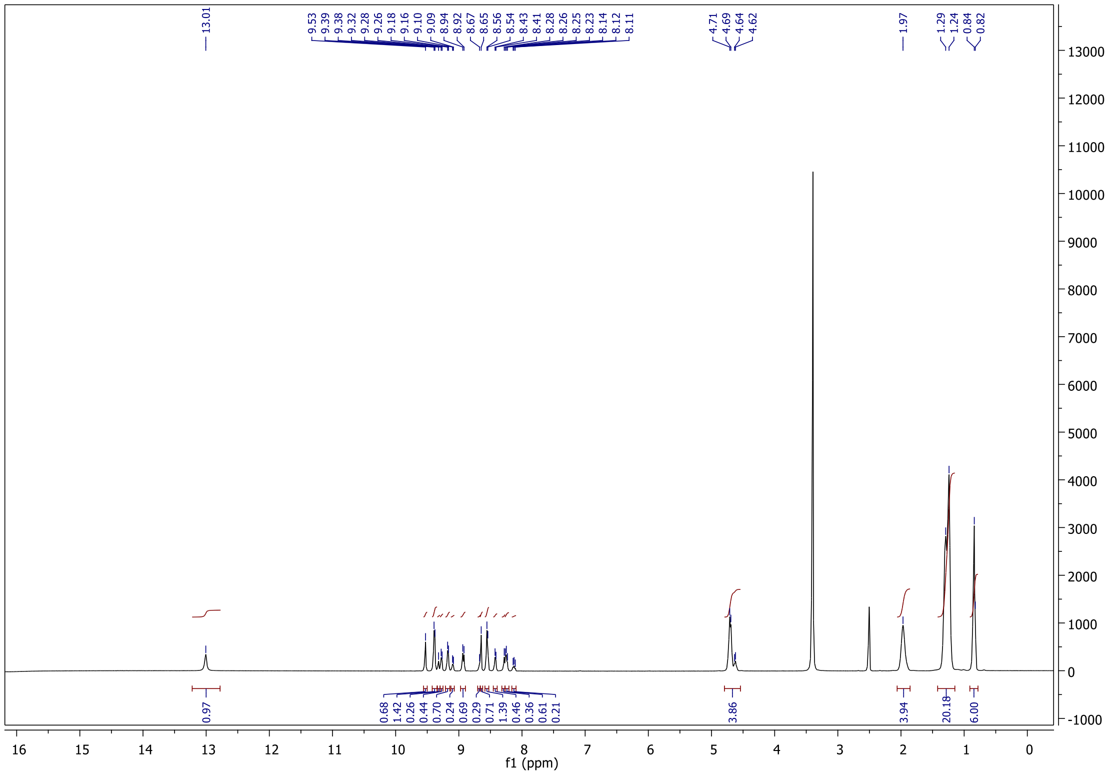

Figure S15. ^{1}H NMR of Compound **16**

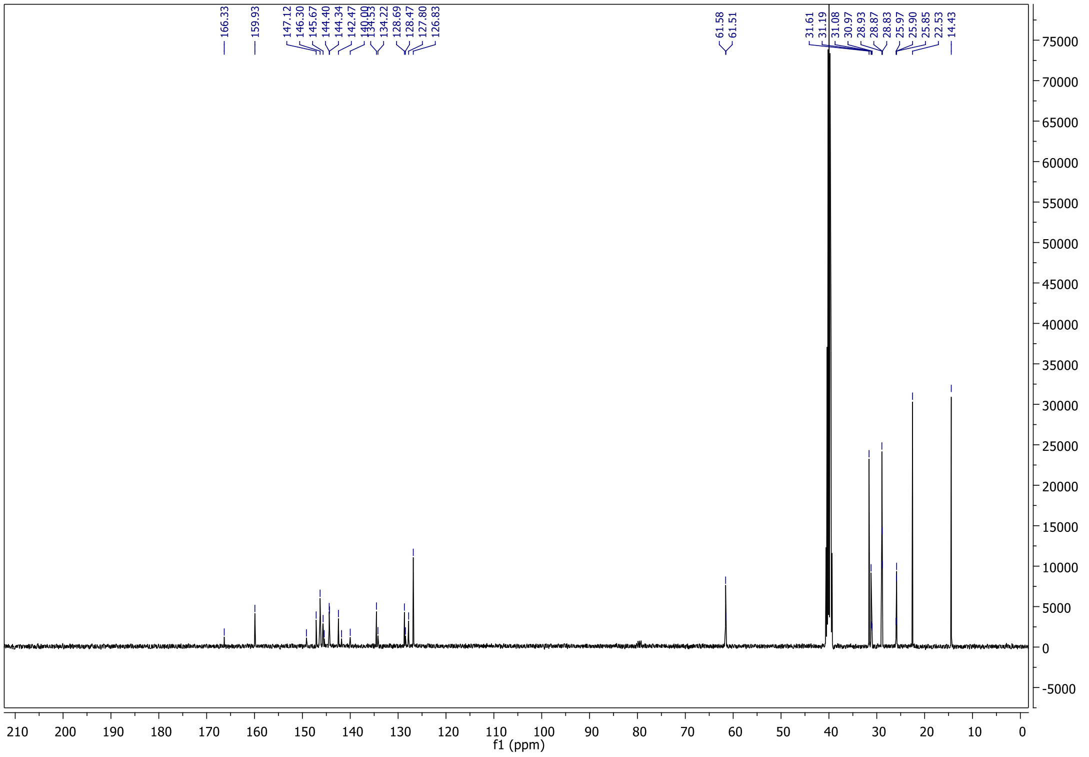

Figure S16. ^{13}C NMR of Compound **16**

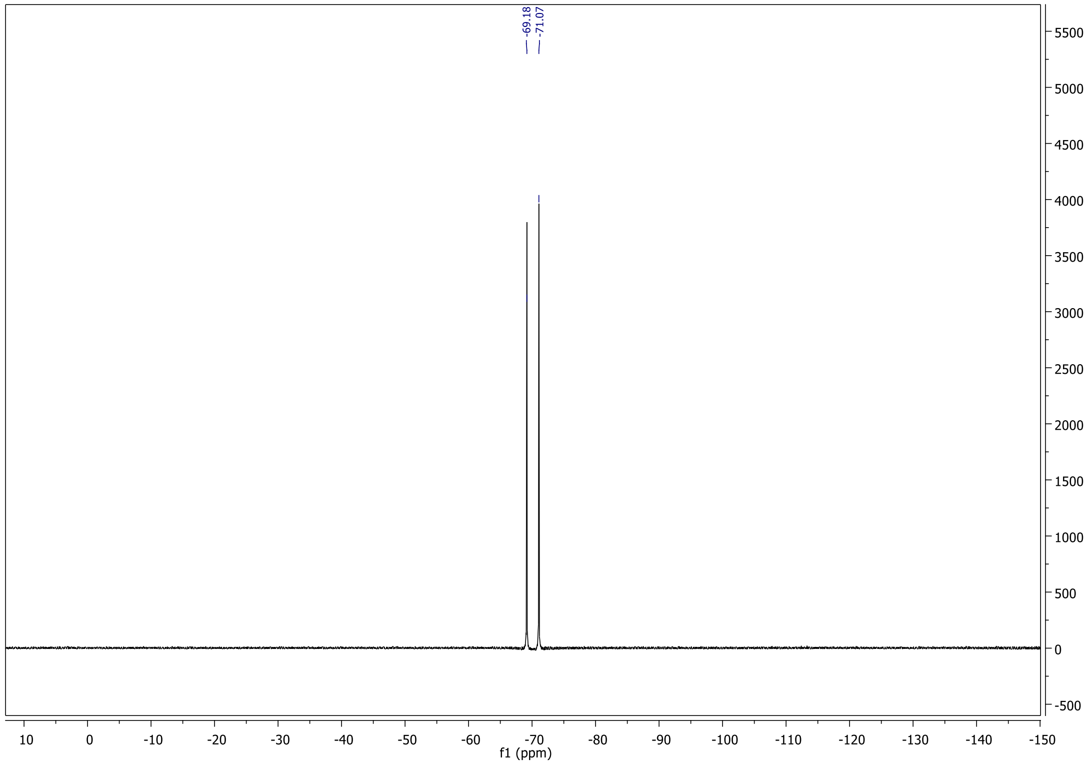

Figure S17. ^{19}F NMR of Compound **16**

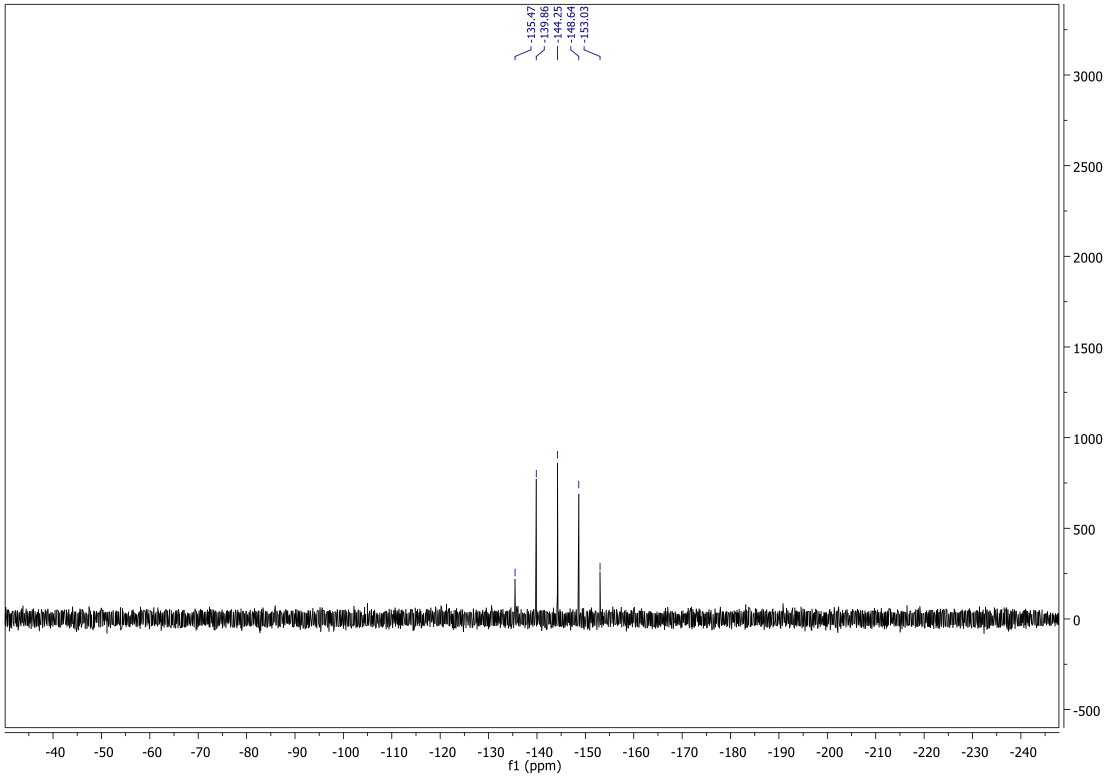

Figure S18. ^{31}P NMR of Compound **16**

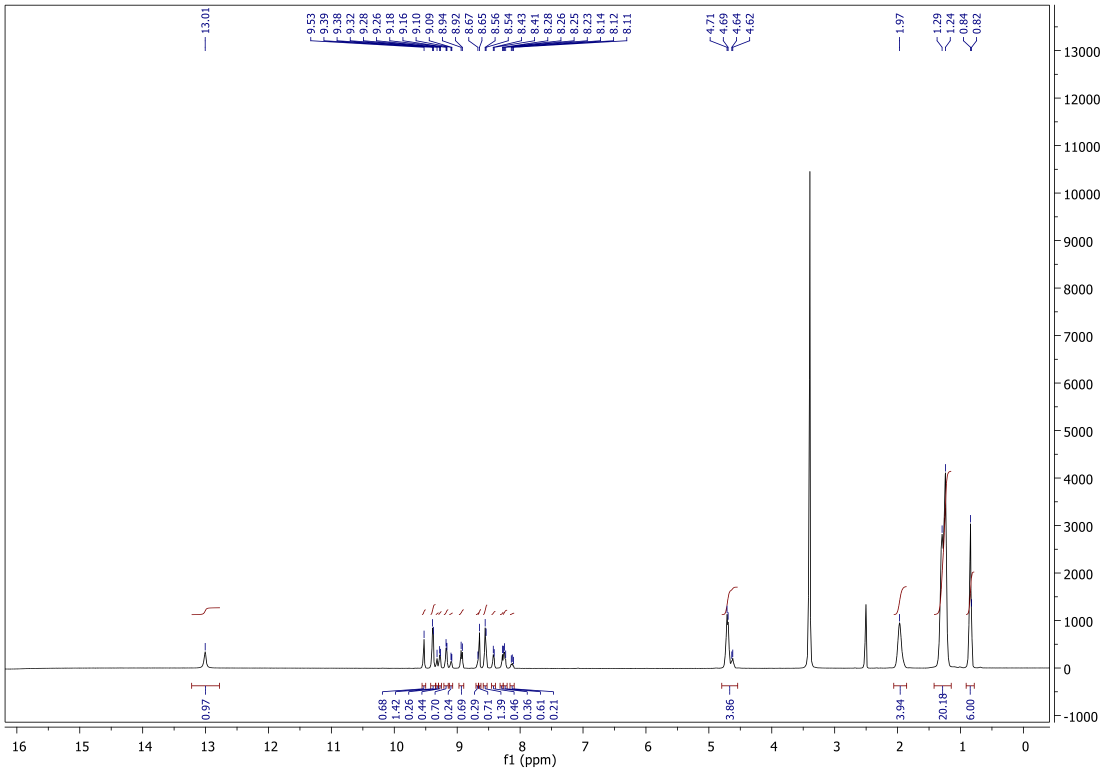

Figure S19. ^{1}H NMR of Compound **17**

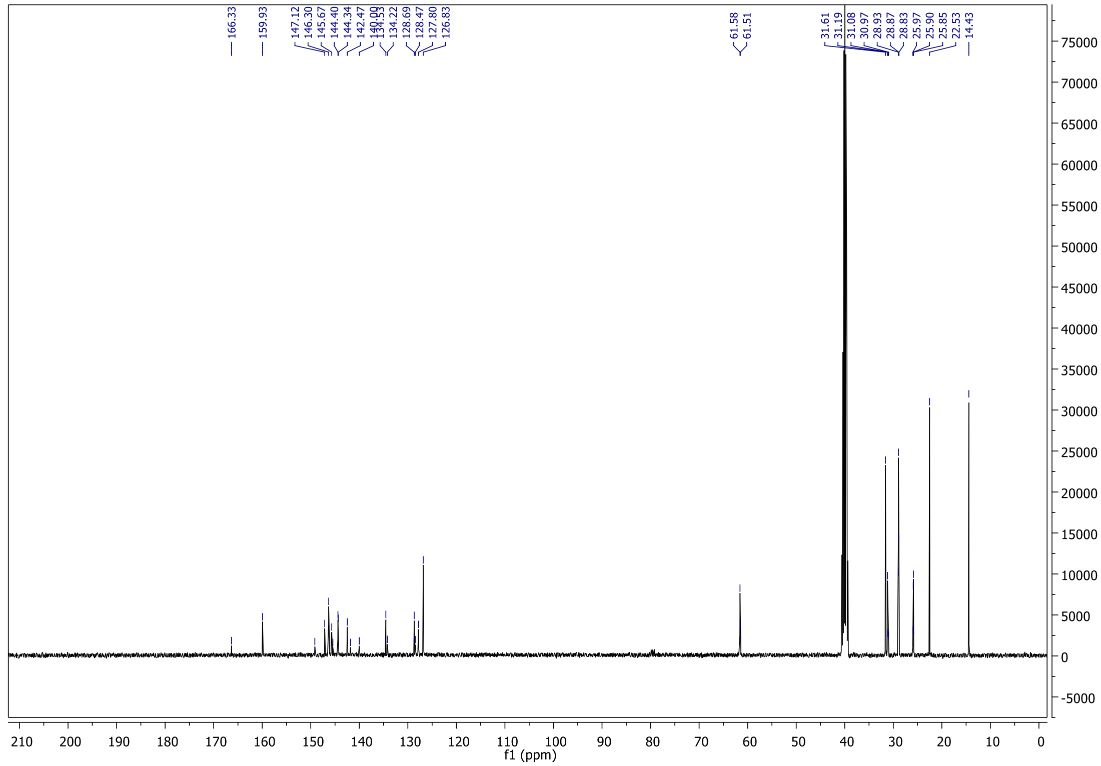

Figure S20. ^{13}CNMR of Compound **17**

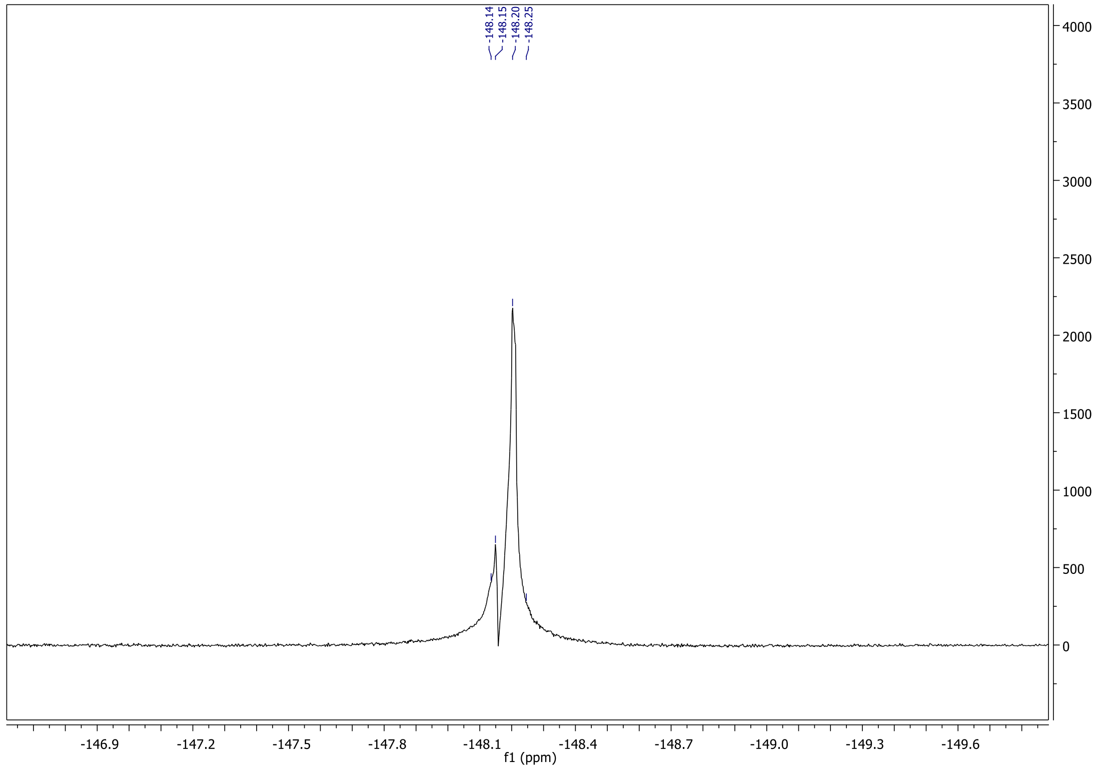

Figure S21. ^{19}F NMR of Compound **17**

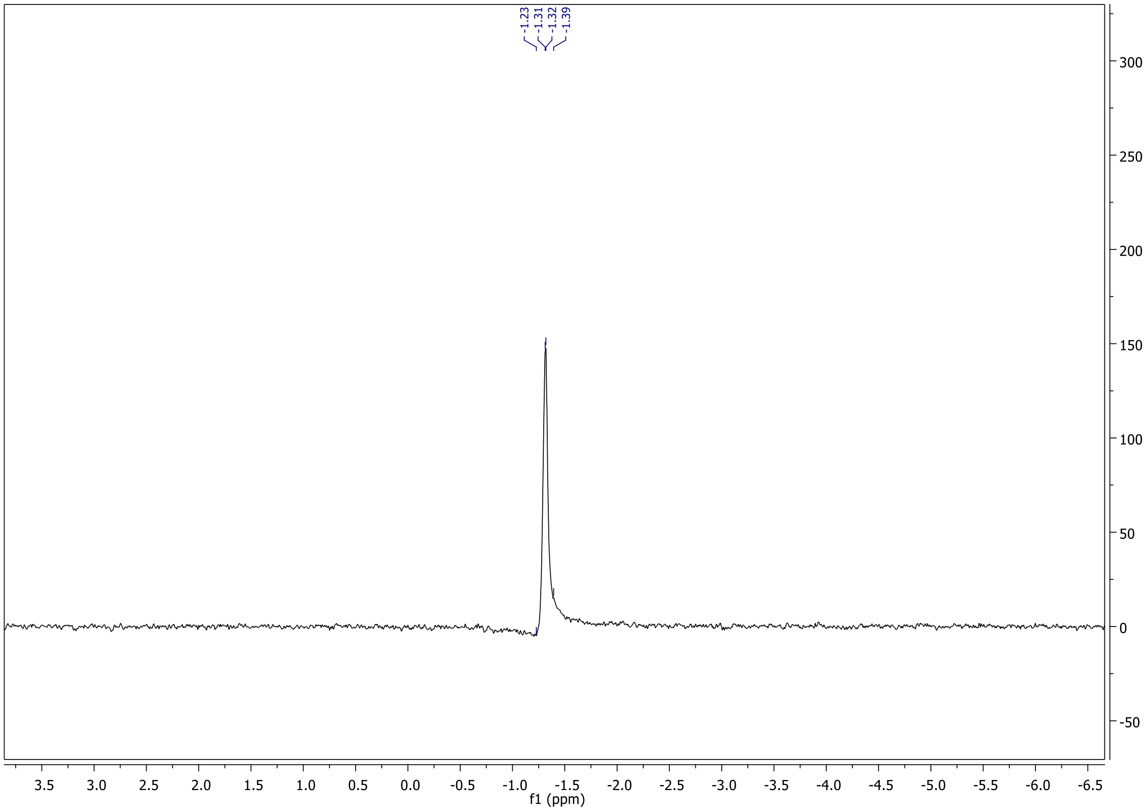

Figure S22. ^{11}B NMR of Compound **17**

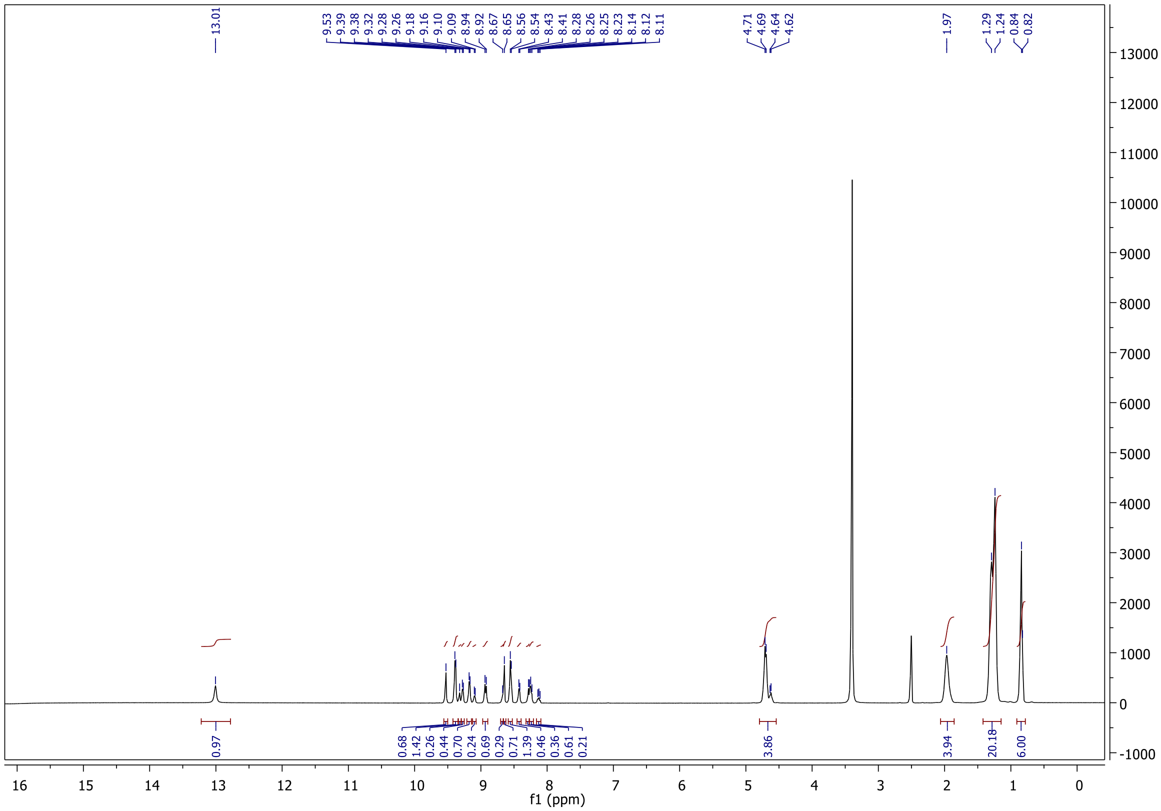

Figure S23. ^{1}H NMR of Compound **18**

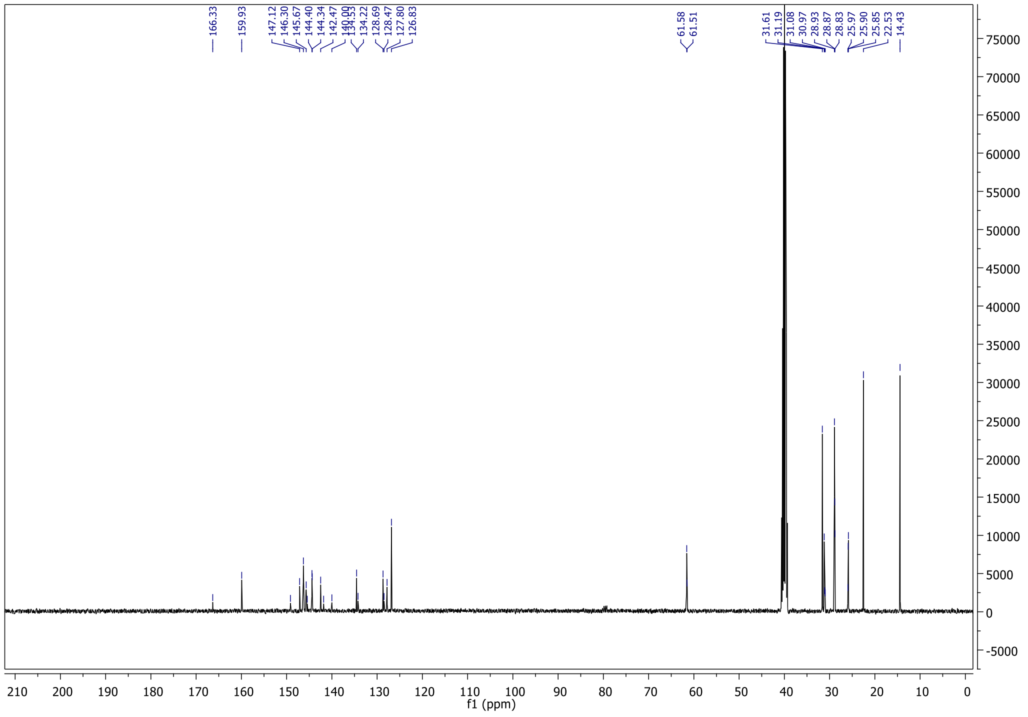

Figure S24. ^{13}C NMR of Compound **18**

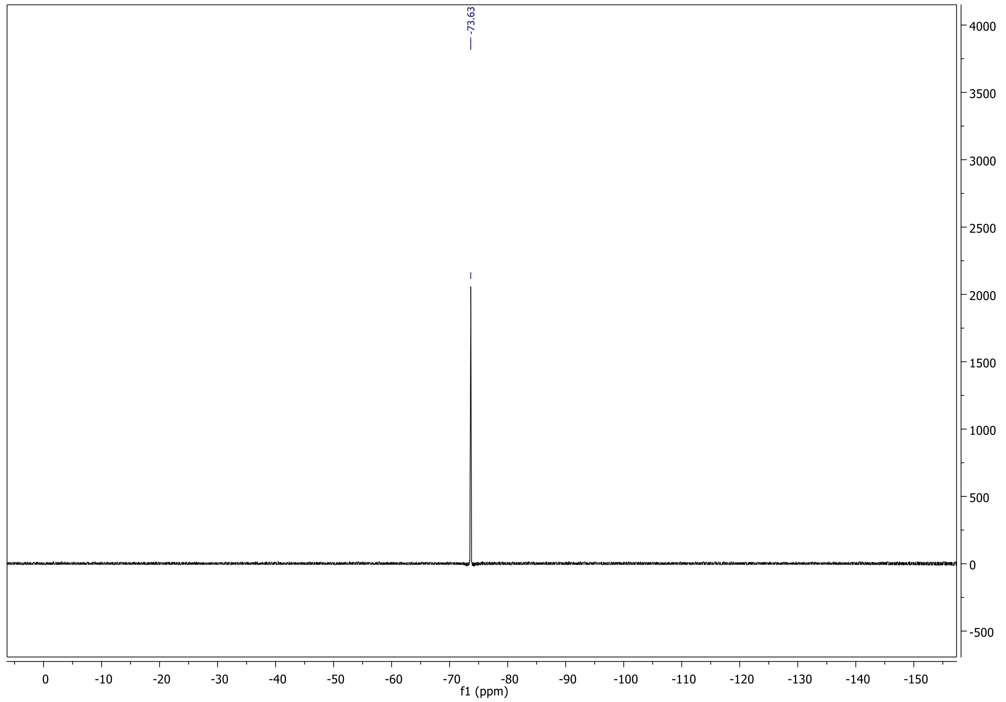

Figure S25. ^{19}F NMR of Compound **18**

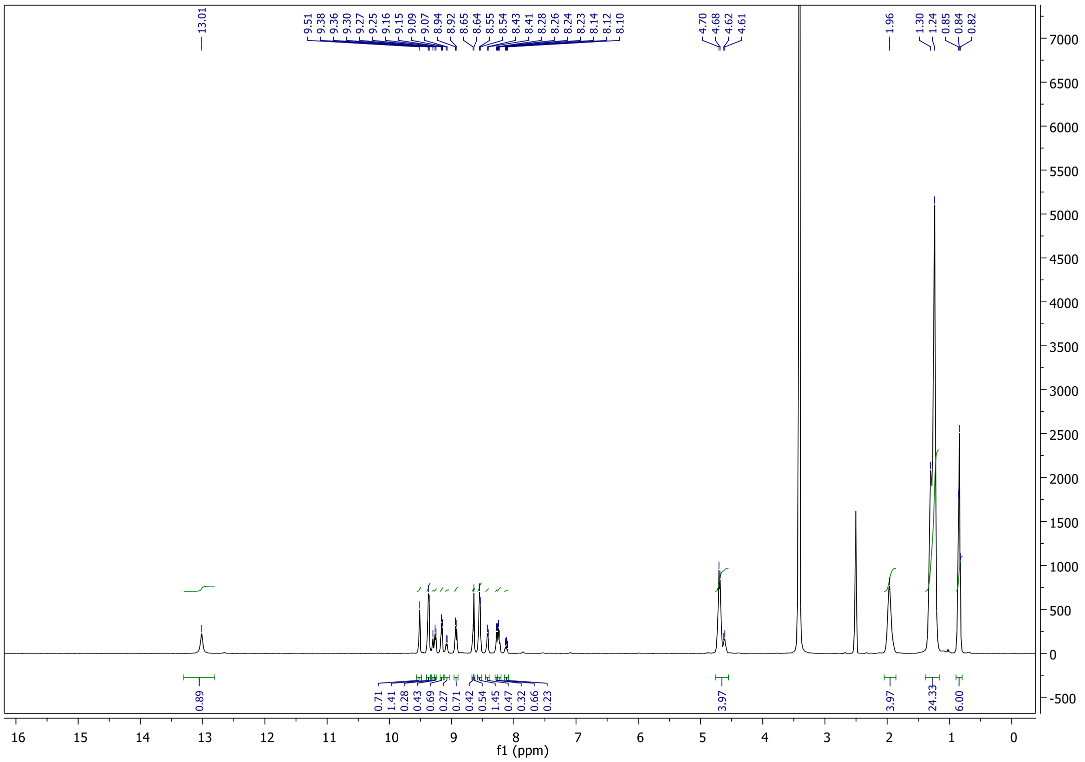

Figure S26. ^{1}H NMR of Compound **19**

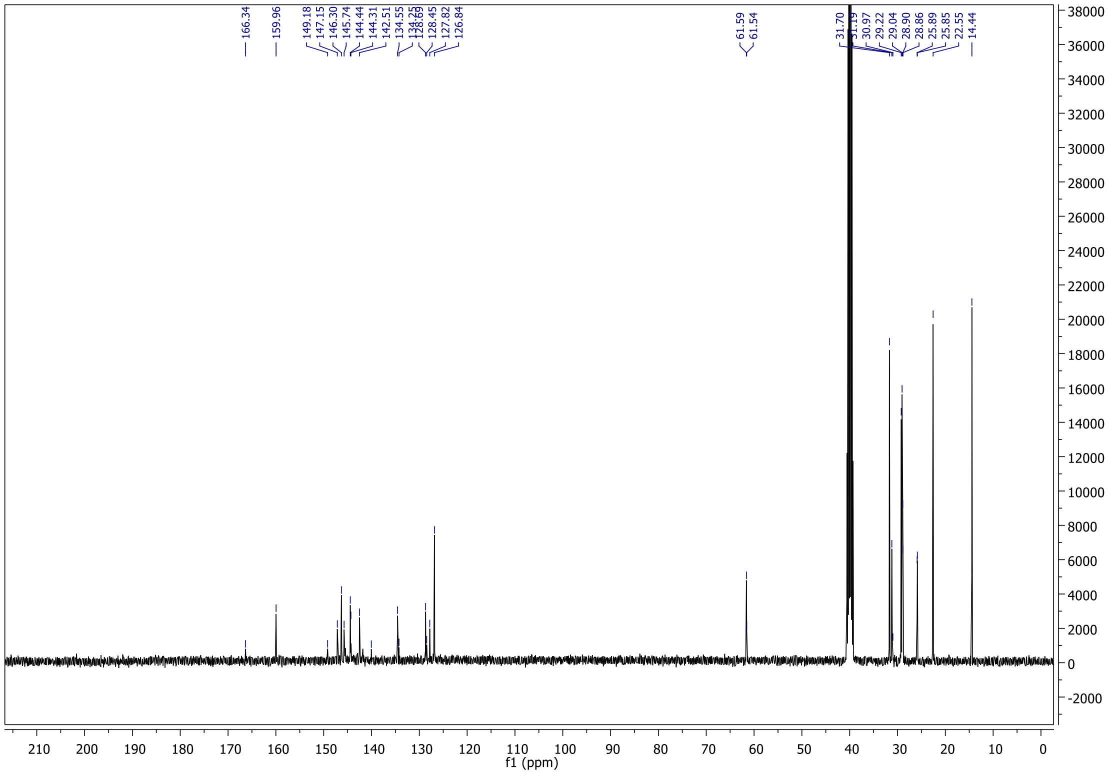

Figure S27. ¹³C NMR of Compound **19**

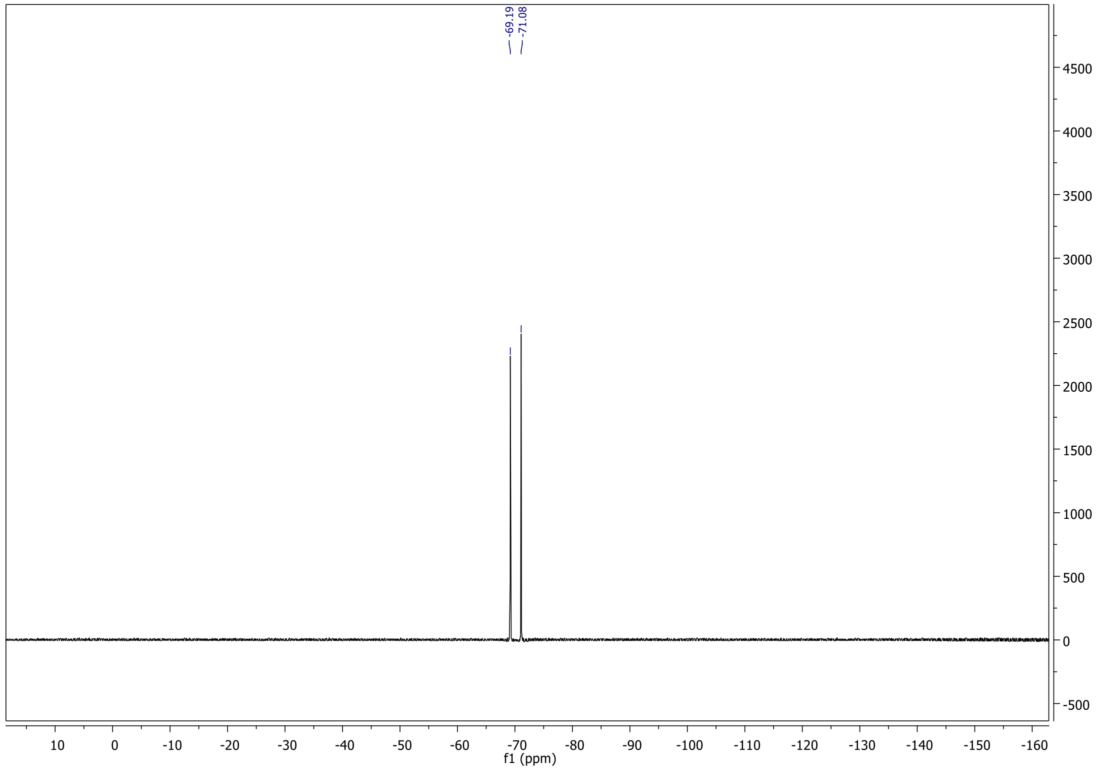

Figure S28. ¹⁹F NMR of Compound **19**

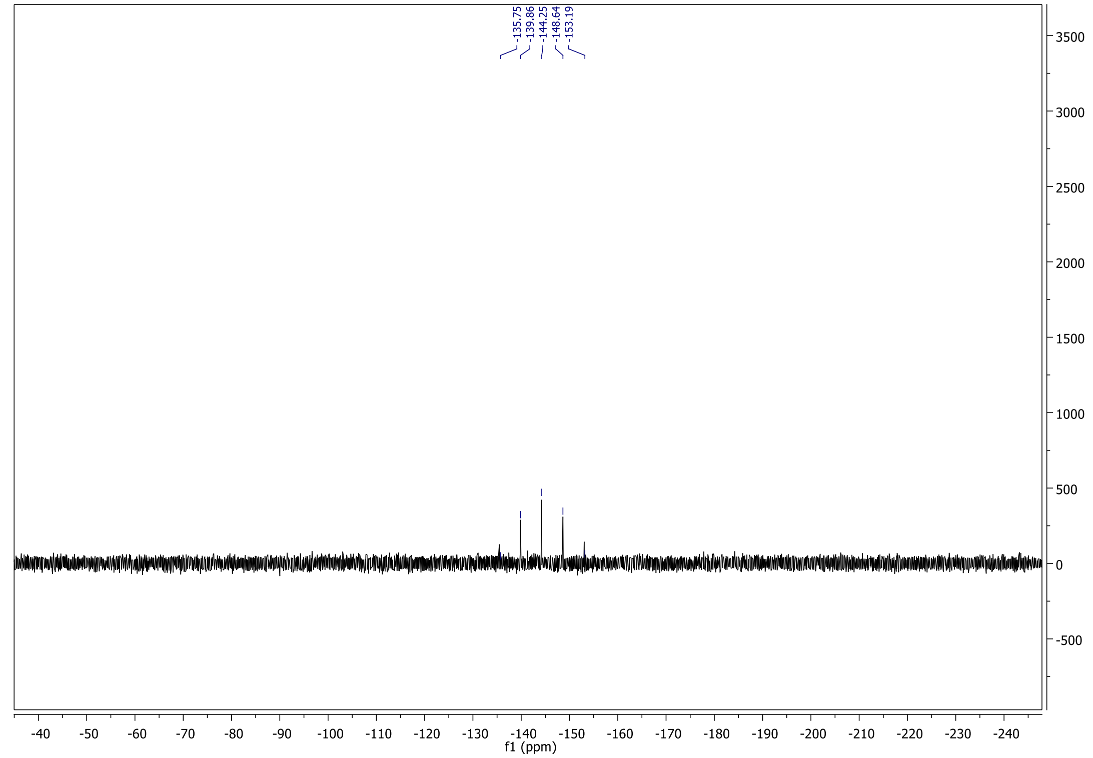

Figure S29. ^{31}P NMR of Compound **19**

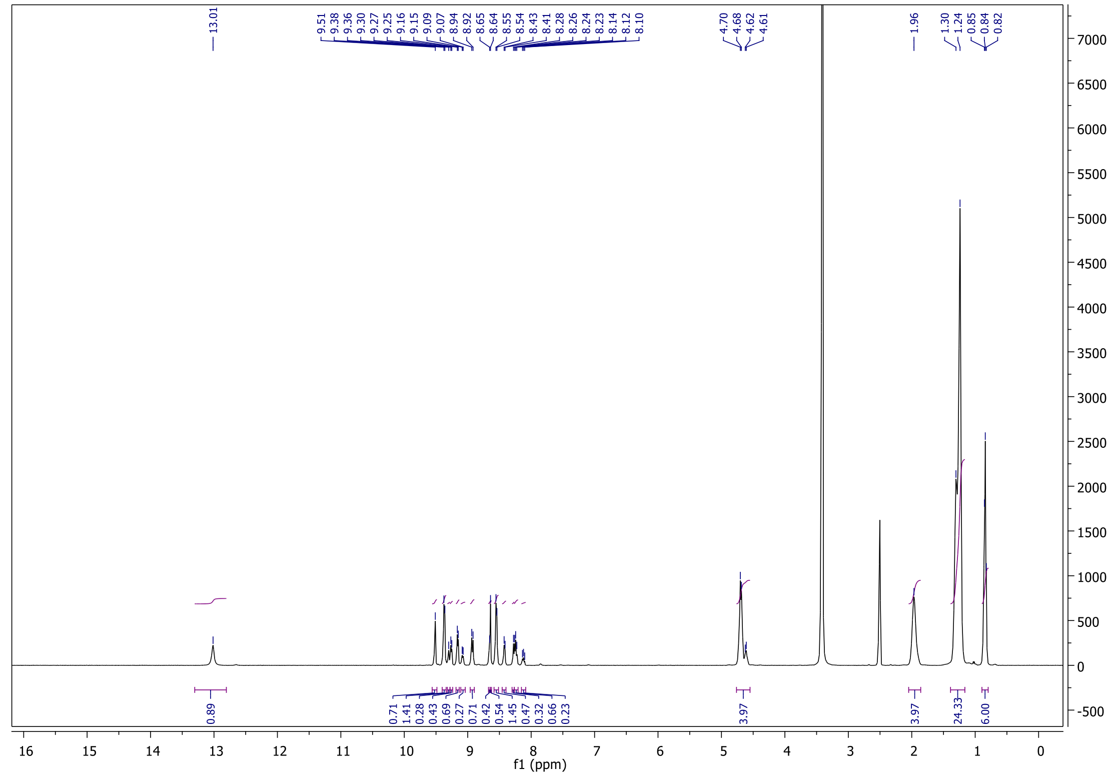

Figure S30. ^{1}H NMR of Compound **20**

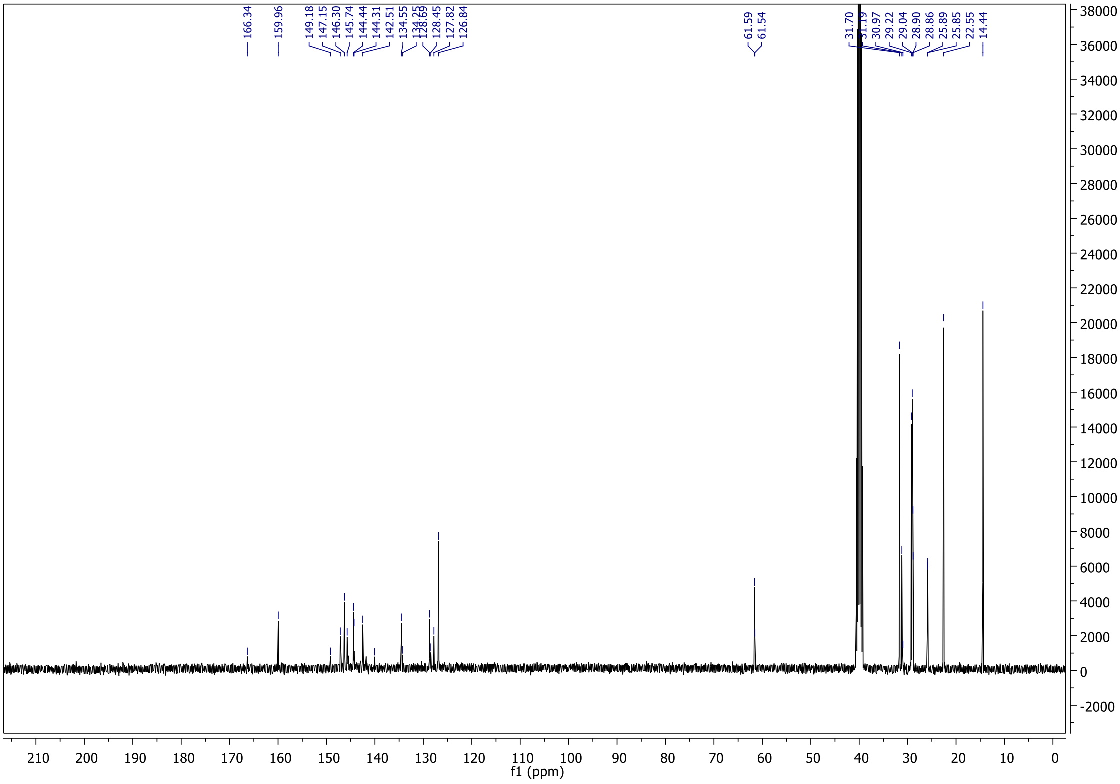

Figure S31. ^{13}C NMR of Compound **20**

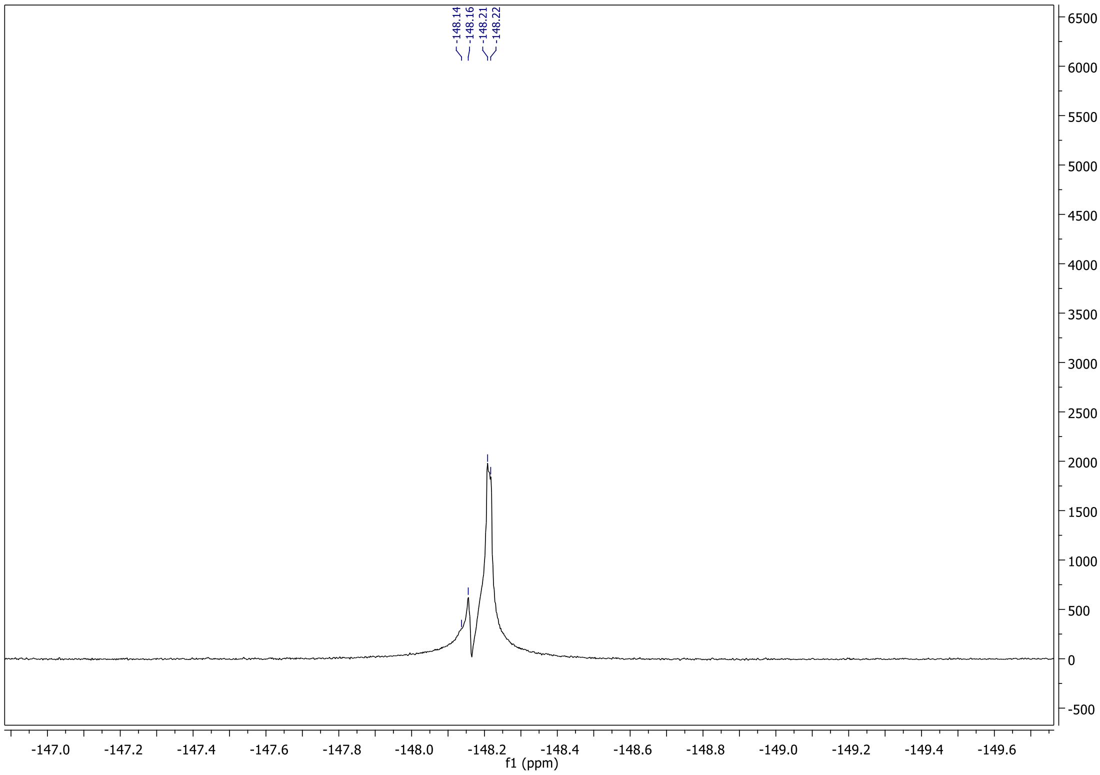

Figure S32. ^{19}F NMR of Compound **20**

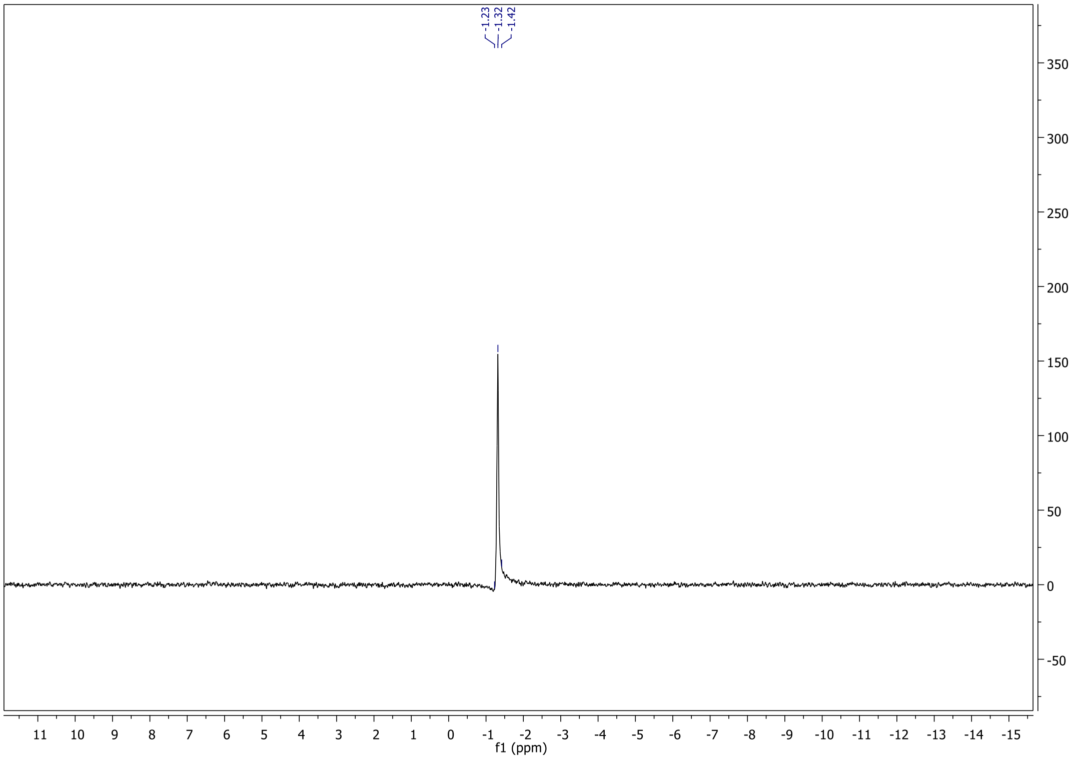

Figure S33. ^{11}B NMR of Compound **20**

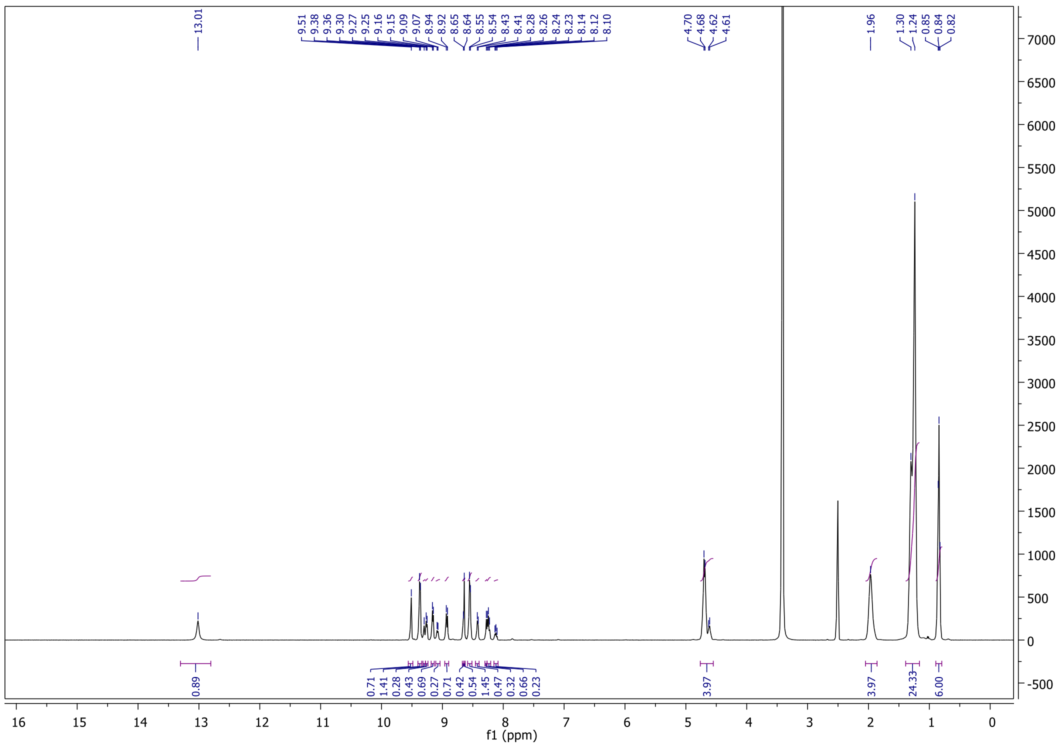

Figure S34. ^{1}H NMR of Compound **21**

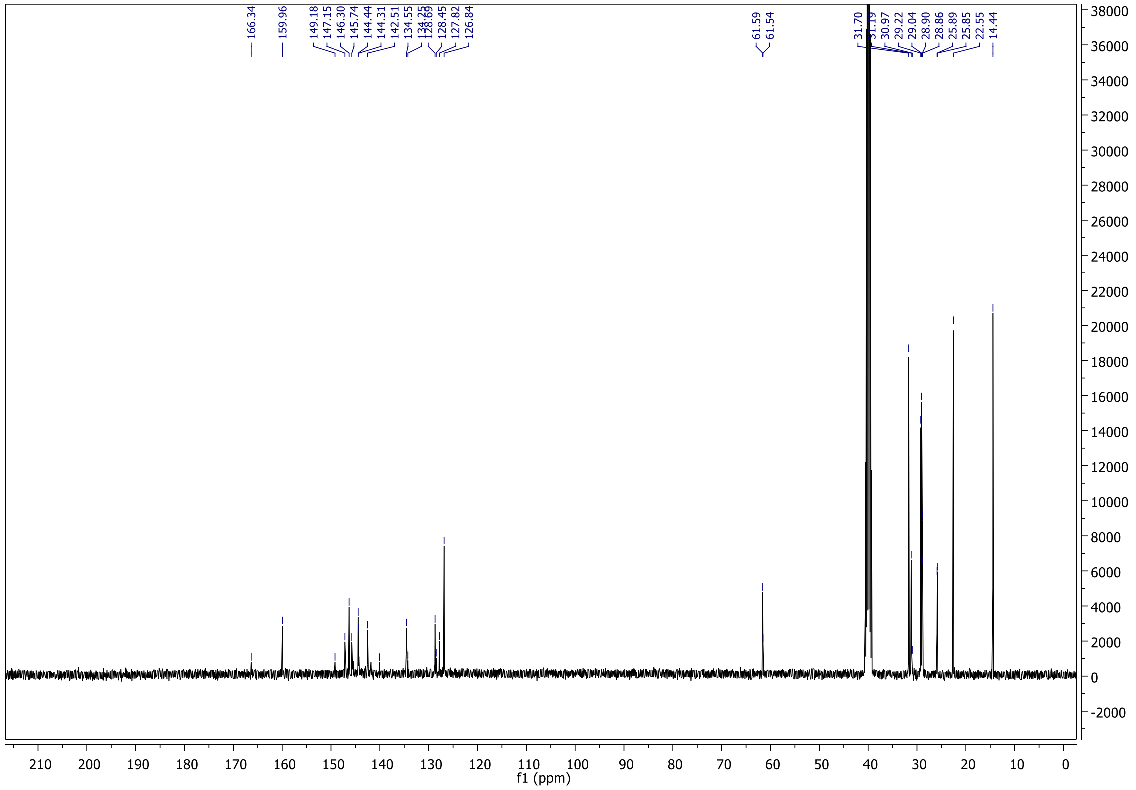

Figure S35. ^{13}C NMR of Compound **21**

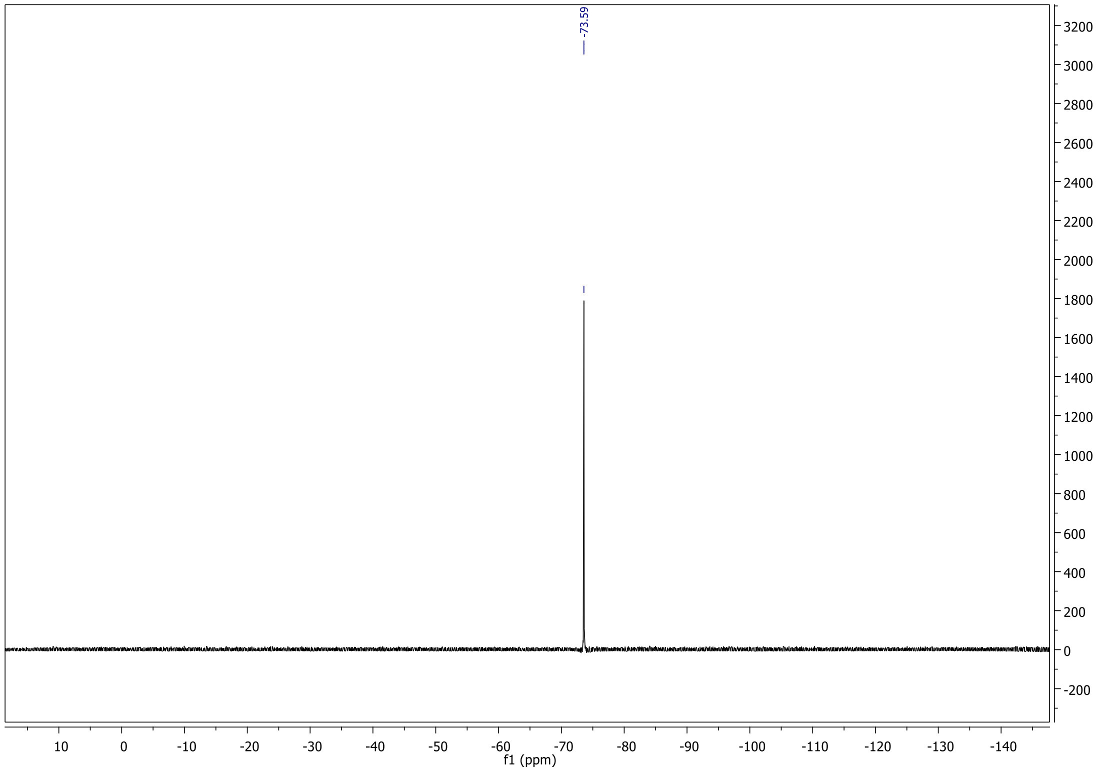

Figure S36. ^{19}F NMR of Compound **21**

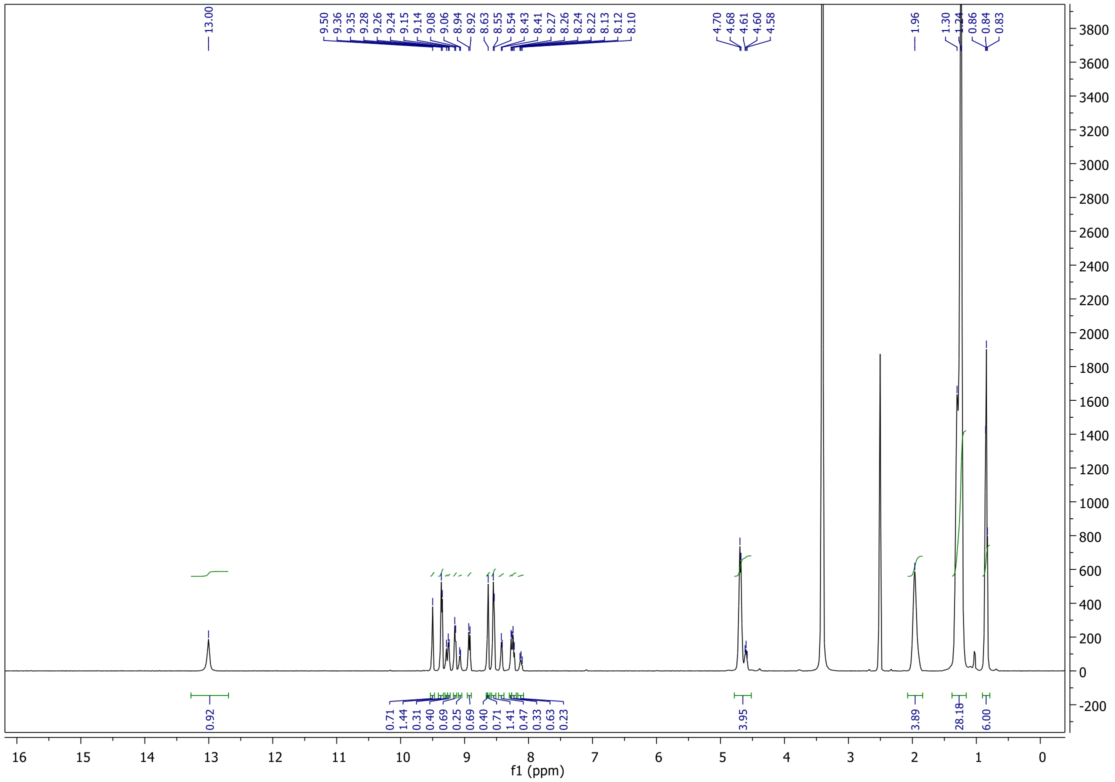

Figure S37. ^{1}H NMR of Compound **22**

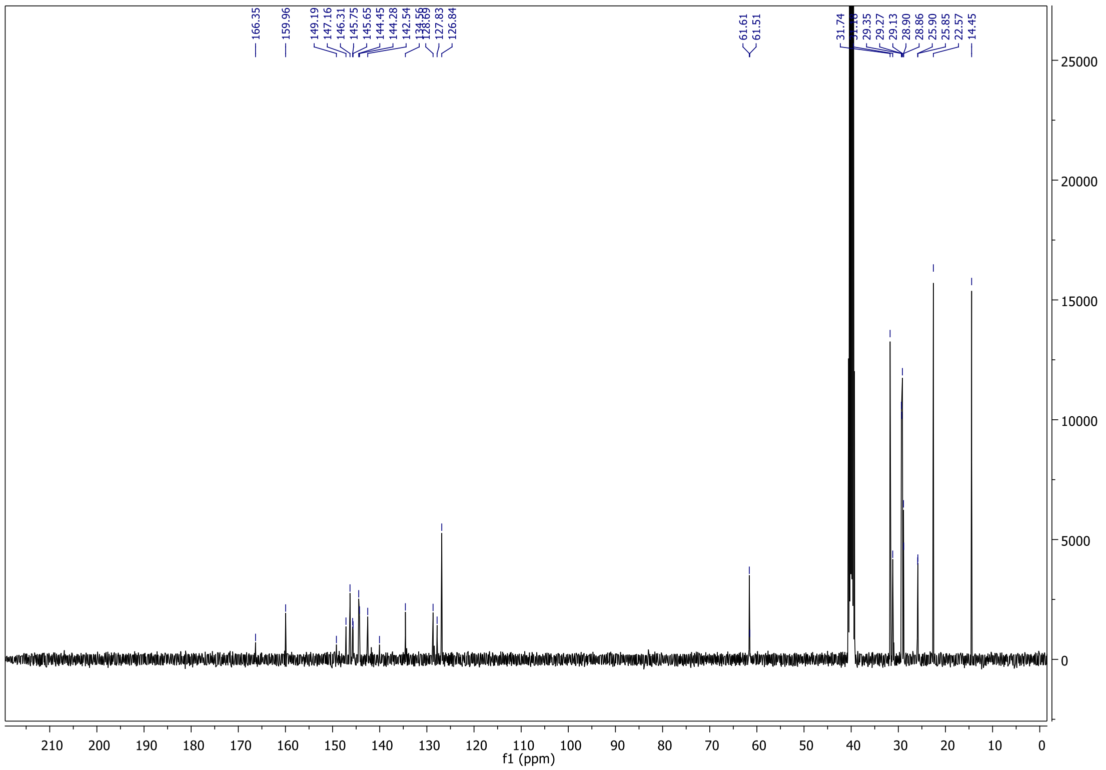

Figure S38. ^{13}C NMR of Compound **22**

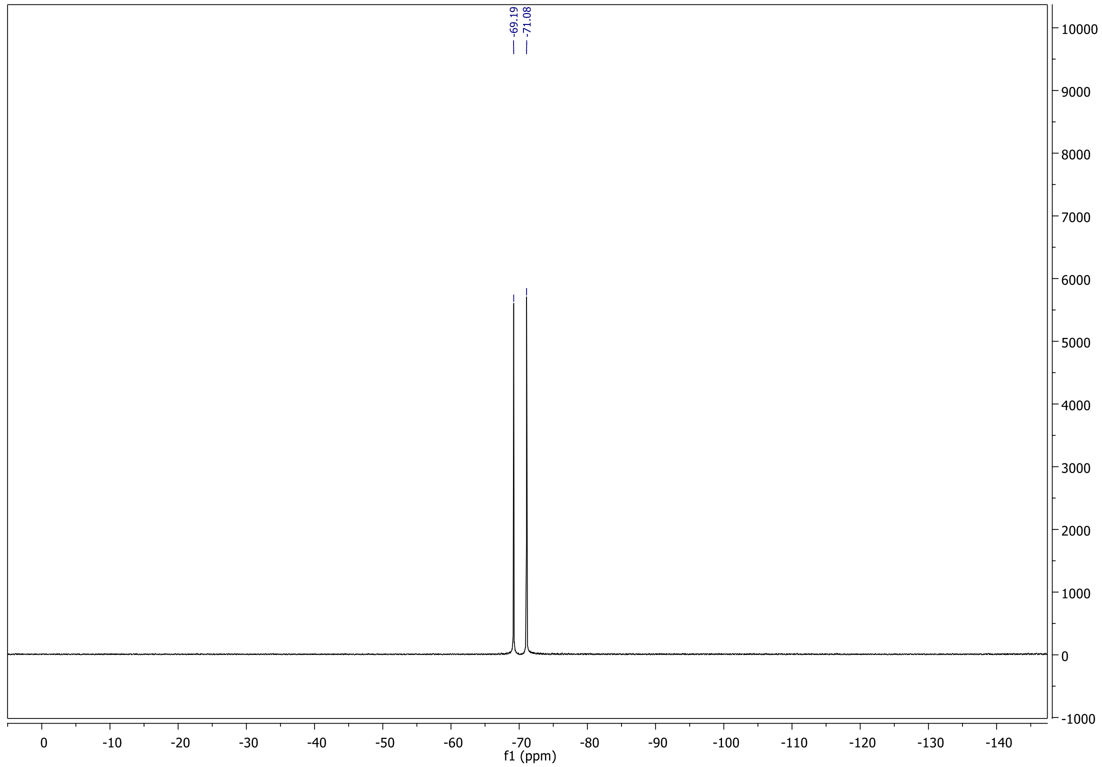

Figure S39. ^{19}F NMR of Compound **22**

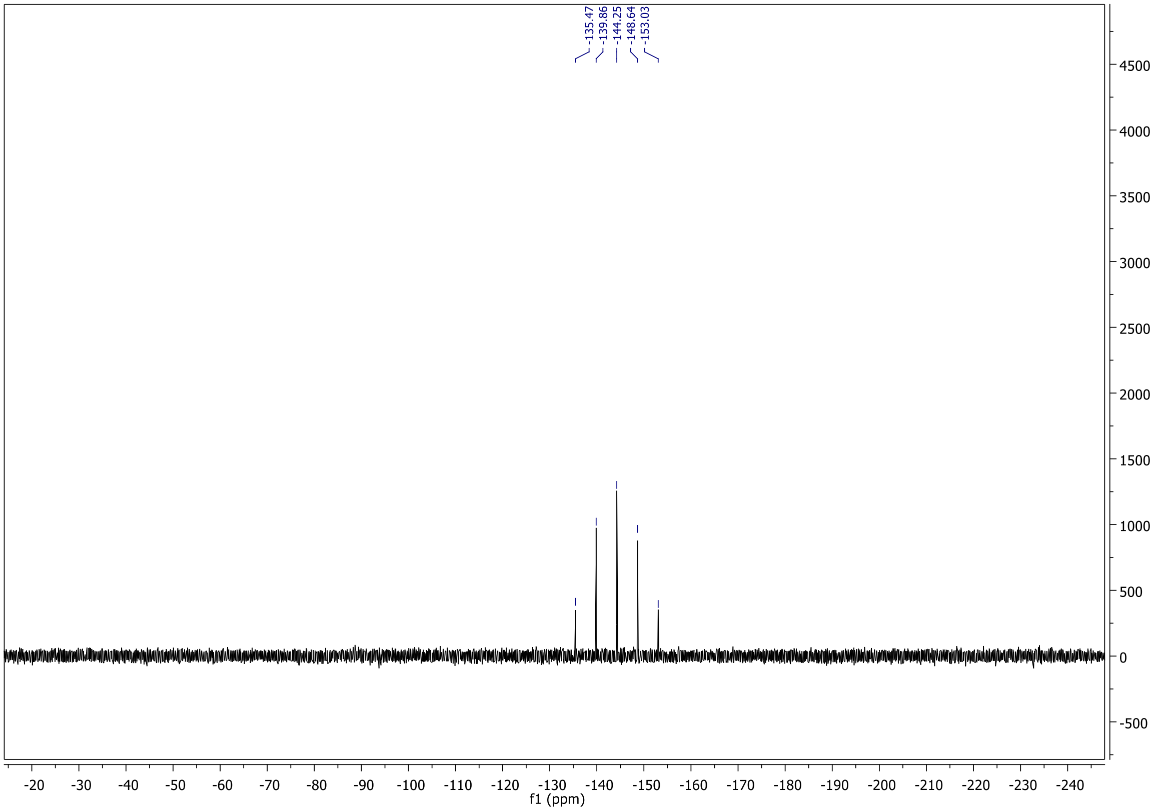

Figure S40. ^{31}P NMR of Compound **22**

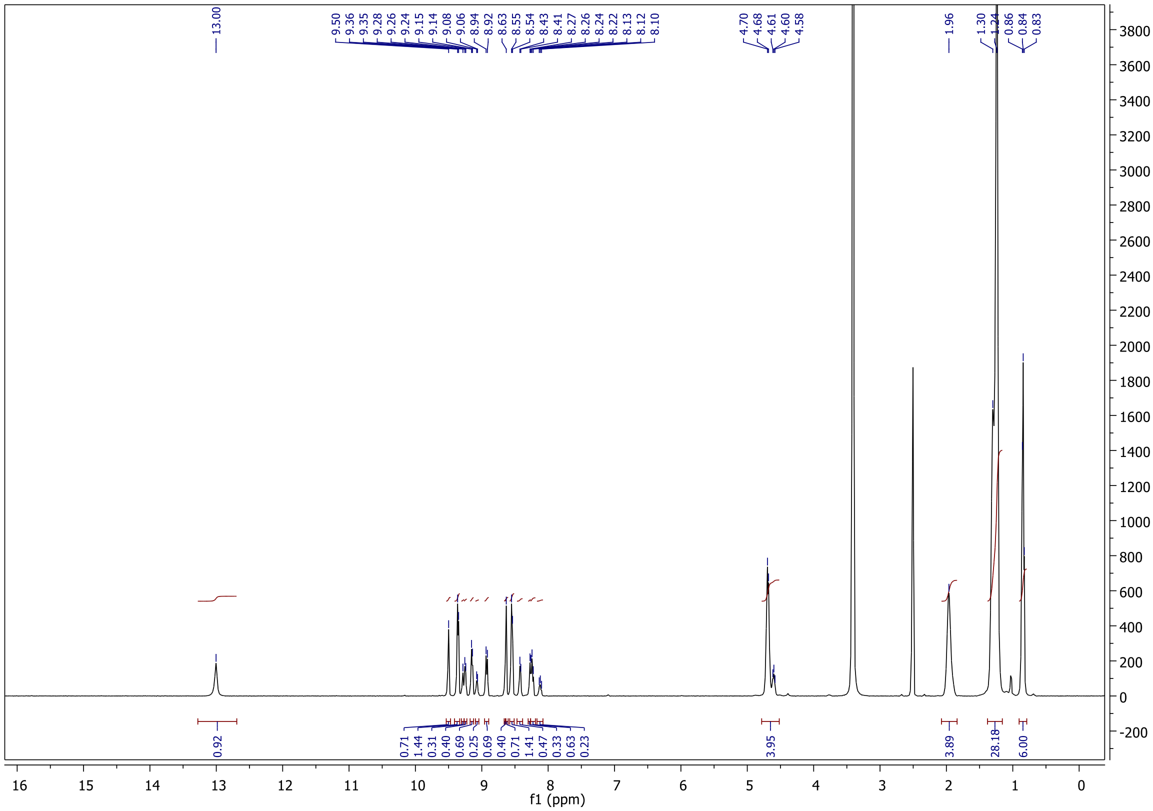

Figure S41. ^{1}H NMR of Compound **23**

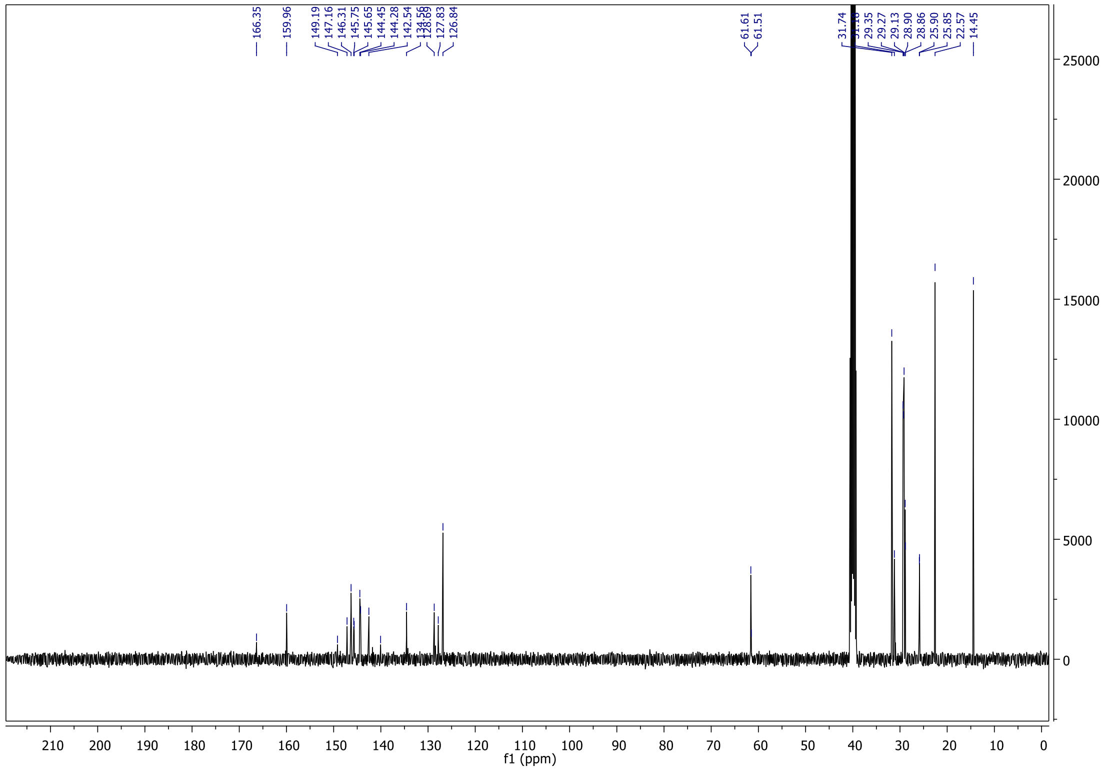

Figure S42. ^{13}C NMR of Compound **23**

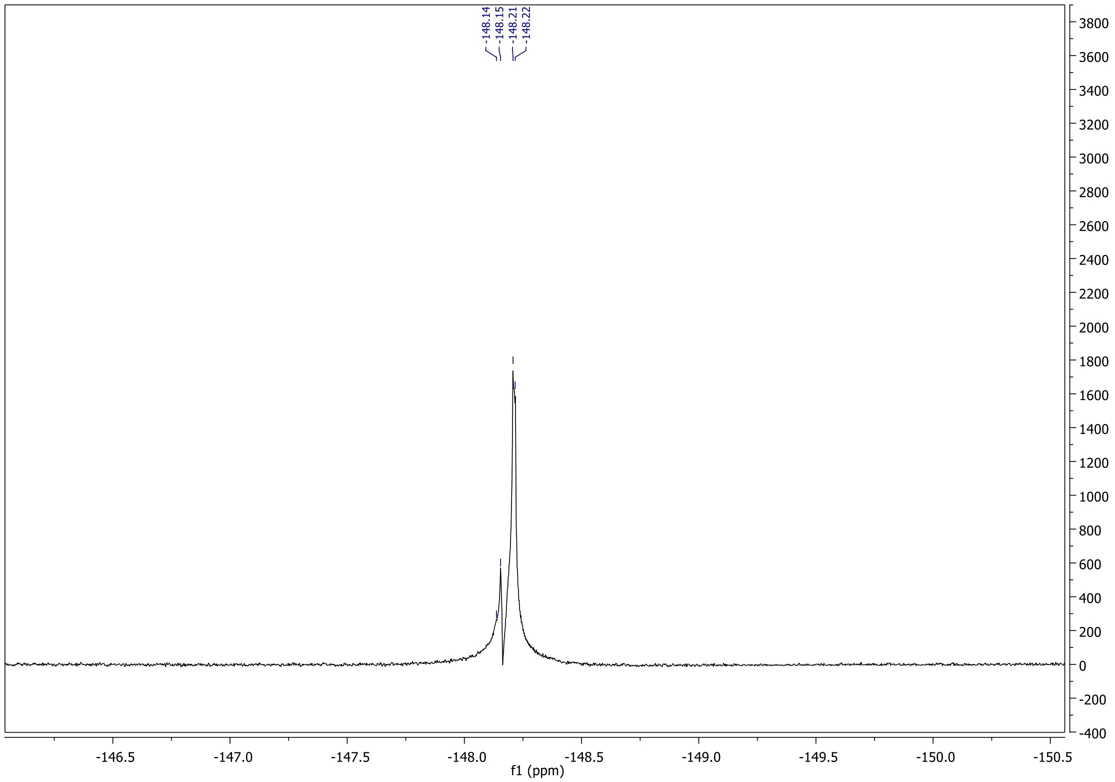

Figure S43. ^{19}F NMR of Compound **23**

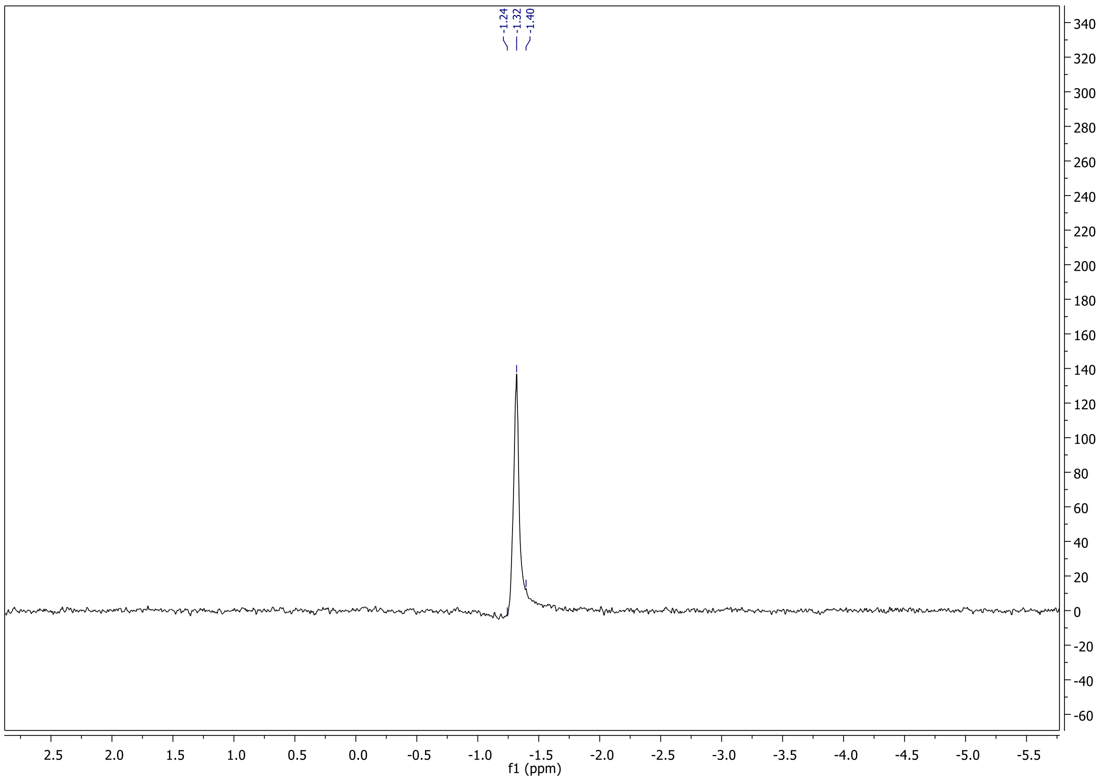

Figure S44. ^{11}B NMR of Compound **23**

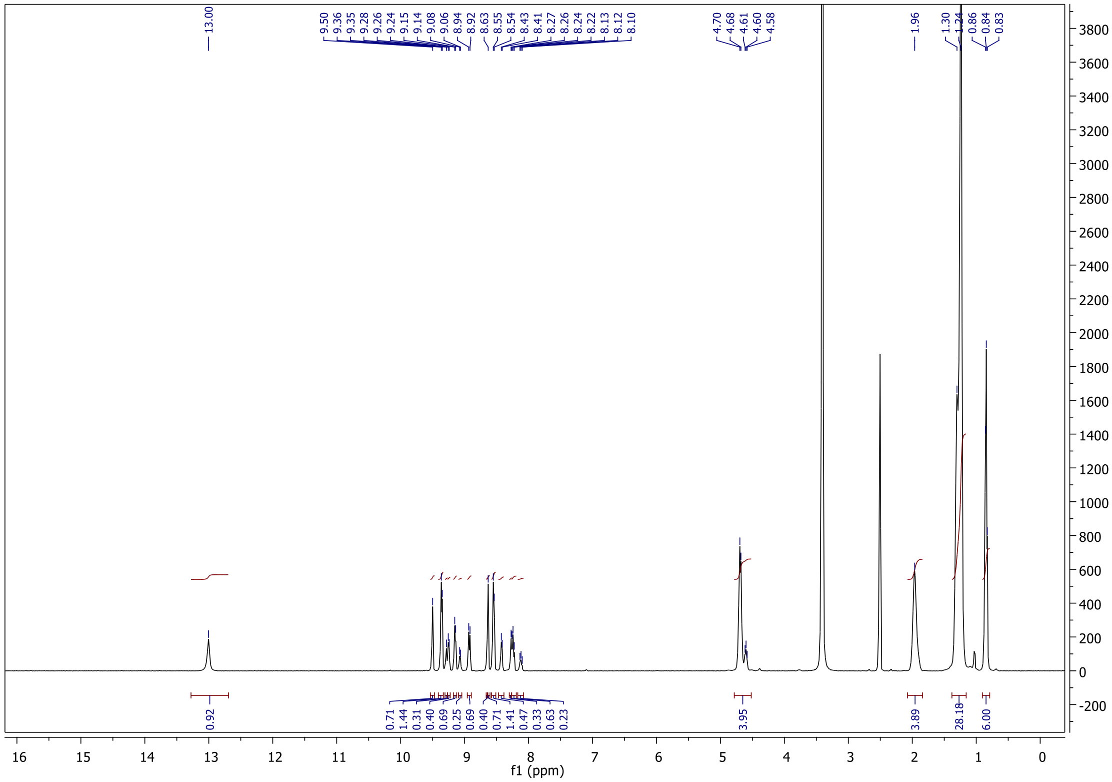

Figure S45. ^{1}H NMR of Compound **24**

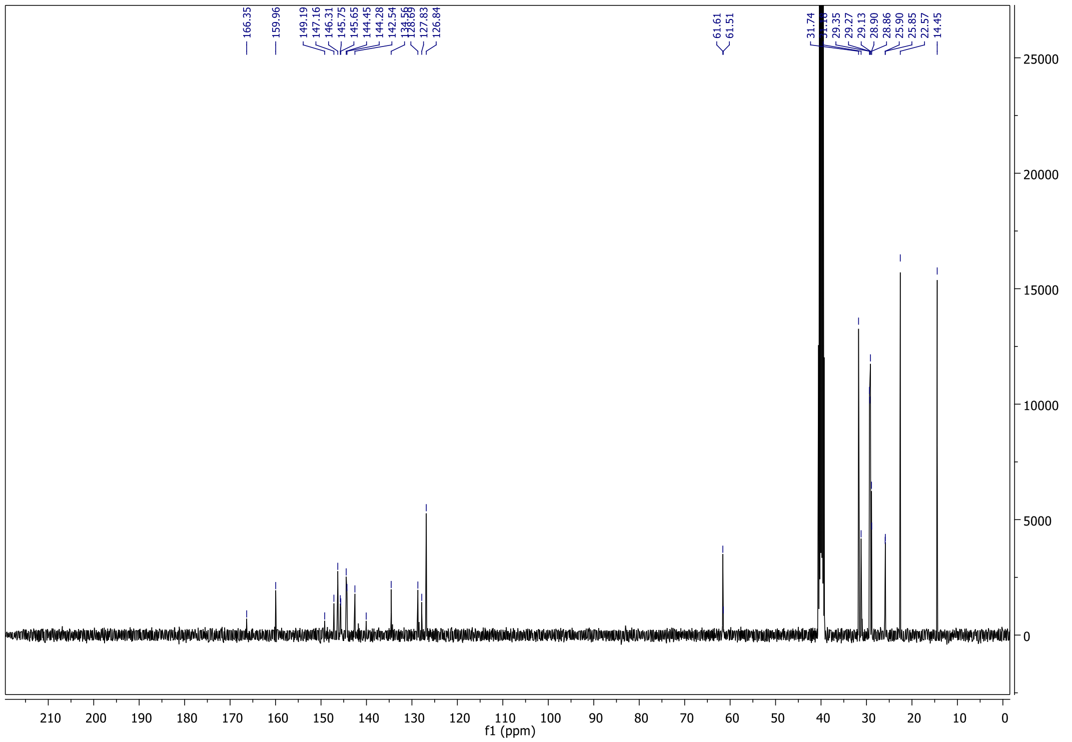

Figure S46. ^{13}C NMR of Compound **24**

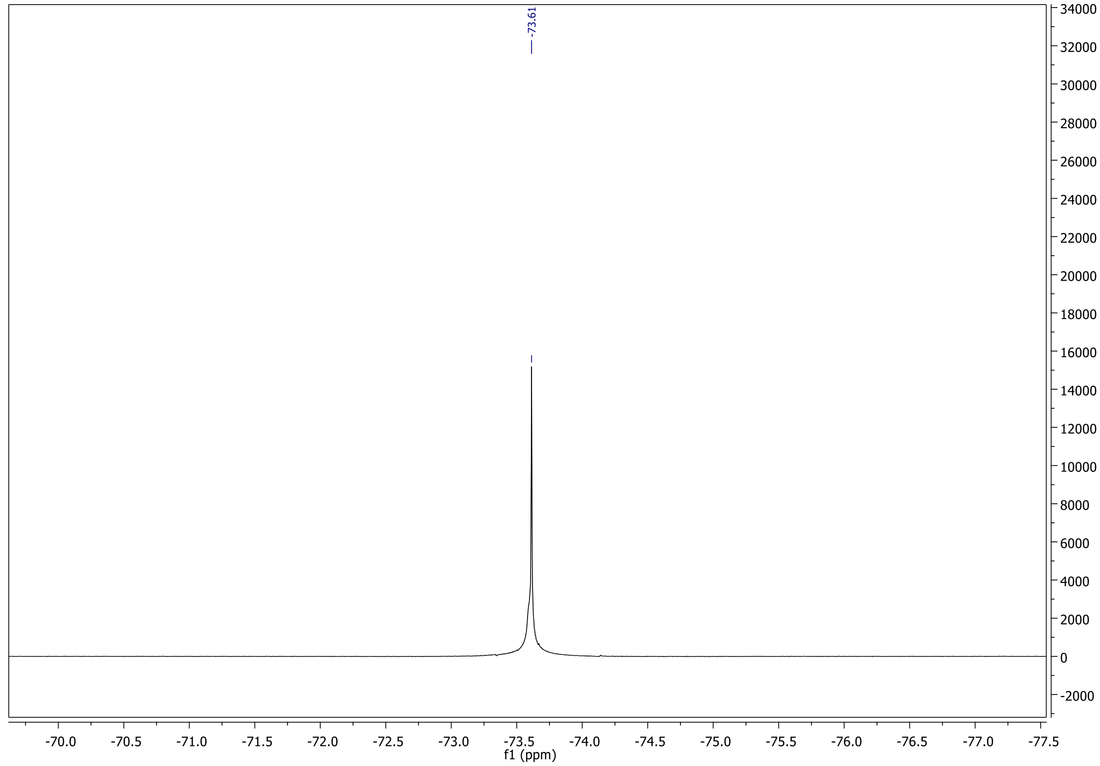

Figure S47. ^{19}F NMR of Compound **24**

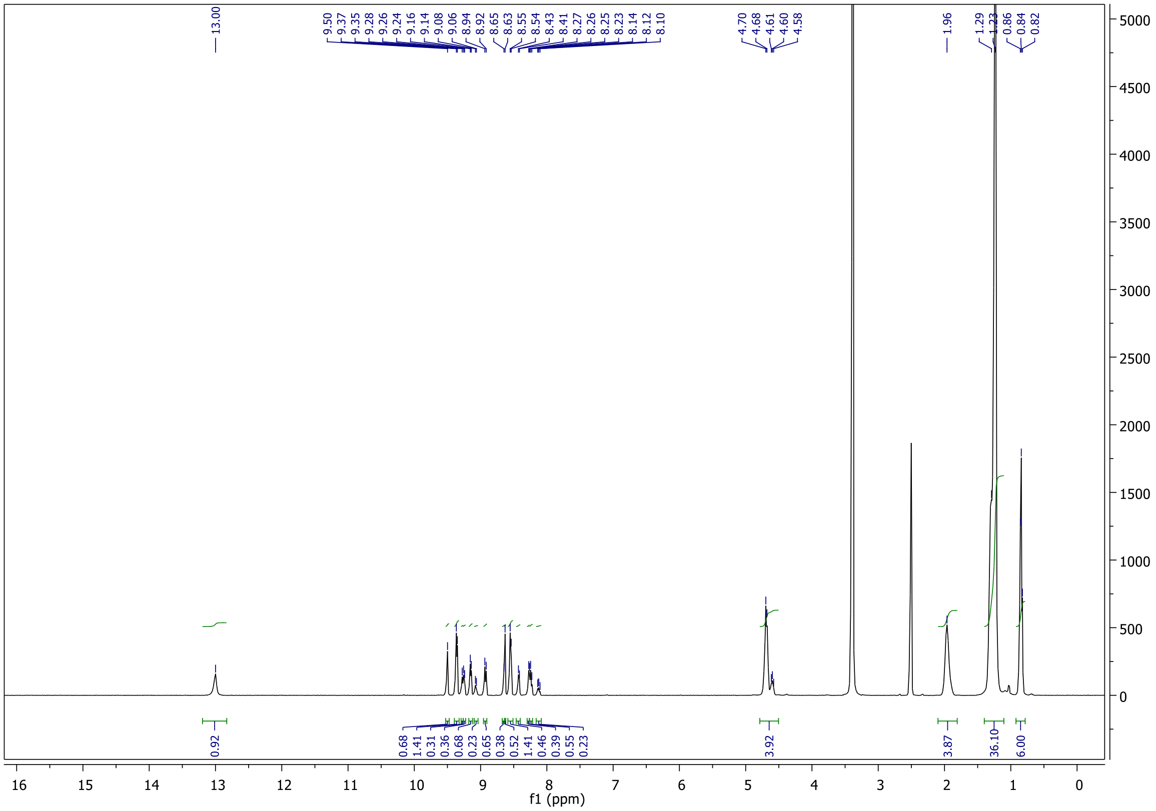

Figure S48. ^{1}H NMR of Compound **25**

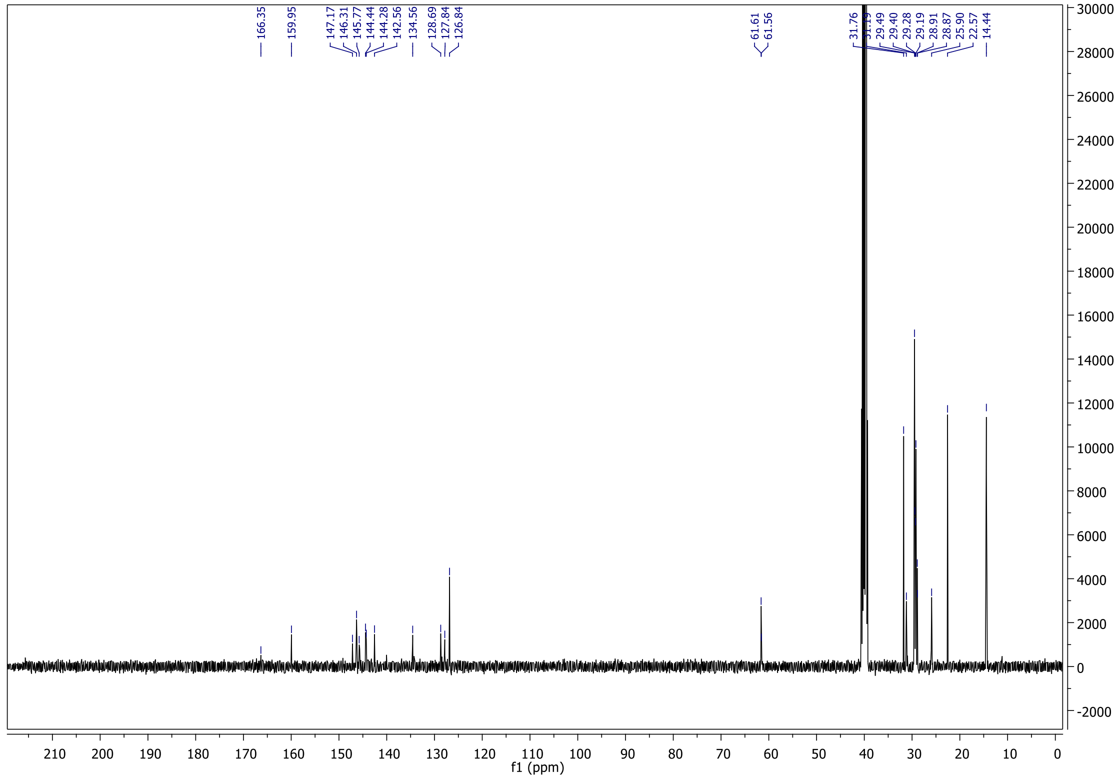

Figure S49. ^{13}C NMR of Compound **25**

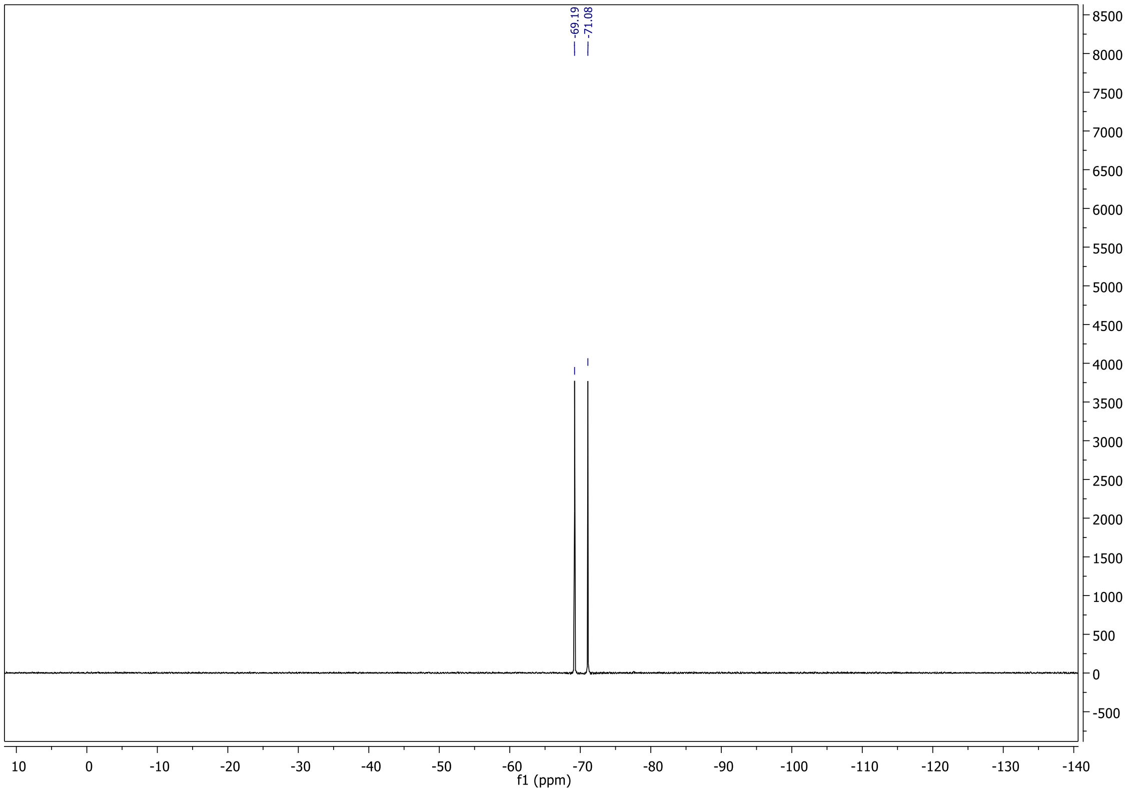

Figure S50. ^{19}F NMR of Compound **25**

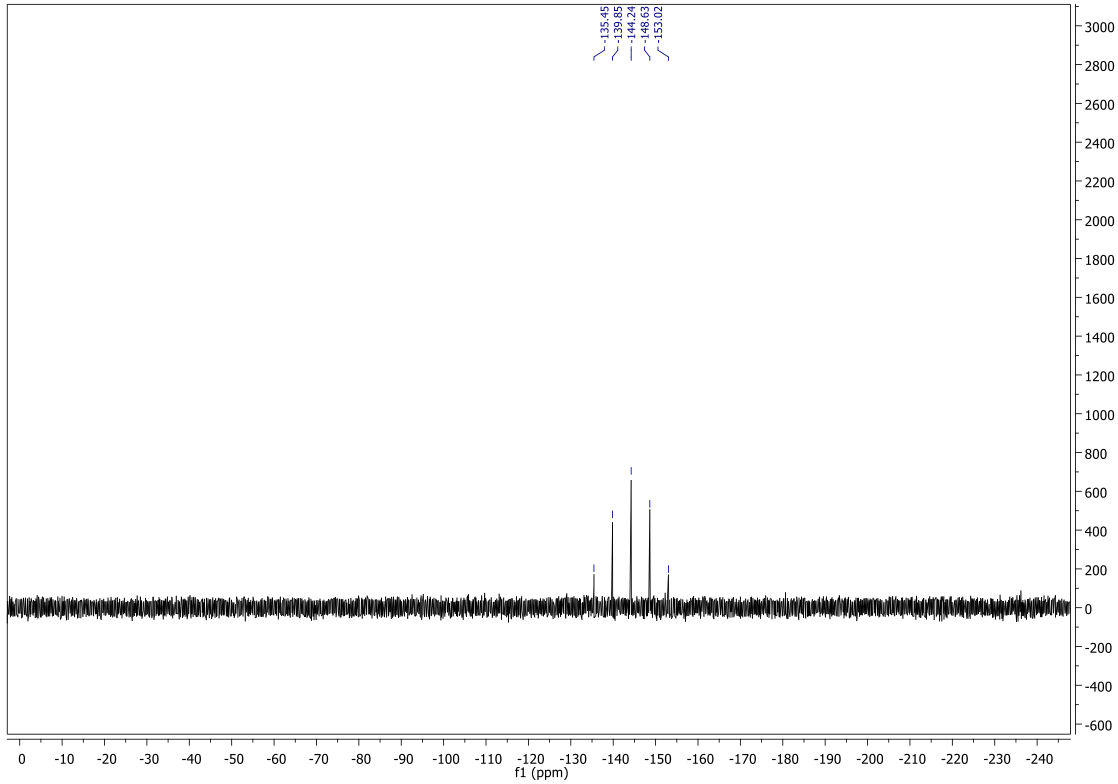

Figure S51. ^{31}P NMR of Compound **25**

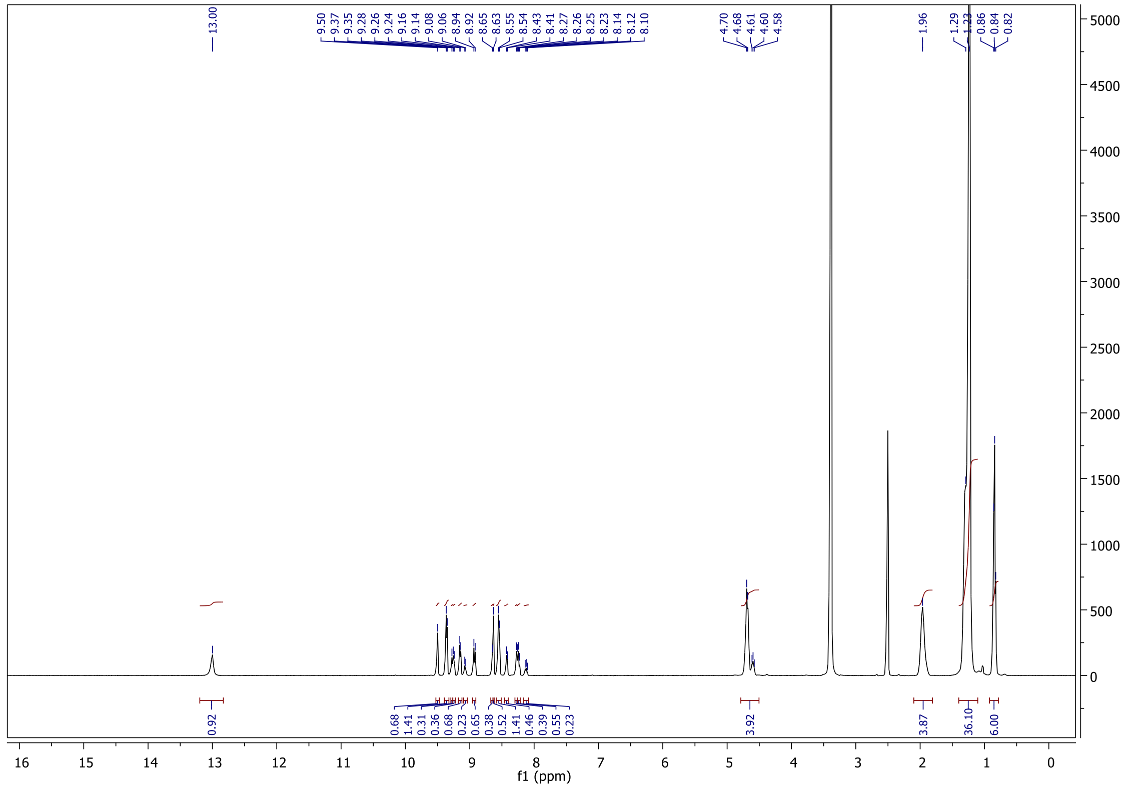

Figure S52. ^{1}H NMR of Compound **26**

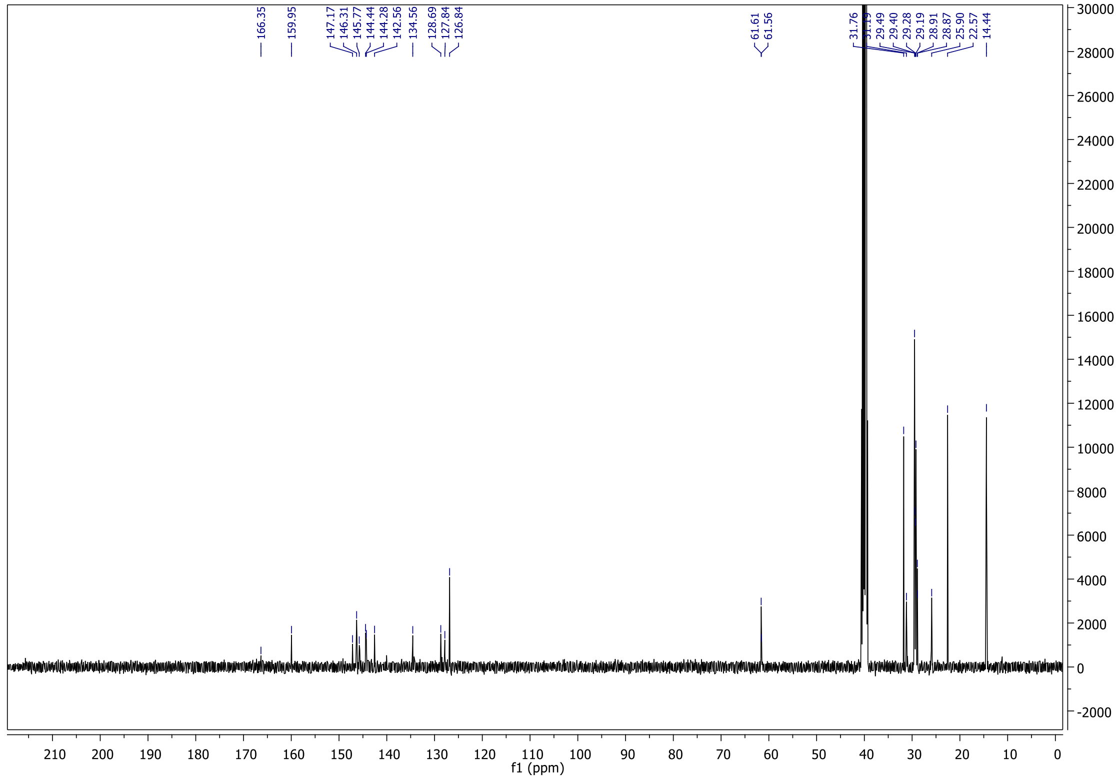

Figure S53. ^{13}C NMR of Compound **26**

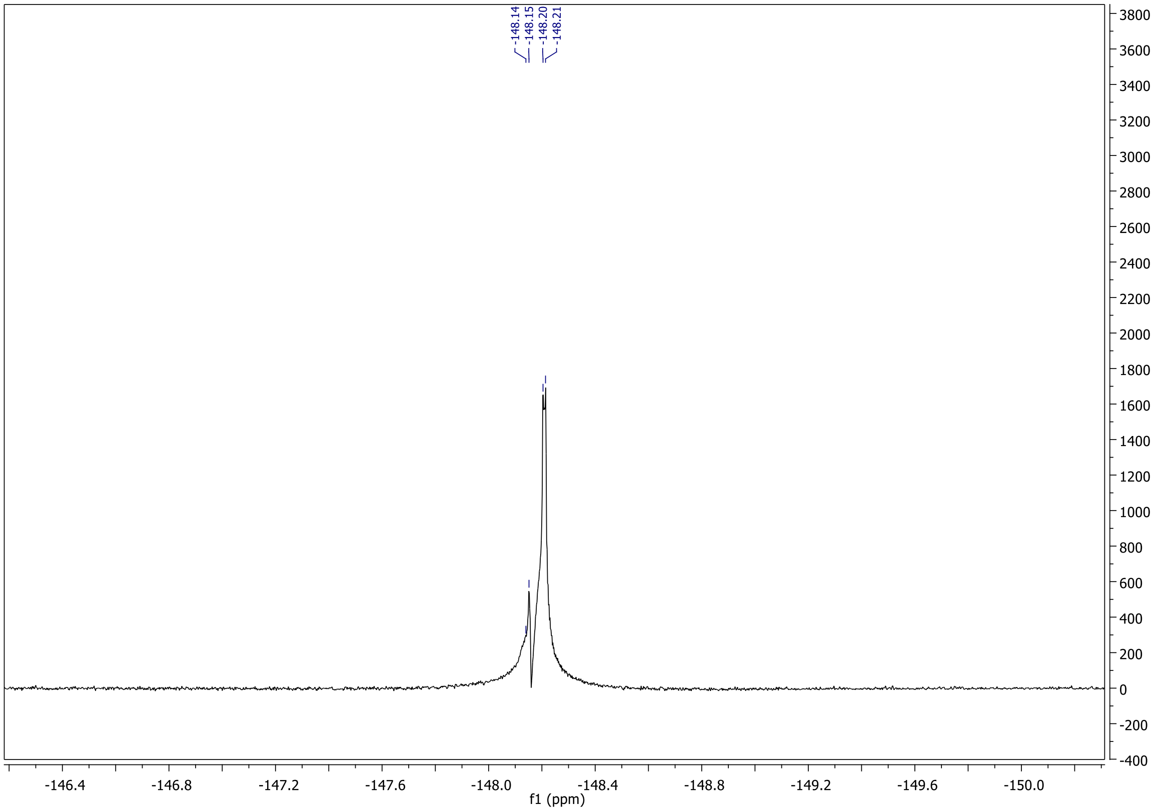

Figure S54. ^{19}F NMR of Compound **26**

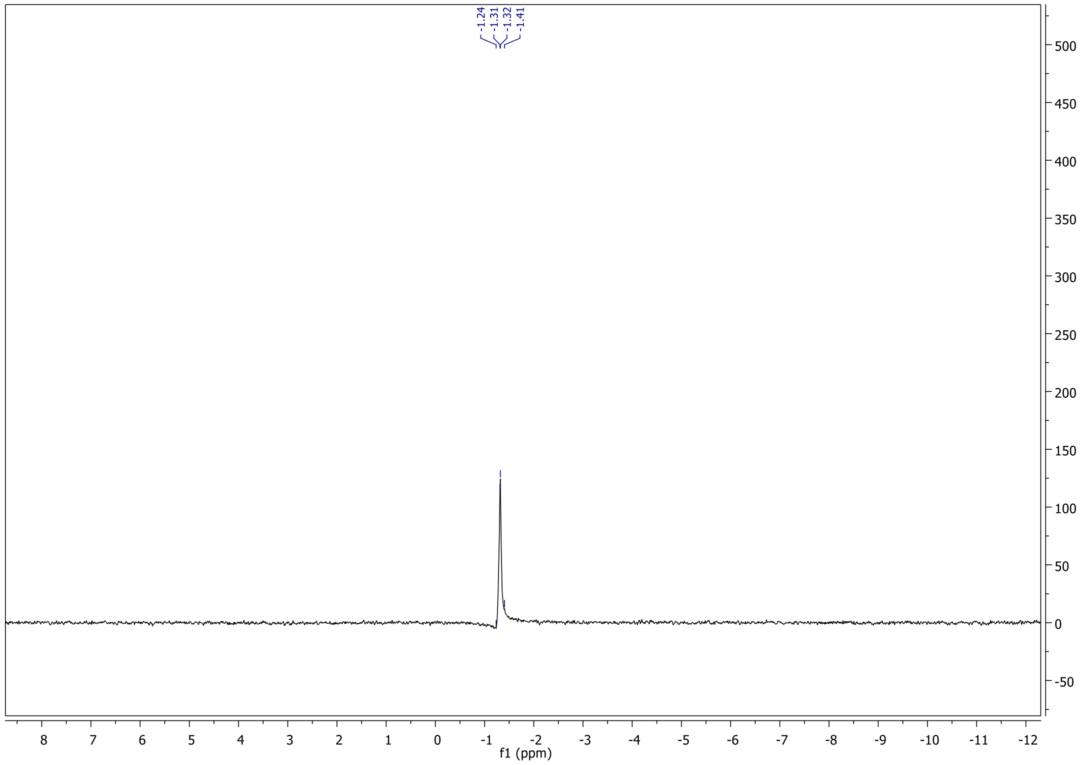

Figure S55. ^{11}B NMR of Compound **26**

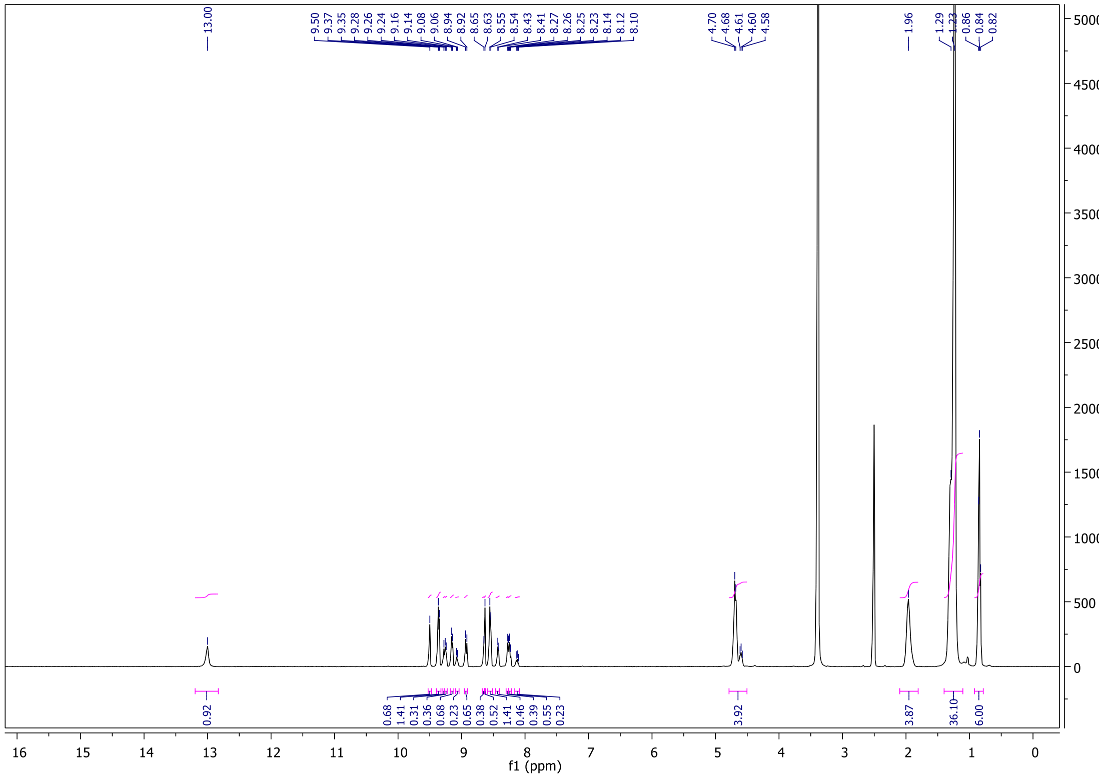

Figure S56. ^{1}H NMR of Compound **27**

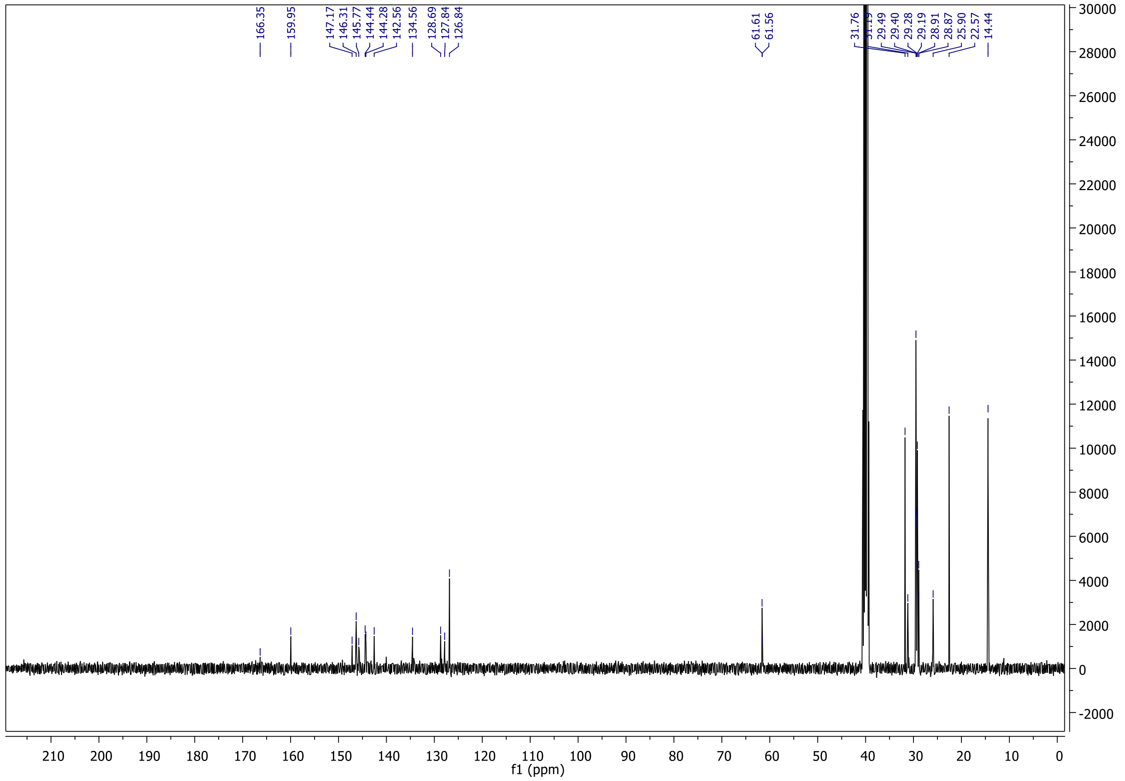

Figure S57. ^{13}C NMR of Compound **27**

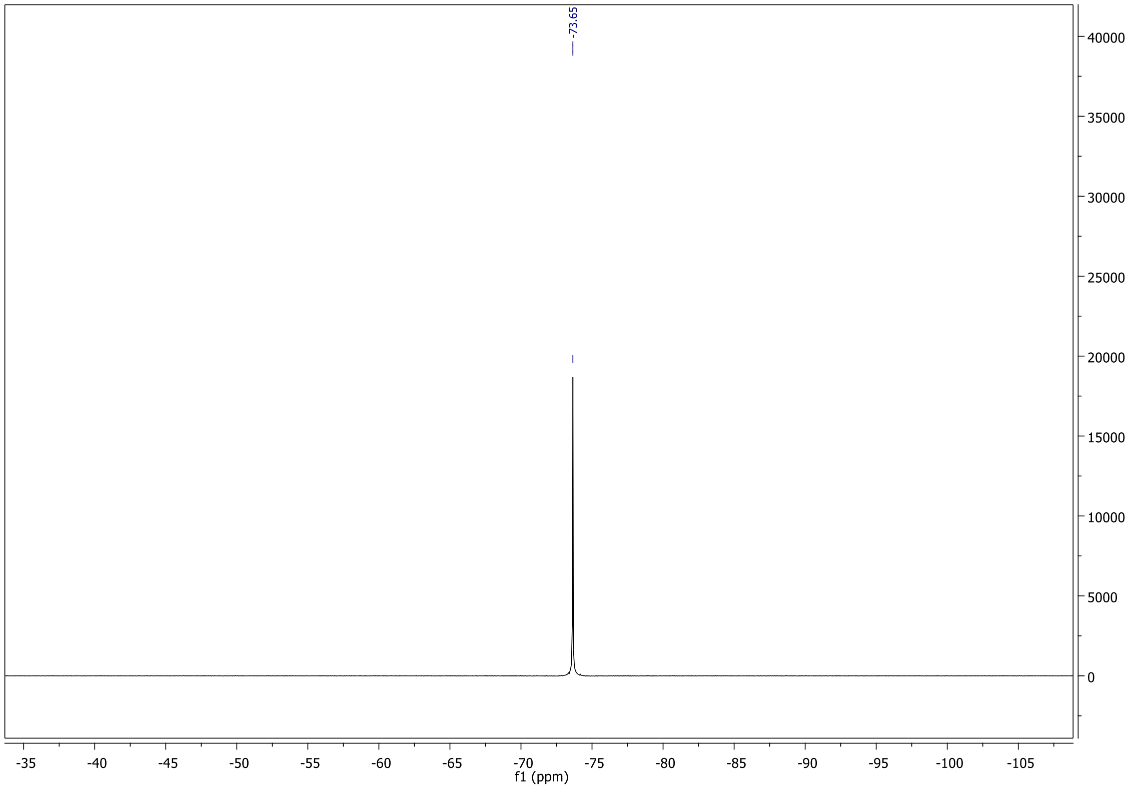

Figure S58. ^{19}F NMR of Compound **27**

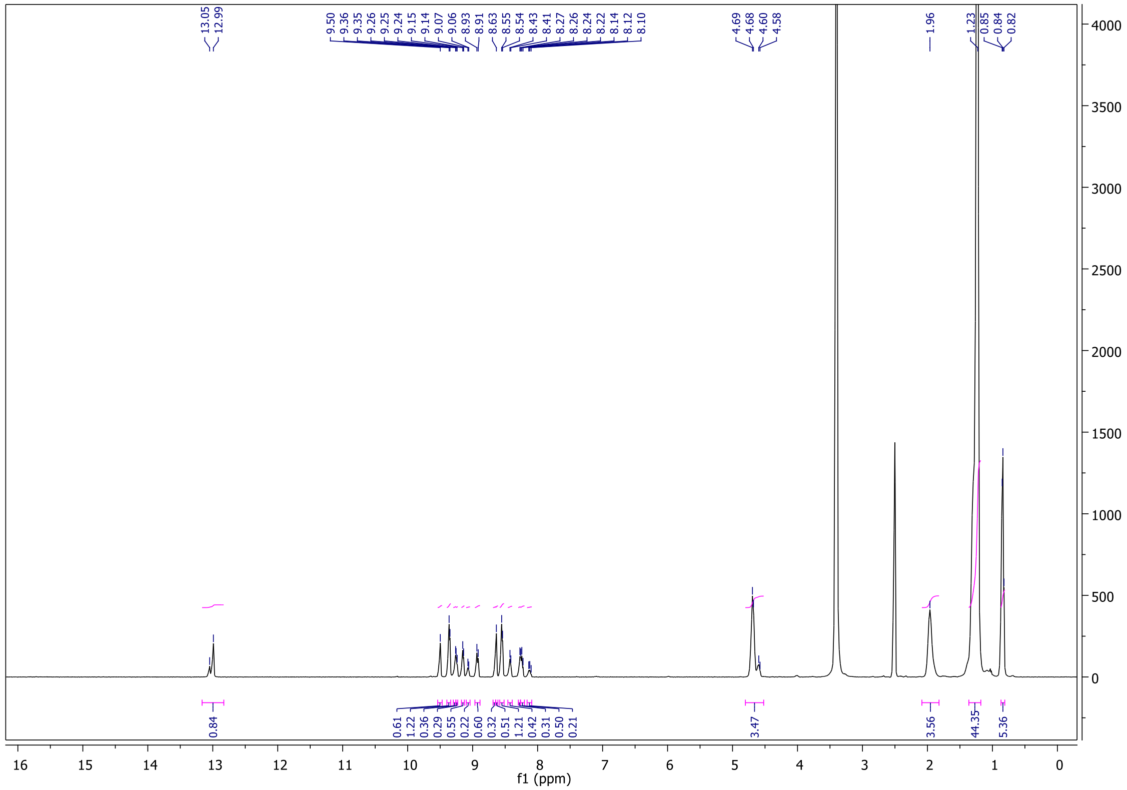

Figure S59. ^{1}H NMR of Compound **28**

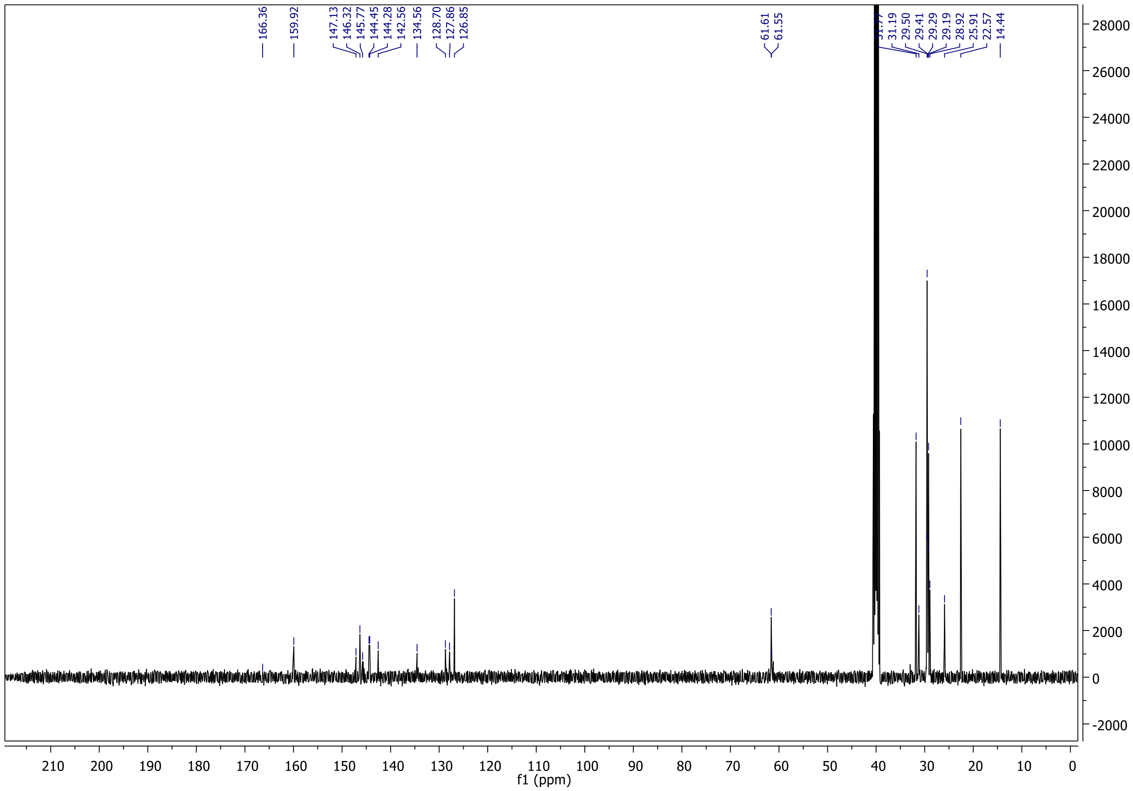

Figure S60 ^{13}C NMR of Compound **28**

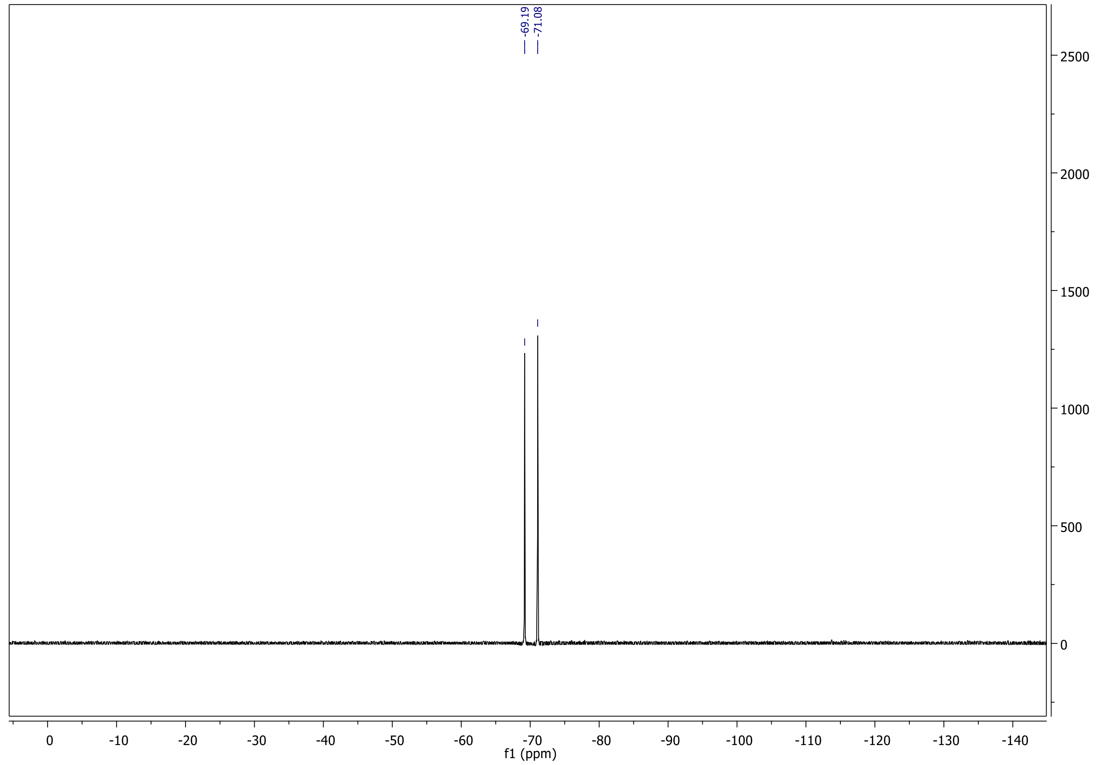

Figure S61. ^{19}F NMR of Compound **28**

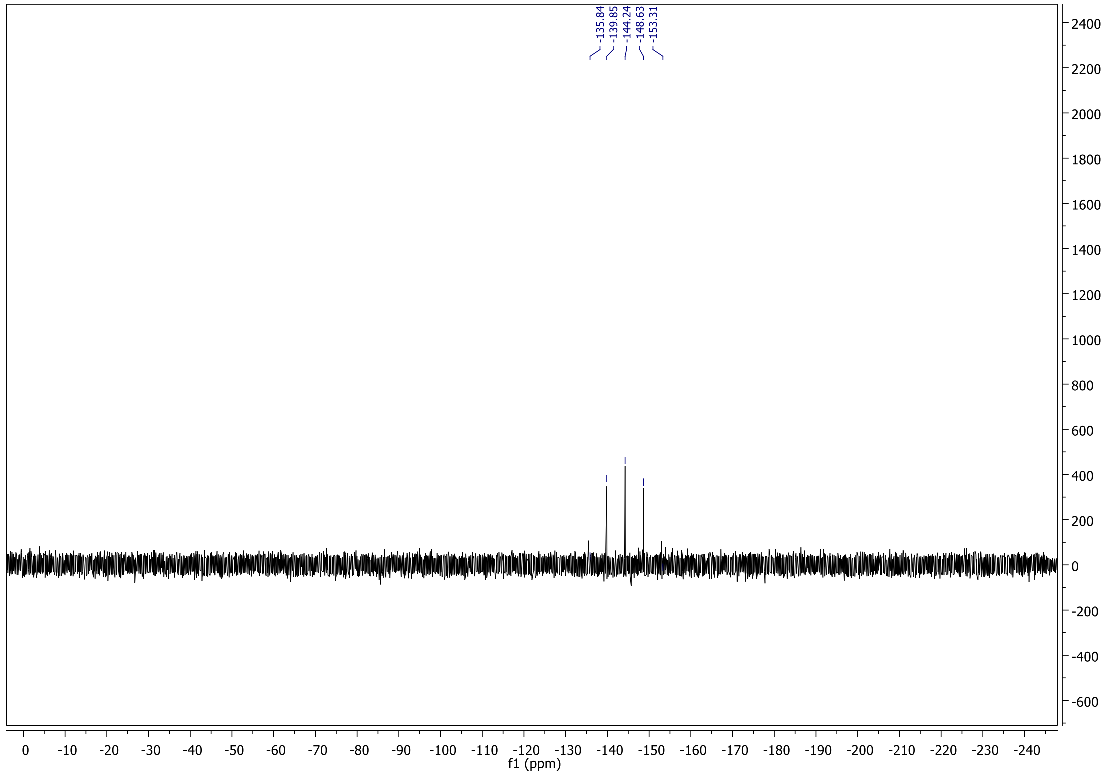

Figure S62. ^{31}P NMR of Compound **28**

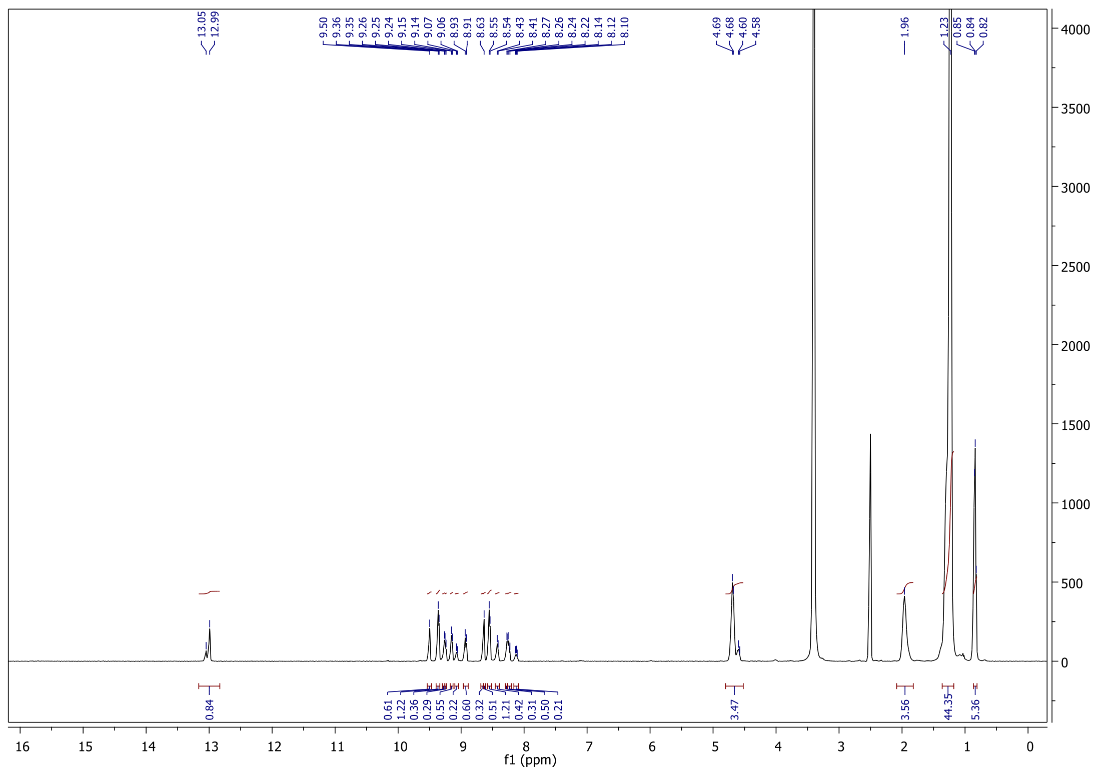

Figure S63. ^{1}H NMR of Compound **29**

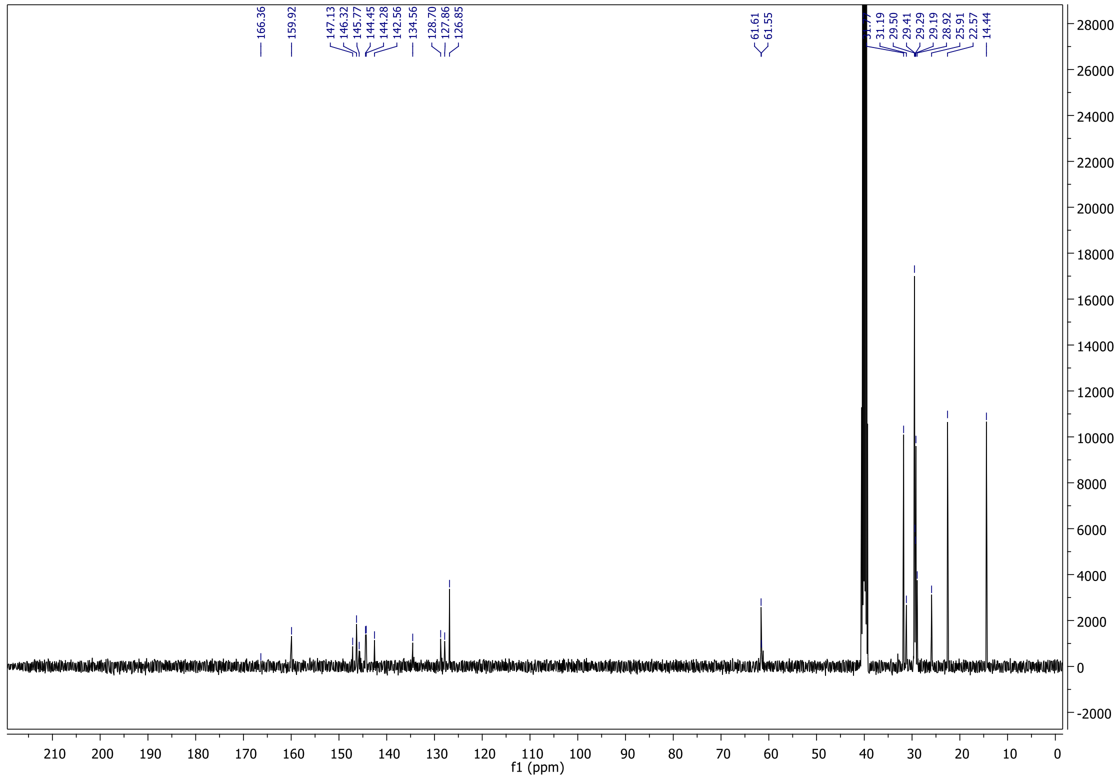

Figure S64. ^{13}C NMR of Compound **29**

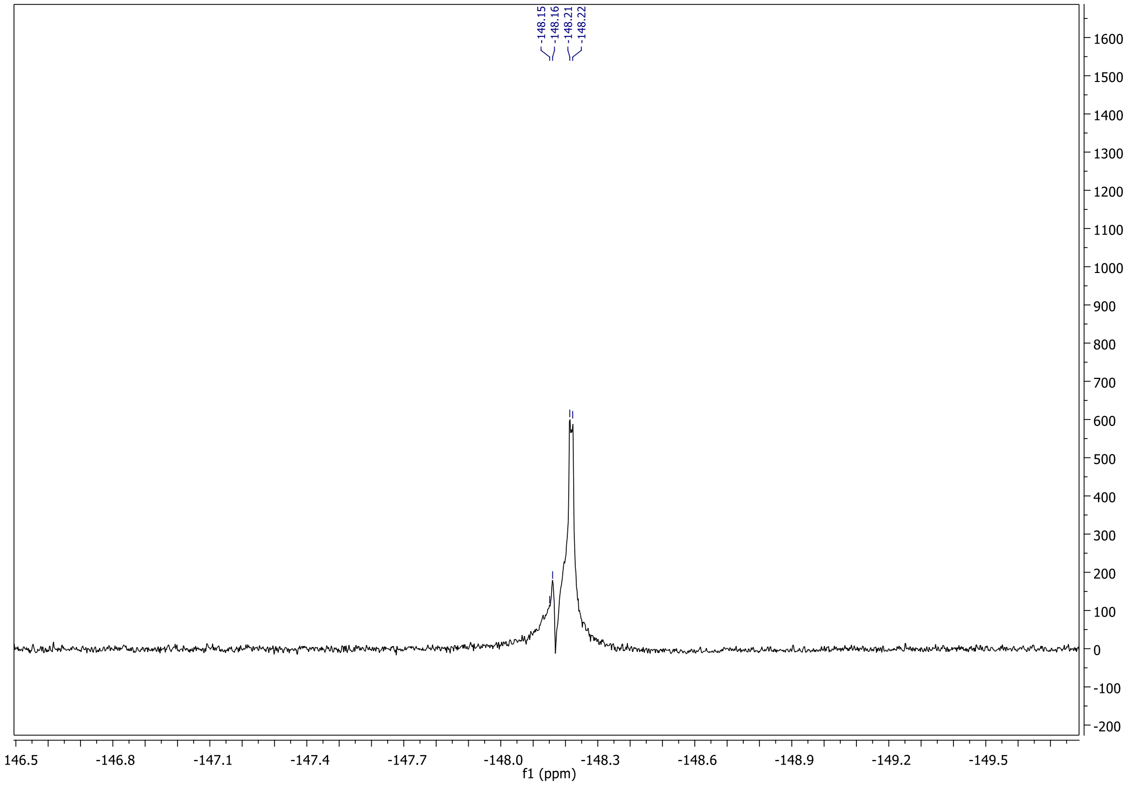

Figure S65. ^{19}F NMR of Compound **29**

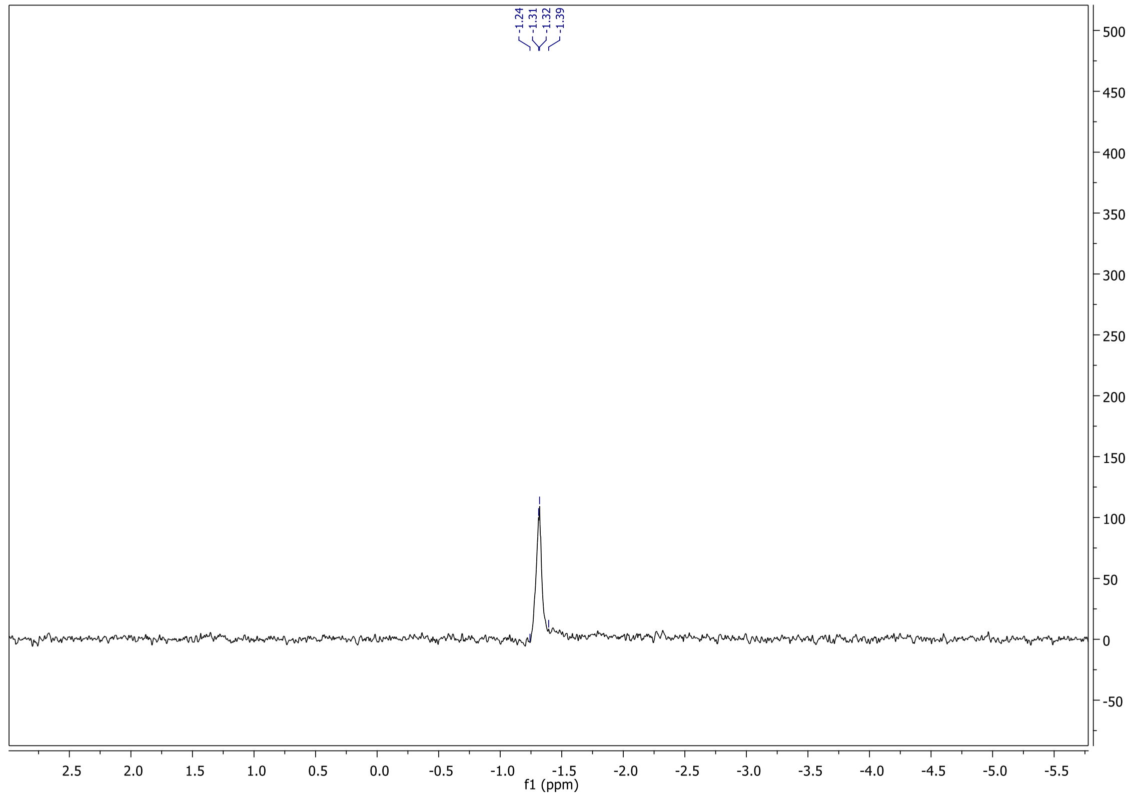

Figure S66. ^{11}B NMR of Compound **29**

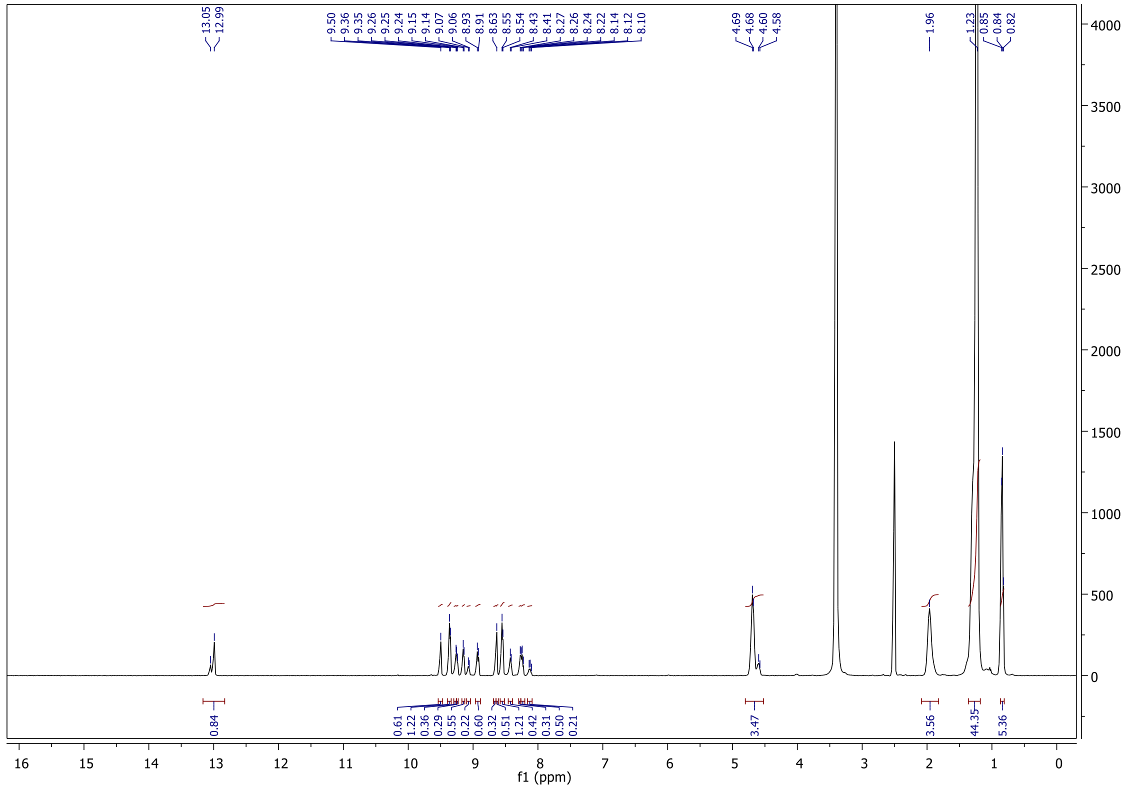

Figure S67. ^{1}H NMR of Compound **30**

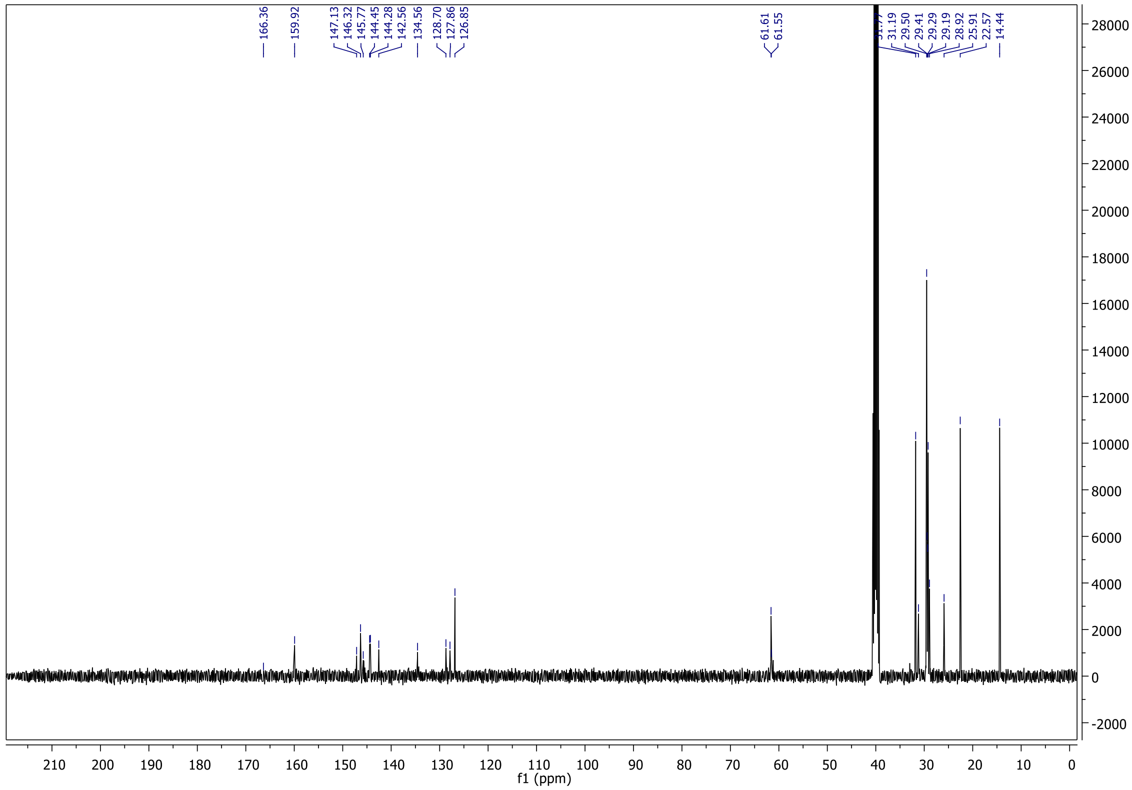

Figure S68. ^{13}C NMR of Compound **30**

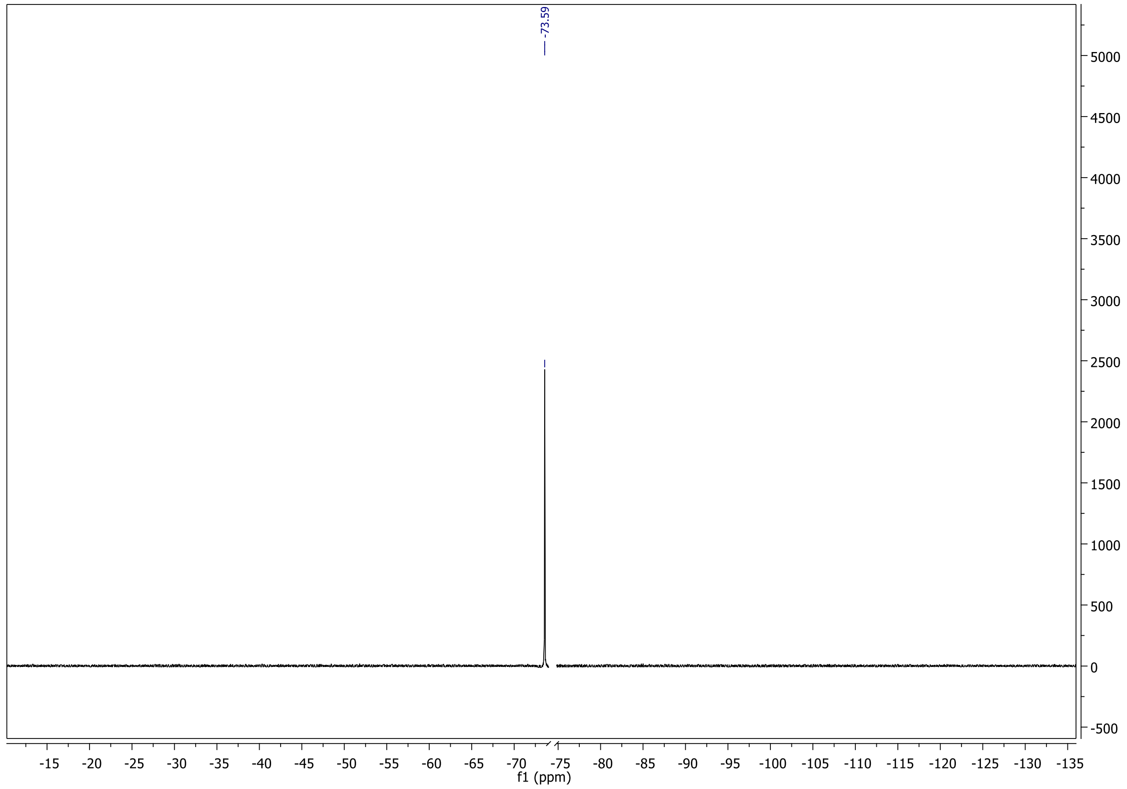

Figure S69. ^{19}F NMR of Compound **30**

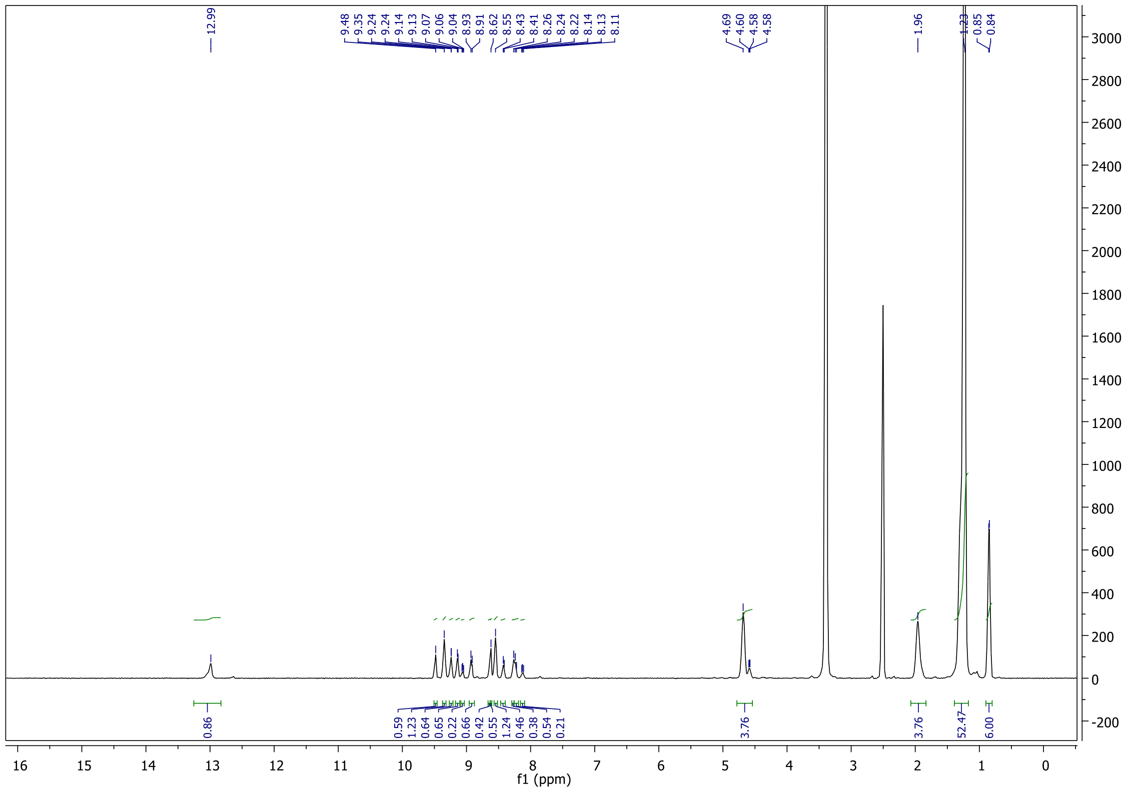

Figure S70. ^{1}H NMR of Compound **31**

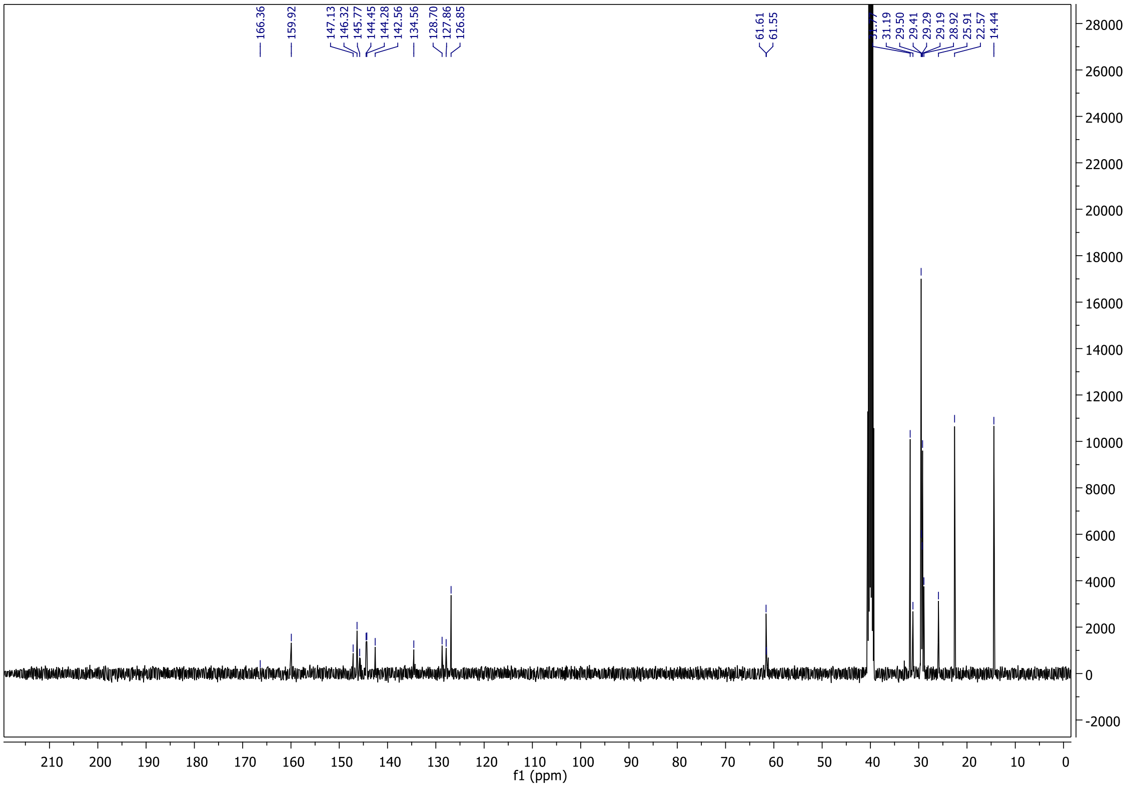

Figure S71. ^{13}C NMR of Compound **31**

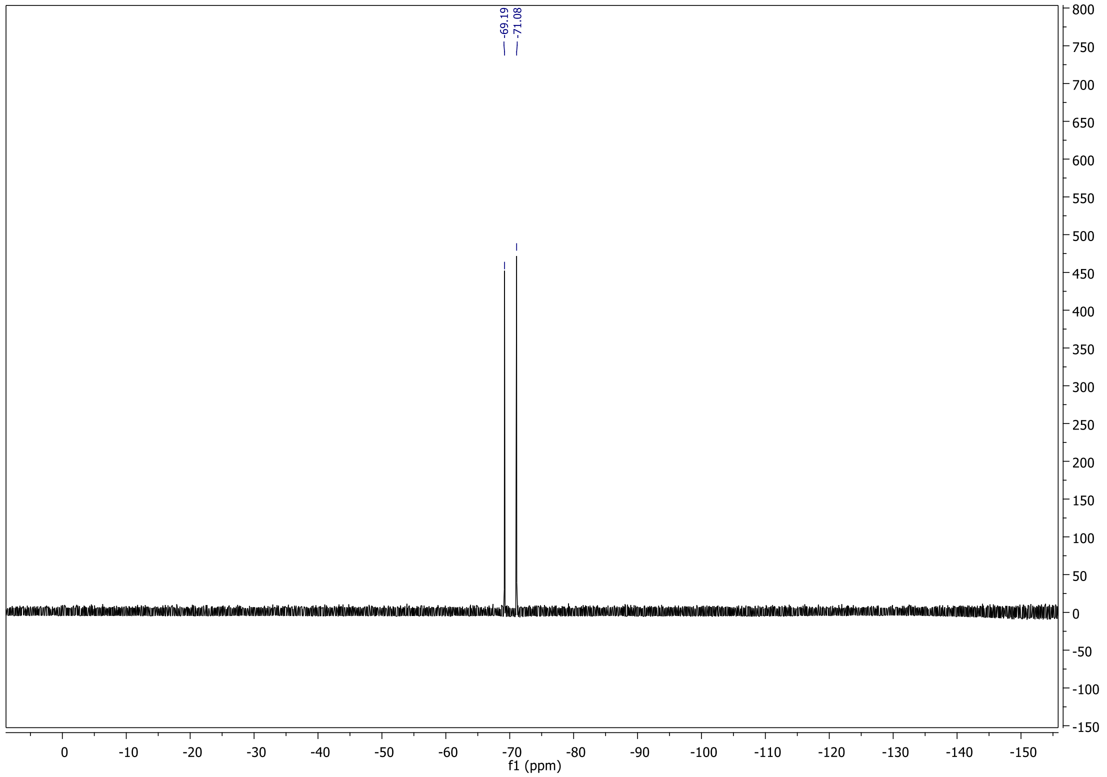

Figure S72. ^{19}F NMR of Compound **31**

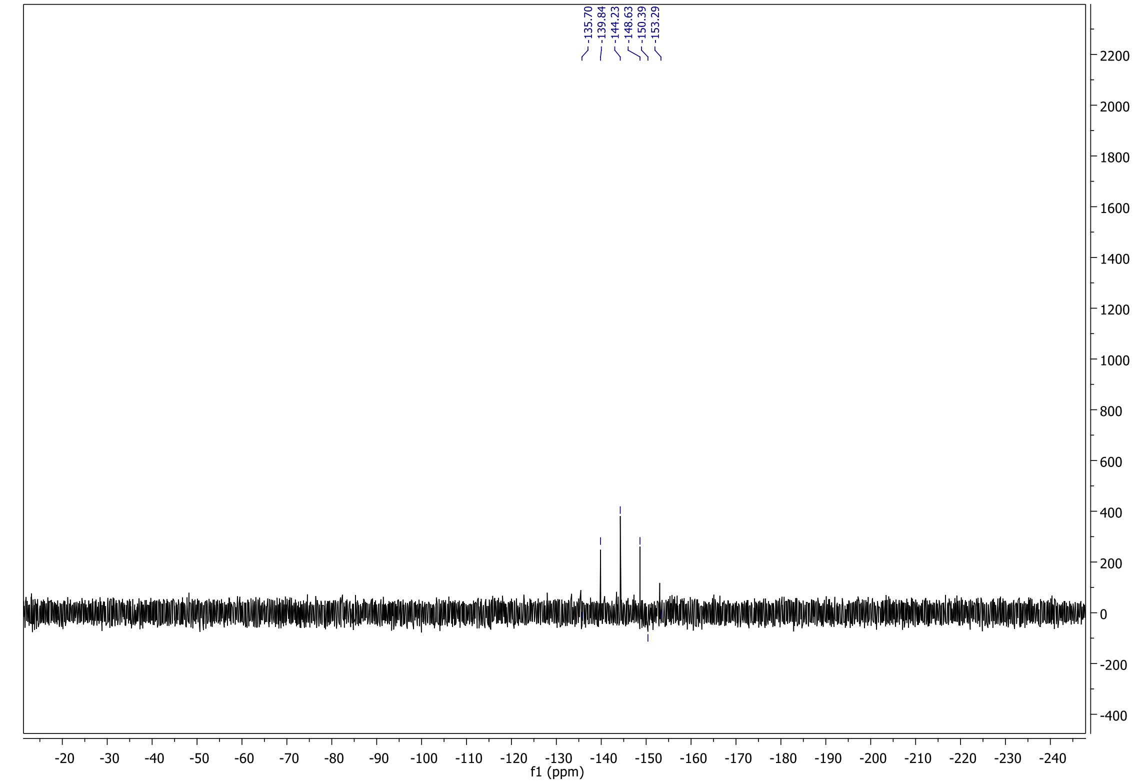

Figure S73. ³¹P NMR of Compound **31**

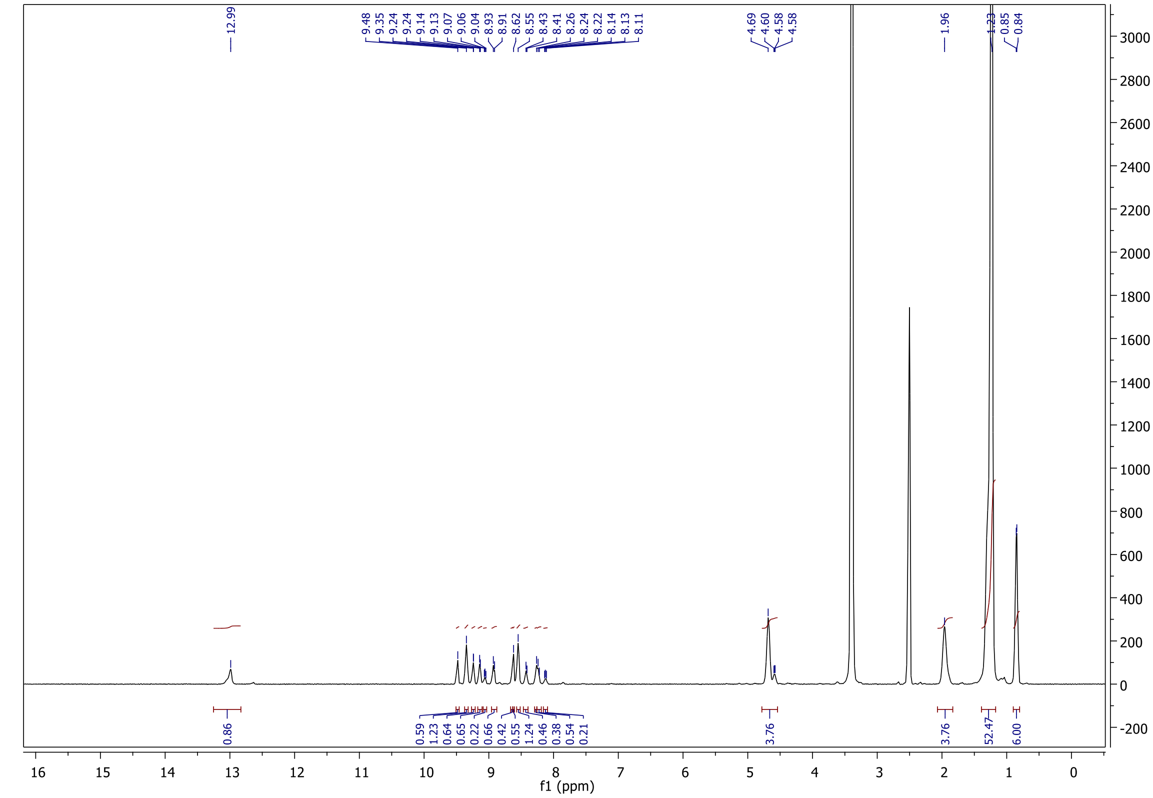

Figure S74. ¹H NMR of Compound **32**

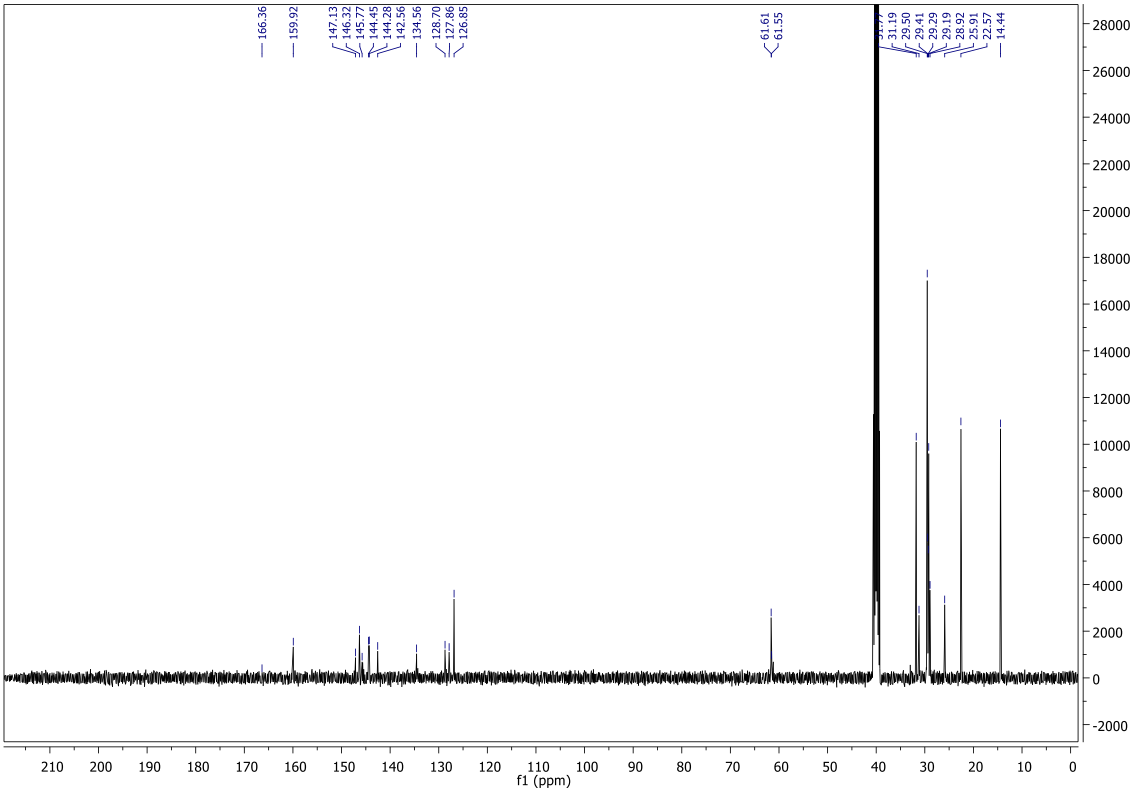

Figure S75. ^{13}C NMR of Compound **32**

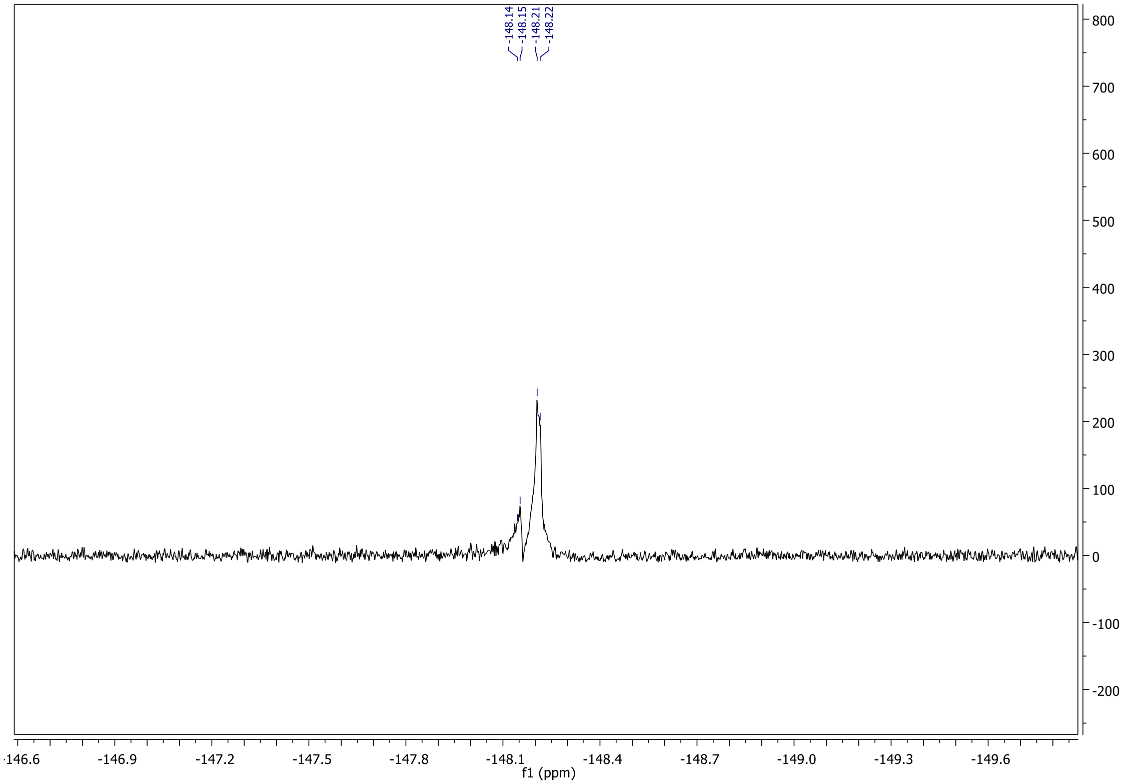

Figure S76. ^{19}F NMR of Compound **32**

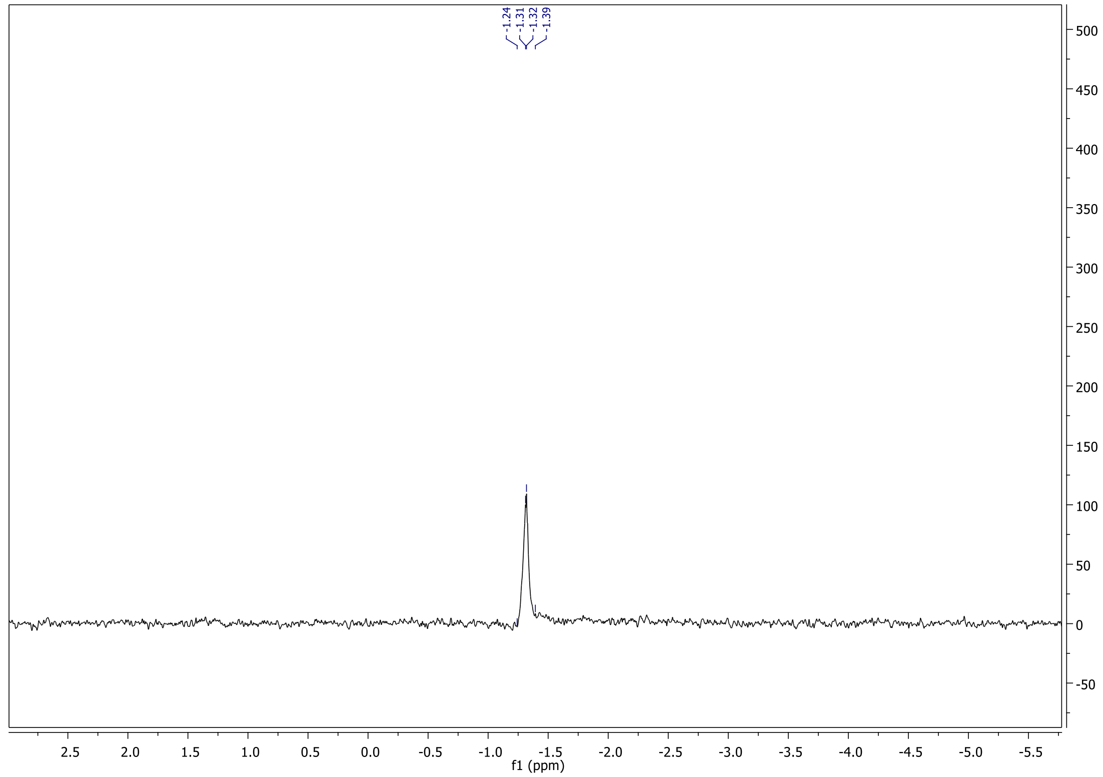

Figure S77. ^{11}B NMR of Compound **32**

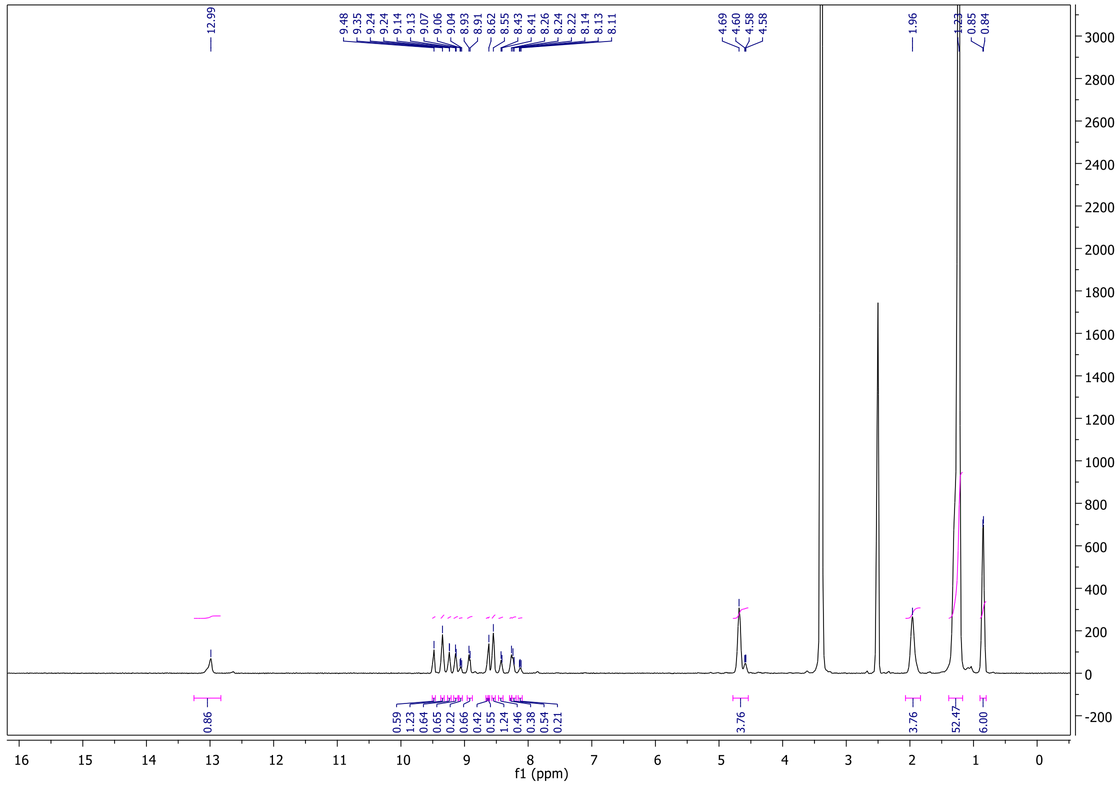

Figure S78. ^{1}H NMR of Compound **33**

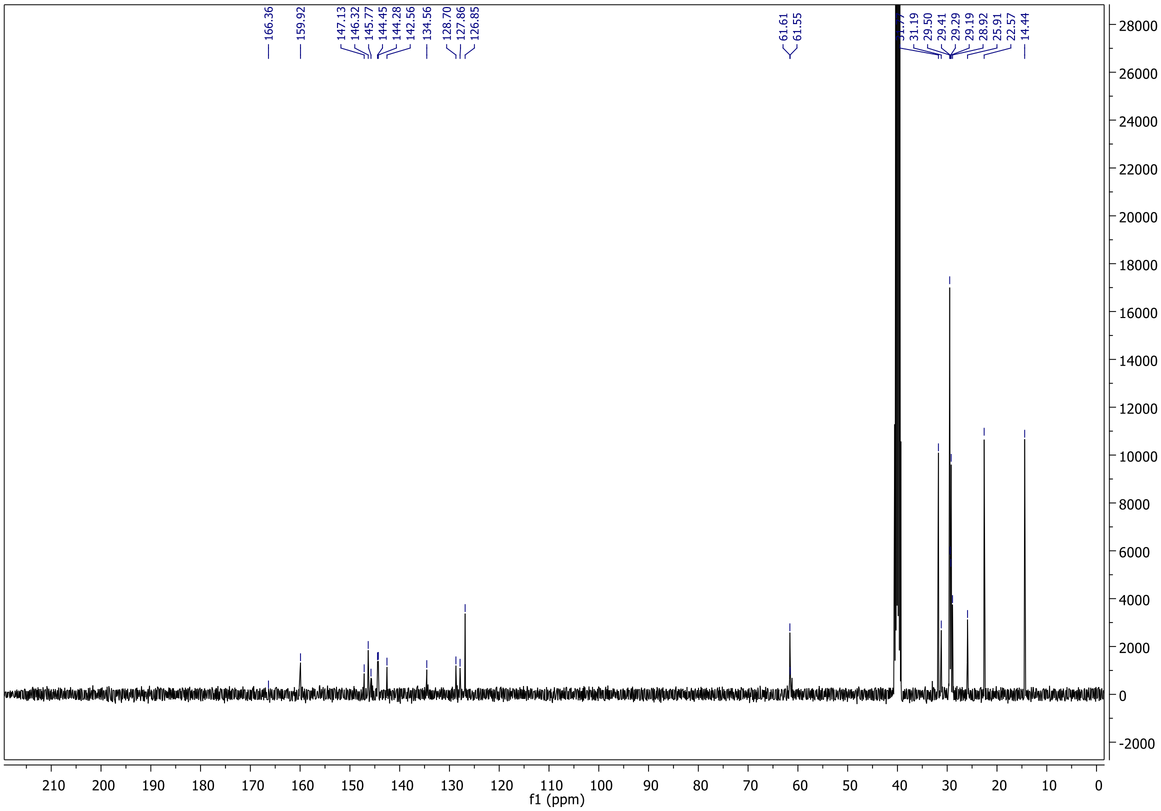

Figure S79. ^{13}C NMR of Compound **33**

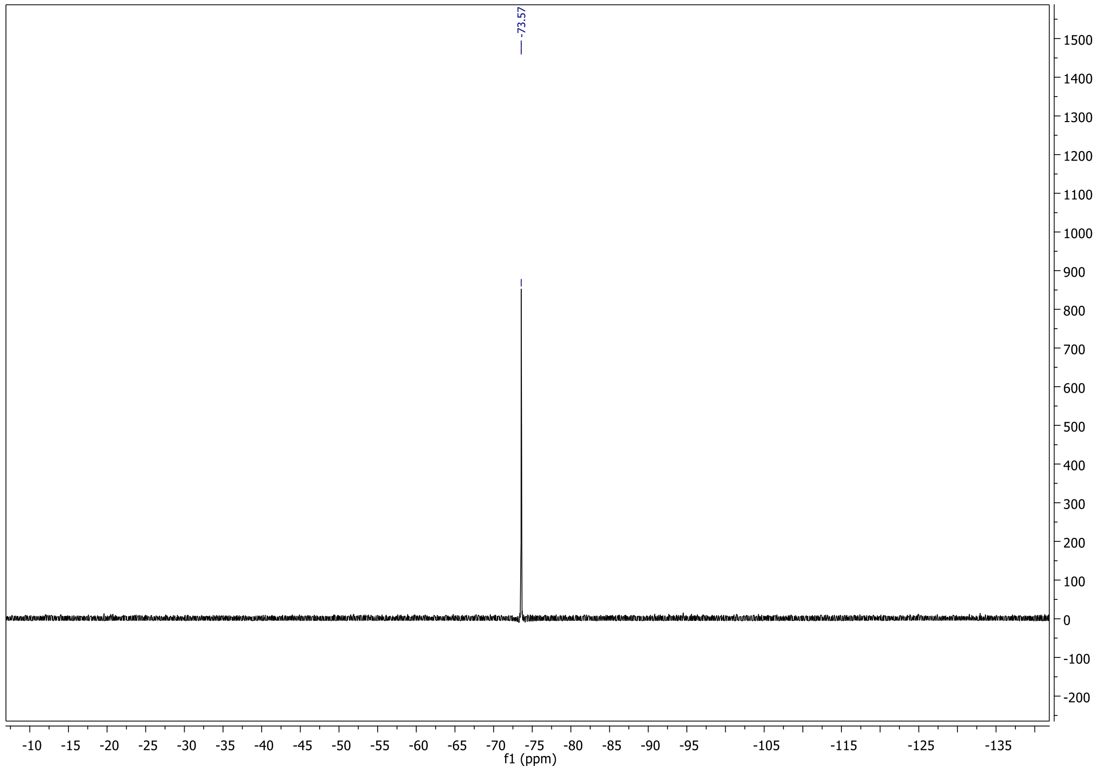

Figure S80. ^{19}F NMR of Compound **33**

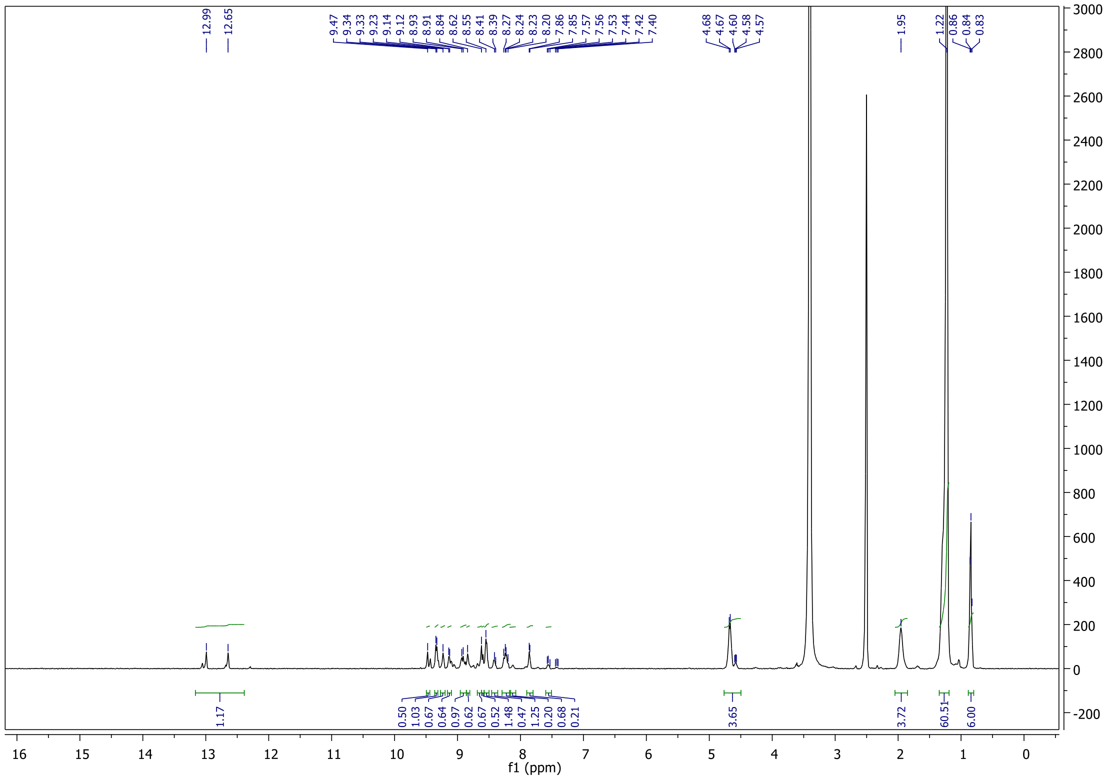

Figure S81. ^{1}H NMR of Compound **34**

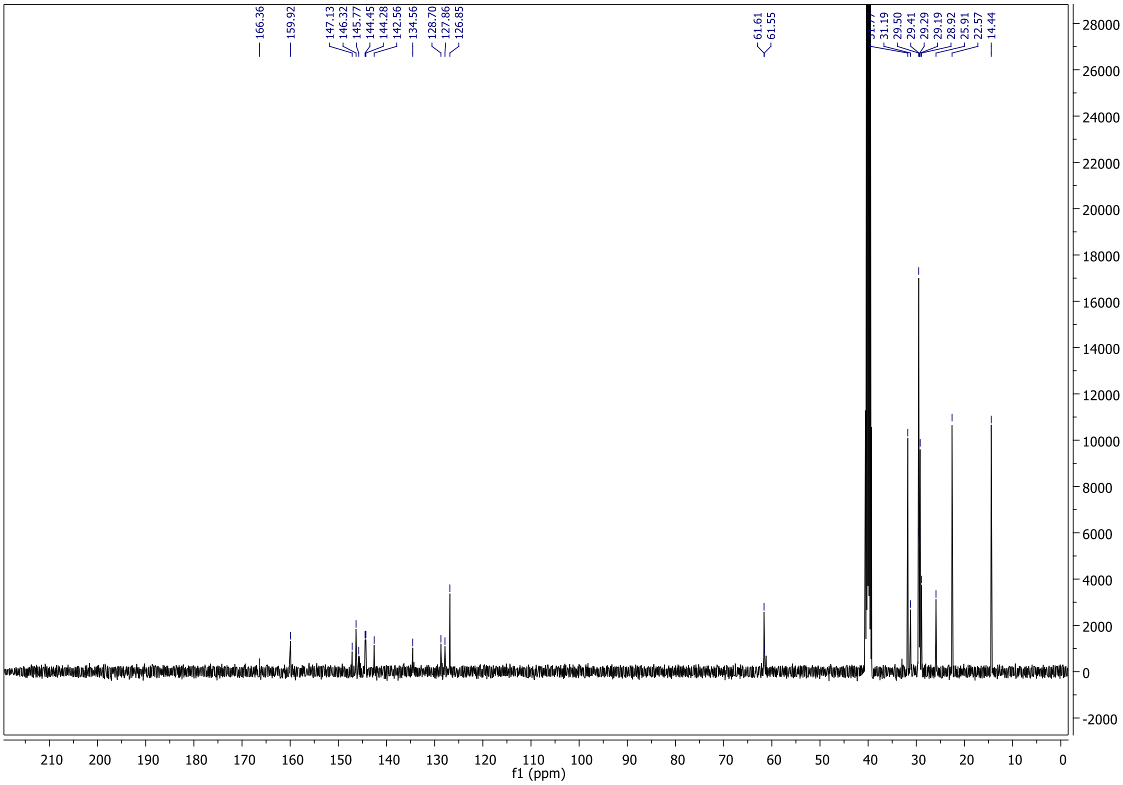

Figure S82. ^{13}C NMR of Compound **34**

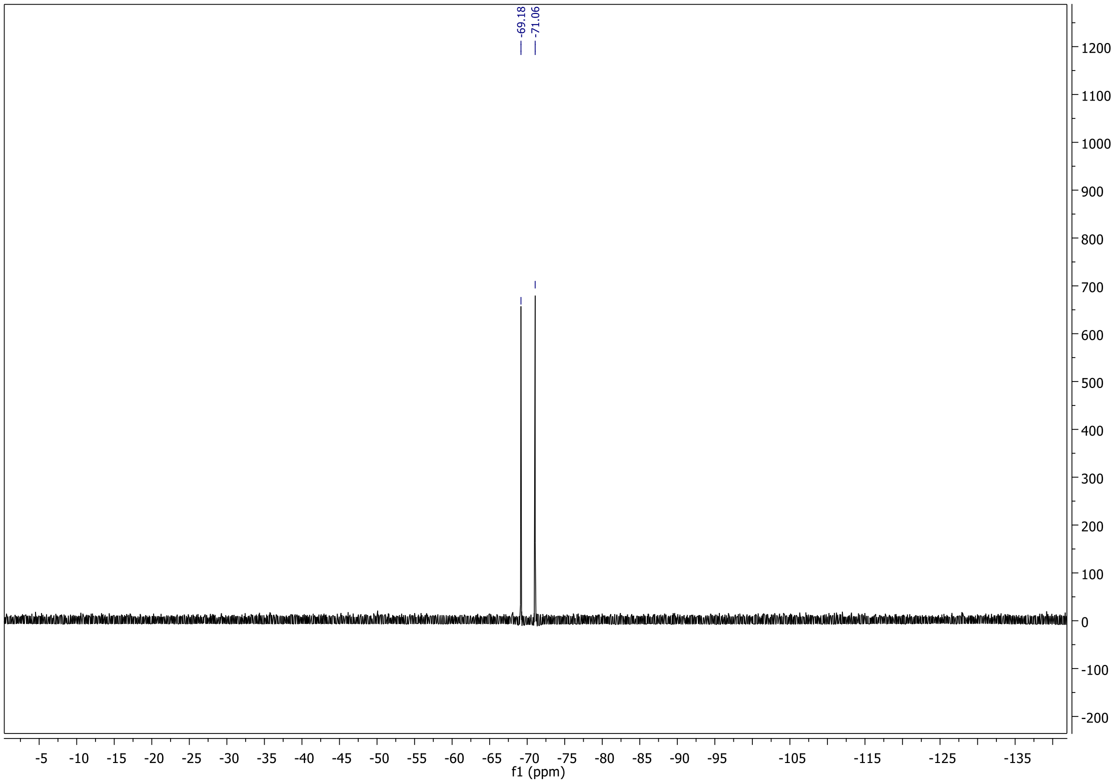

Figure S83. ^{19}F NMR of Compound **34**

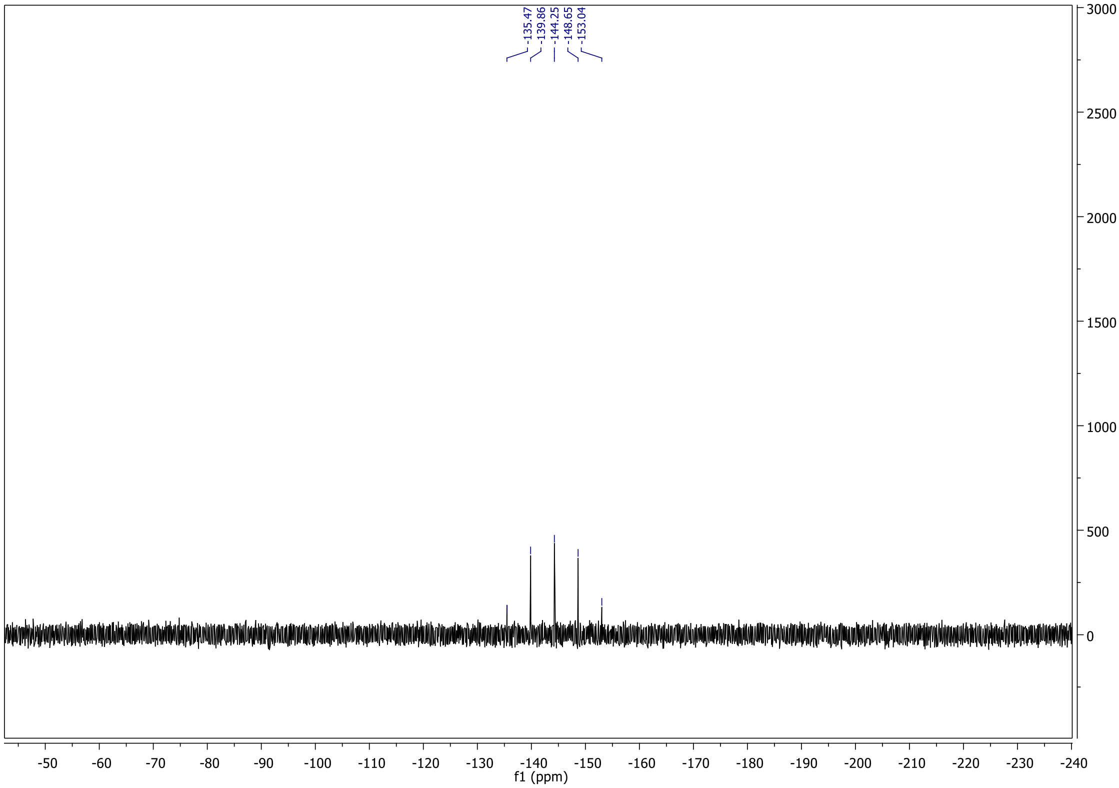

Figure S84. ^{31}P NMR of Compound **34**

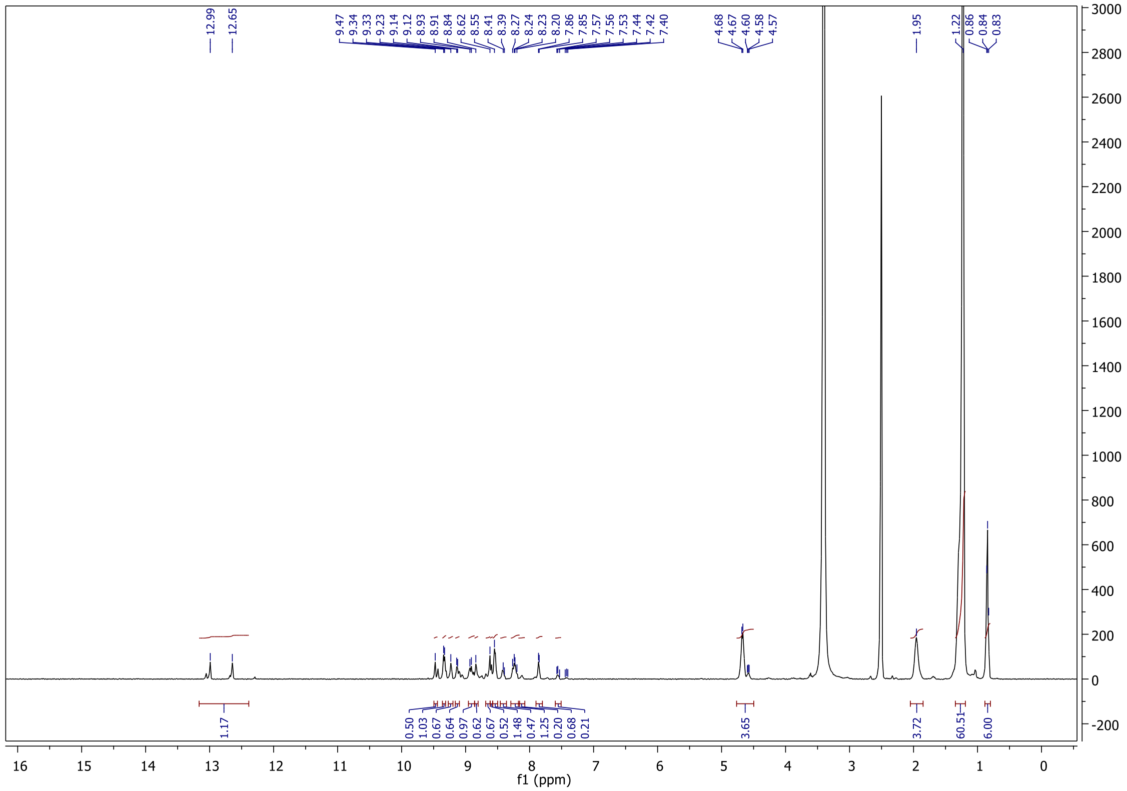

Figure S85. ^{1}H NMR of Compound **35**

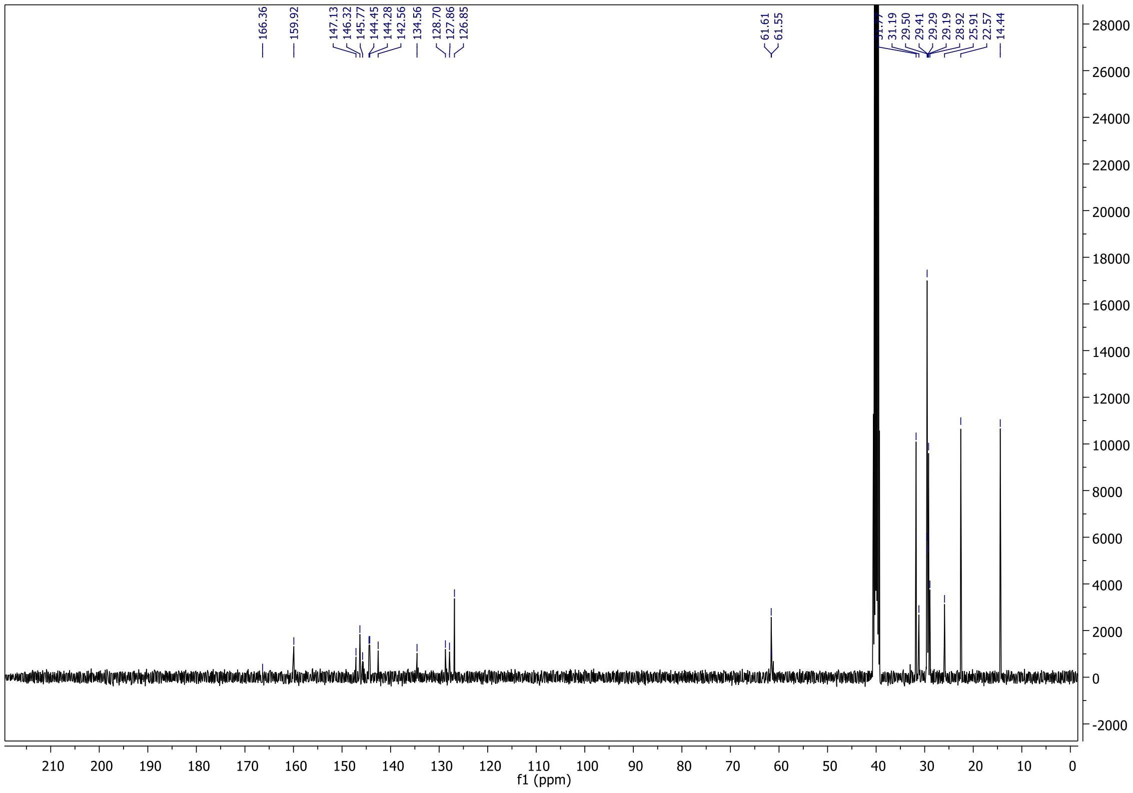

Figure S86. ^{13}C NMR of Compound **35**

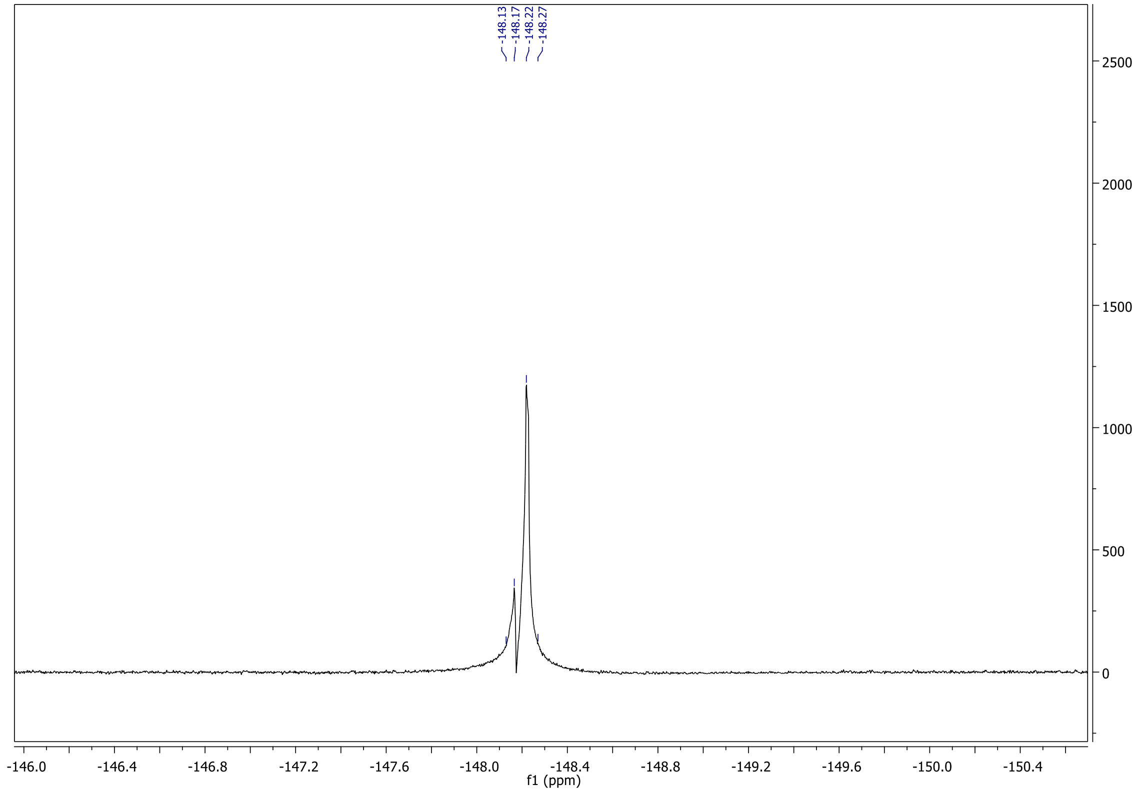

Figure S87. ^{19}F NMR of Compound **35**

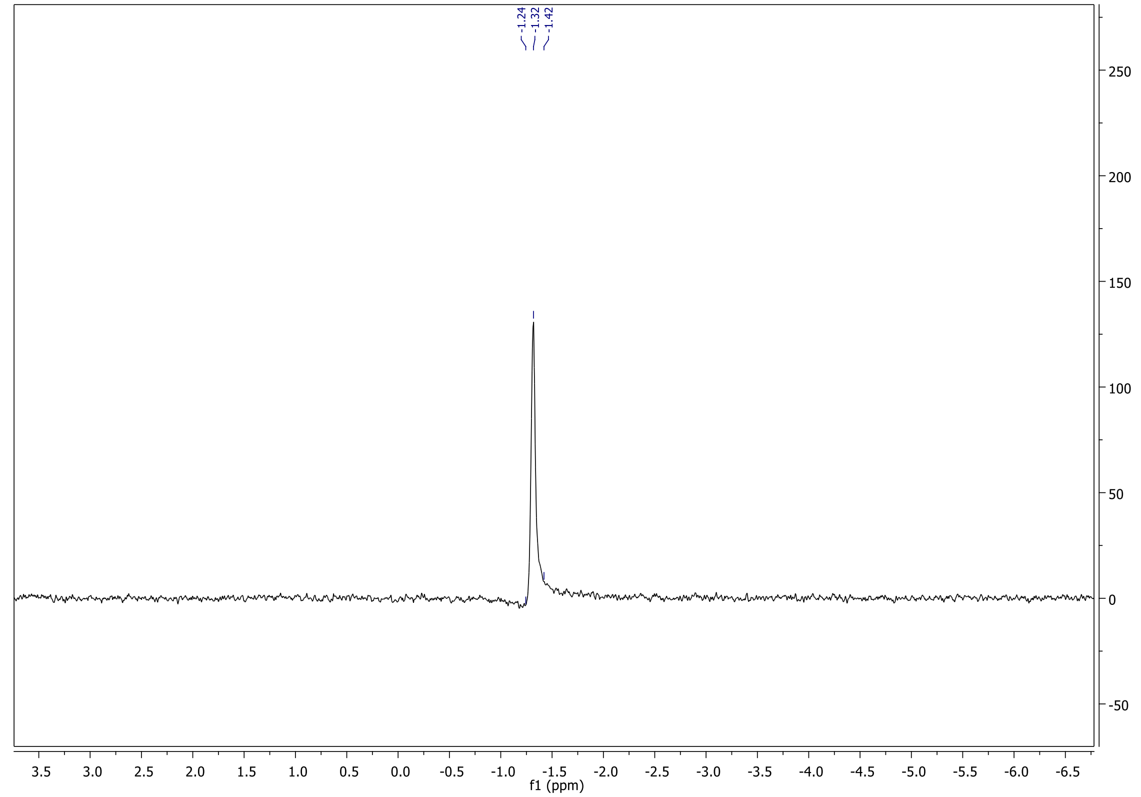

Figure S88. ^{11}B NMR of Compound **35**

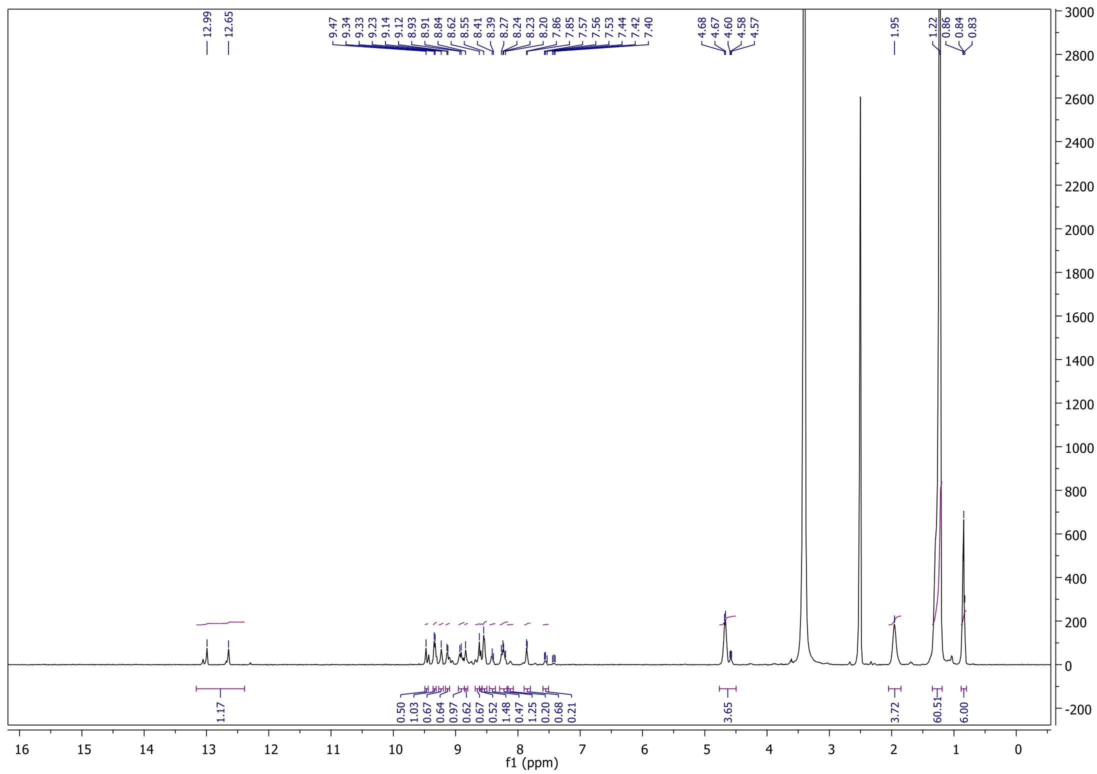

Figure S89. ^{1}H NMR of Compound **36**

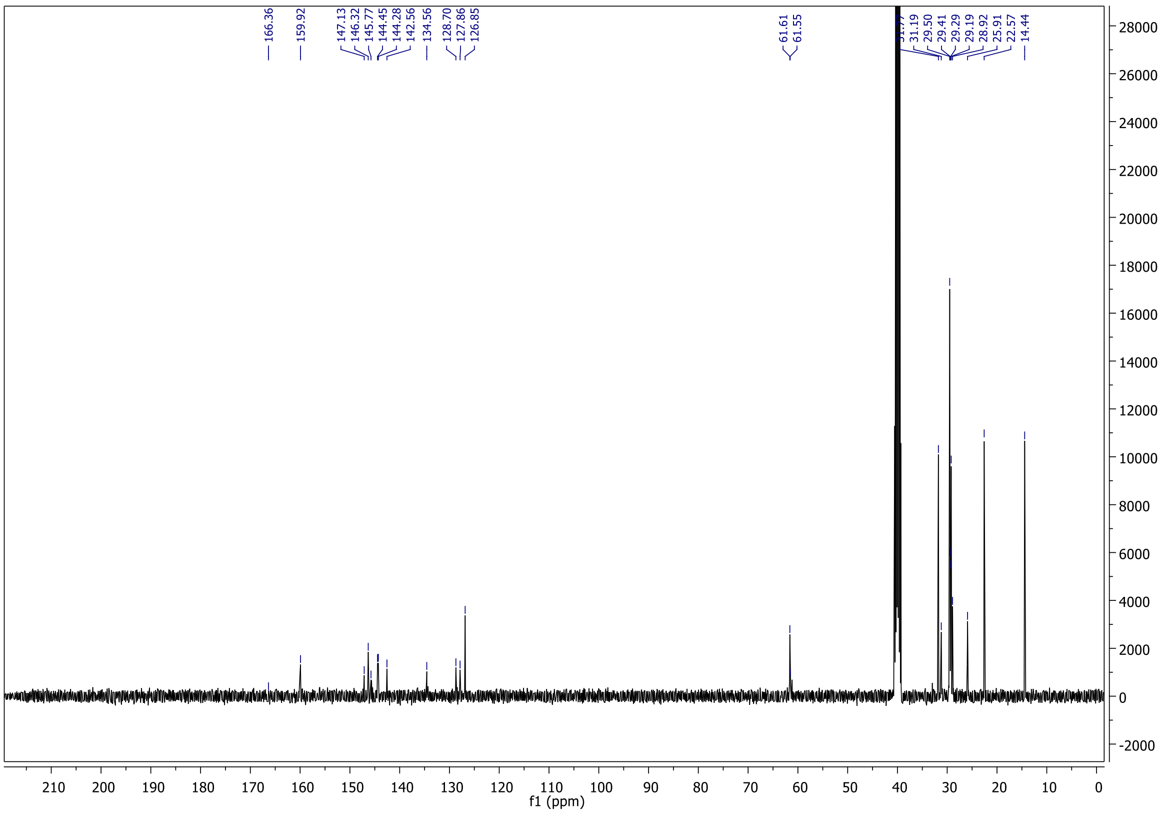

Figure S90. ^{13}C NMR of Compound **36**

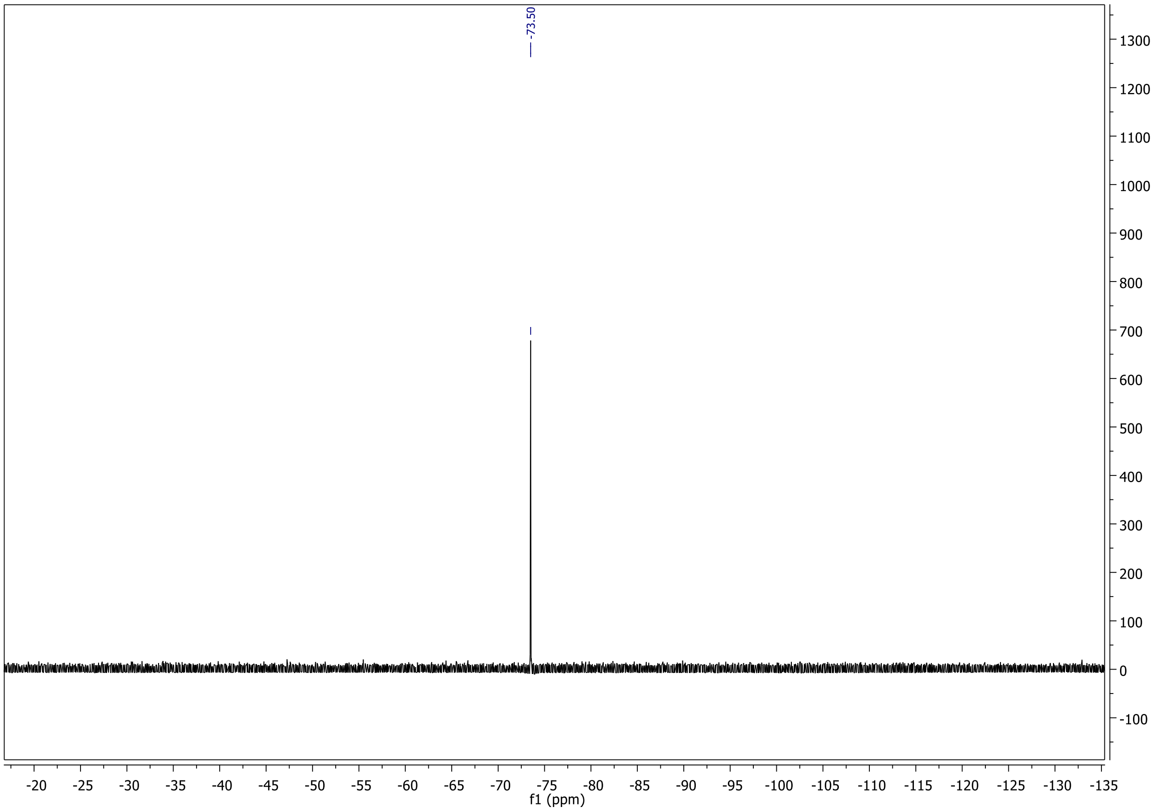

Figure S91. ^{19}F NMR of Compound **36**

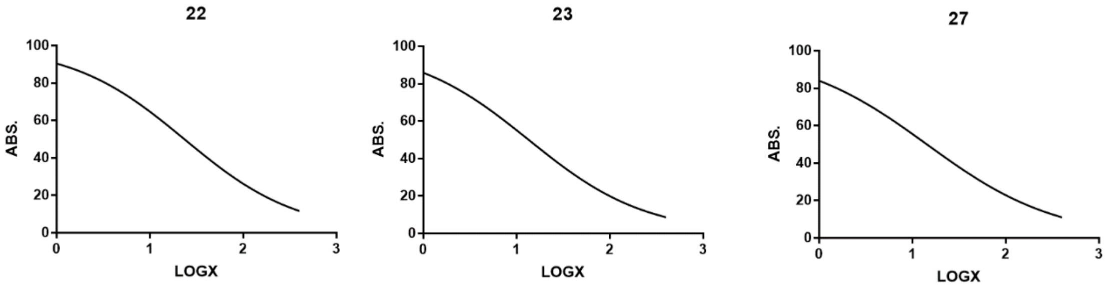

Figure S92.: Representative dose response curves
